# A rat liver cell atlas reveals intrahepatic myeloid heterogeneity

**DOI:** 10.1016/j.isci.2023.108213

**Published:** 2023-10-14

**Authors:** Delaram Pouyabahar, Sai W. Chung, Olivia I. Pezzutti, Catia T. Perciani, Xinle Wang, Xue-Zhong Ma, Chao Jiang, Damra Camat, Trevor Chung, Manmeet Sekhon, Justin Manuel, Xu-Chun Chen, Ian D. McGilvray, Sonya A. MacParland, Gary D. Bader

**Affiliations:** 1 Department of Molecular Genetics, University of Toronto, Toronto, ON, Canada; 2 The Donnelly Centre, University of Toronto, Toronto, ON, Canada; 3 Ajmera Transplant Centre, Toronto General Hospital Research Institute, Toronto, ON, Canada; 4 Department of Immunology, University of Toronto, Toronto, ON, Canada; 5 Department of Laboratory Medicine and Pathobiology, University of Toronto, Toronto, ON, Canada; 6 Department of Computer Science, University of Toronto, Toronto, ON, Canada; 7 Lunenfeld-Tanenbaum Research Institute, Toronto, ON, Canada; 8 Princess Margaret Research Institute, University Health Network, Toronto, ON, Canada; 9 The Multiscale Human Program, Canadian Institute for Advanced Research, Toronto, ON, Canada

**Keywords:** Components of the immune system, Transcriptomics, Model organism

## Abstract

The large size and vascular accessibility of the laboratory rat (*Rattus norvegicus*) make it an ideal hepatic animal model for diseases that require surgical manipulation. Often, the disease susceptibility and outcomes of inflammatory pathologies vary significantly between strains. This study uses single-cell transcriptomics to better understand the complex cellular network of the rat liver, as well as to unravel the cellular and molecular sources of inter-strain hepatic variation. We generated single-cell and single-nucleus transcriptomic maps of the livers of healthy Dark Agouti and Lewis rat strains and developed a factor analysis-based bioinformatics analysis pipeline to study data covariates, such as strain and batch. Using this approach, we discovered transcriptomic variation within the hepatocyte and myeloid populations that underlie distinct cell states between rat strains. This finding will help provide a reference for future investigations on strain-dependent outcomes of surgical experiment models.

## Highlights

• A multi-strain cellular atlas of the healthy rat liver
• Uncovering stain-based variations via interpretable matrix factorization
• Identifying key zonation signatures within the rat hepatic lobules

## Graphical abstract

**Figure:**
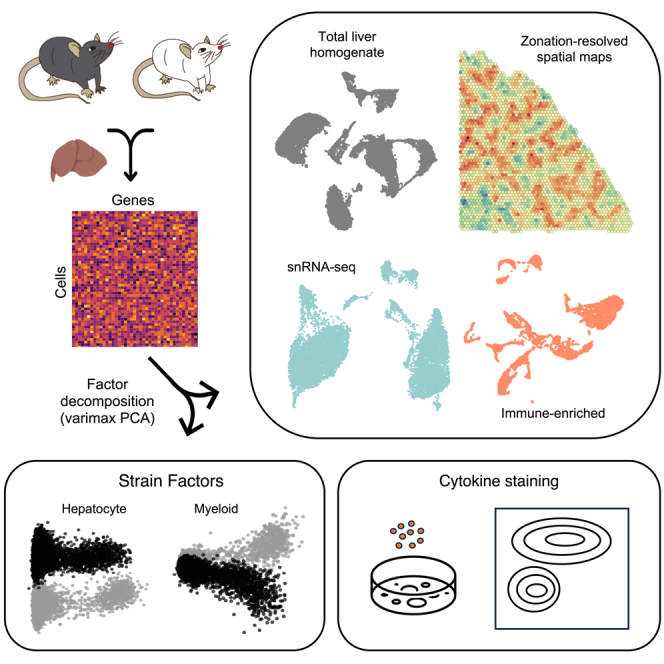

## Introduction

The liver is a multitasking organ that performs a remarkably diverse set of functions including nutrient metabolism, regulation of immune responses, and protein synthesis. Despite its highly regenerative and tolerogenic nature,^1^^,^^2^ inflammatory end-stage liver diseases such as drug-induced liver injury, hepatitis infection, hepatocellular carcinoma, and autoimmune hepatitis are common.^3^ Despite the recent advancements in medical strategies to treat acute liver disease,^4^^,^^5^^,^^6^ the development of therapeutic options is limited by our incomplete understanding of the cellular landscape of the liver in non-mouse animal models. The liver is composed of multiple cell types with complementary functions, including hepatocytes, biliary epithelial cells (cholangiocytes), mesenchymal cells (stellate cells and vascular smooth muscle cells [VSMCs]), myeloid cells, liver sinusoidal endothelial cells (LSECs) and multiple other immune cell populations.^7^ Hepatocytes make up the majority of liver volume and are involved in metabolism and drug detoxification, among other functions that are often zonated along the hepatic lobule.^8^^,^^9^ Myeloid cells are distributed throughout the liver and can adopt pro-inflammatory or anti-inflammatory roles, with phenotypic characteristics of recently recruited monocytic myeloid cells and more tissue-resident Kupffer cell-like populations, respectively.^7^ Current animal models used to recapitulate and study liver pathology include the porcine, murine, and rat models. A key advantage of the rat model (*Rattus norvegicus*) is its large size, which allows for better vascular access for disease models that include surgical interventions such as hepatectomies,^10^^,^^11^ hepatic ischemia reperfusion-induced injury models, transplant injury,^12^ and fibrocirrhotic bile duct ligation models.^13^^,^^14^

To date, our understanding of the rat liver has been informed by technologies such as bulk RNA sequencing (RNA-seq),^15^^,^^16^^,^^17^^,^^18^ transcriptome microarrays,^19^^,^^20^^,^^21^^,^^22^ immunohistology,^23^^,^^24^ targeted qPCR,^19^^,^^23^^,^^25^ and tandem mass spectrometry.^17^ These approaches have uncovered the presence of major expected hepatic populations in the rat liver;^19^ however, the relatively low resolution and targeted nature of these approaches do not allow us to have a holistic understanding of how the interaction between diverse hepatic cells shapes the liver environment. Single-cell RNA sequencing (scRNA-seq) technology is a powerful tool for the unbiased profiling of heterogeneous tissues. While both human^7^^,^^26^^,^^27^^,^^28^^,^^29^^,^^30^ and murine^31^^,^^32^^,^^33^^,^^34^ livers have been well studied at the single-cell level, the rat liver has remained poorly annotated. Studies using the rat model^13^^,^^35^^,^^36^ demonstrate strain-associated differences in the liver and inflammatory disease severity. For example, while both Dark Agouti (DA) and Lewis (LEW) strains are prone to Th1-skewed responses in the joints of treatment-induced autoimmunity models,^37^ DA rats appear to paradoxically have similar innate responses as autoimmune-resistant Albino Oxford (AO) rat strains.^38^^,^^39^ In the context of orthotopic transplantation, LEW recipient liver macrophages are better able to stimulate T cell proliferation in comparison to DA.^35^ This provides a rationale for single-cell examination of rat strain-specific differences at baseline.

We developed a single-cell transcriptomic atlas of the healthy liver, based on DA and LEW rats, to provide a reference atlas of the healthy rat liver. Our combined use of single-cell (sc) and single-nucleus (sn) RNA sequencing (RNA-seq) as well as spatial transcriptomics enabled us to discover cellular and molecular sources that drive the inter-strain variation and will be helpful for understanding strain-dependent hepatic disease models and rat liver biology in future studies.

## Results

### The cellular landscape of the healthy rat liver

**Figure 1:**
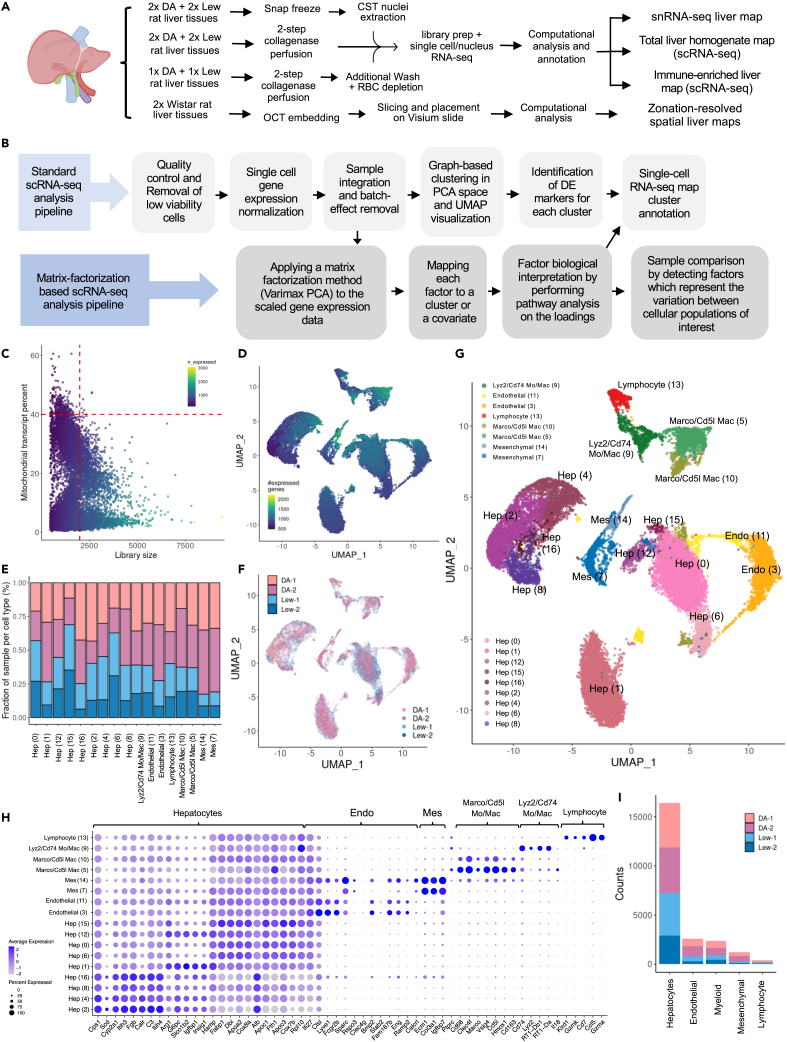
scRNA-seq profiling of rat liver reveals 17 distinct cell populations (A) Overview of single-cell RNA-seq pipeline, including both the experimental and analysis workflows. (B) Major steps of the standard and matrix factorization-based single-cell RNA-seq data analysis pipeline. (C) Viable cell selection for a Lewis rat liver sample (LEW-1) based on library size and mitochondrial transcript proportion shown as an example. High-quality cells were identified from the single-cell libraries having a minimum library size of 1500 transcripts and a maximum of 40% mitochondrial transcript proportion. (D) UMAP (uniform manifold approximation and projection for dimension reduction) plot of four rat samples including 2 samples from each Dark agouti (DA) and Lewis (LEW) rat strains. Cells are colored by the number of expressed genes, with lighter colors indicating higher gene counts. (E) Bar plot indicating the relative contribution of input samples to each cluster. All samples are represented in each cluster. (F) UMAP projection of cells labeled based on the input sample indicates that cells from different samples have been well-integrated and clusters represent cell-type differences rather than sample-specific variations. (G) UMAP projection of four total liver homogenate rat samples (each point represents a single cell) where cells that share similar transcriptome profiles are grouped by colors representing unsupervised clustering results. The legend indicates the unique color representing the cell-type annotation of each cluster. The cluster number is shown within the curved brackets. (H) Dot plot indicating the relative expression of marker genes in each population. The size of the circle indicates the percentage of cells in each population which express the marker of interest, and the color represents the average expression value of the marker. (I) The number of cells in each major cell type population colored by the contribution of each input sample. RBC: red blood cell, PCA: principal-component analysis, DE: differentially expressed, QC: quality control, Mac: macrophage, Mo: monocyte, Endo: endothelial, Mes: mesenchymal, Hep: hepatocyte.

We generated a multi-strain single-cell transcriptomic map of the healthy rat liver to help examine the cellular complexity in this model system. Single-cell transcriptomes were generated from total liver homogenates (TLHs) of four 16–18 week-old healthy male rats following 2-step collagenase digestion (Figure 1A). Two livers from each of the DA and LEW strains were sampled, and a standard scRNA-seq mapping pipeline was applied (Figure 1B). In total, 226,270 single cells were called by the 10x Genomics Cell Ranger software and 23,036 passed additional quality control filters and were included in the final map (see STAR Methods, Figures 1C, 1D, and S1; Tables S1 and S2). Significant batch effects were evident while integrating the four rat samples; therefore, the Harmony^40^ integration method was used to reduce the inter-sample technical confounding effects. After applying this batch correction, all clusters were represented by all animals, demonstrating that integration worked well (Figures 1E and 1F). Liver tissue from an additional two pairs of LEW and DA rats were processed for 10x Genomics snRNA-seq to further inform parenchymal cell identities (Figure 1A). These samples went through standard quality control steps (see STAR Methods; Figure S2; Table S1) and were batch-corrected using the Harmony integration tool. The resulting map contained 12,497 nuclei. Cell populations were annotated, based on known marker genes, using top differentially expressed (DE) genes^41^ (see STAR Methods; Figures 1G and 1H; Tables S3 and S4). To resolve the spatial distribution of rat hepatic cell populations, we conducted spatial transcriptomics on two healthy Wistar rat liver samples using 10x Genomics Visium technology. These samples were then quality controlled in a similar manner to scRNA-seq data (Figures S3; Table S1).

### Hepatocytes

**Figure 2:**
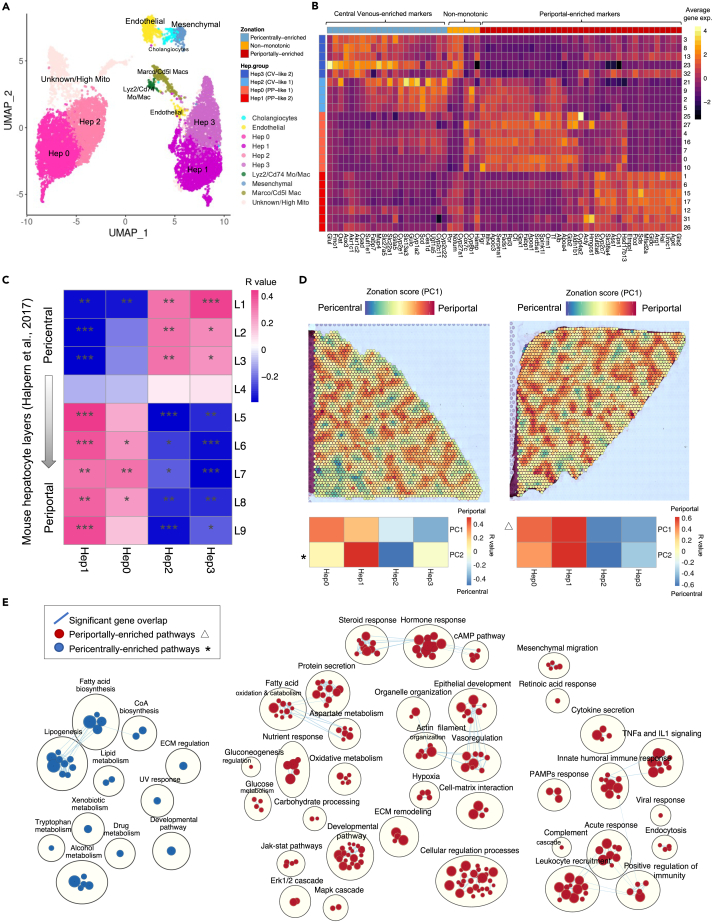
SnRNA-seq and spatial transcriptomic profiling of the rat liver resolves hepatocyte zonation Four additional rat liver samples were added and sequenced using snRNA-seq to better characterize hepatocyte and cholangiocyte populations and verify the strain variations identified based on the scRNA-seq TLH map. (A) UMAP projection of four snRNA-seq samples where cells are colored based on cell-type annotation. (B) Heatmap representing the average gene expression of zonated genes based on spatial data within the snRNA-seq clusters. (C) Pearson correlation between the average gene expression of the genes across snRNA-seq hepatocyte clusters and the nine layers of mouse liver cells was calculated (see STAR Methods). Mouse liver layer-9 is more periportal and layer 1 is pericentral. Red represents a positive correlation, and blue represents a negative correlation. (∗: p value <0.05, ∗∗: p value <0.01, ∗∗∗: p value <0.001). (D) Projection of zonation signature scores, captured by PC1, across the spatial transcriptomics spots of two healthy Wistar rat liver cryosections. The top negatively loaded genes in PC2 (and PC1) of both samples are enriched in pericentral markers, and the top positively loaded genes in PC1 (and PC2) factors are enriched in periportal markers. Red and blue represent high periportal and pericentral zonation scores, respectively. The two heatmaps represent the Pearson correlation between the zonation factors PC1 and PC2 and the average expression of snRNA-seq hepatocyte clusters. Both PC1 and PC2 are positively correlated with PP-like clusters Hep0 and Hep1 and negatively correlated with CV-like clusters Hep2 and Hep3. The asterisk and triangle symbols indicate the factors used for pathway enrichment analysis. (E) Pathway enrichment analysis using GSEA (gene set enrichment analysis) to examine active cellular pathways in periportal and central venous regions of the healthy rat liver based on spatial PC1 and PC2 loadings visualized as an enrichment map. The pathways enriched in the pericentral and periportal areas are based on PC2 (asterisk) of liver cryosections-A (left) and PC1 (triangle) of liver cryosections-B (right) respectively. Each circle represents a gene ontology (GO) biological process term. The size of the circles represents the number of genes in that pathway and blue lines indicate significant gene overlap.

Hepatocytes, organized in functional units referred to as lobules, make up the majority of the liver volume (Figure 1I). Many of their critical biological functions are zonated based on their spatial organization from the center of the lobule near the central vein (CV) to the outer regions near the portal triad. Data from both sc- and snRNA-seq protocols identify hepatocyte-like clusters, based on their correlation with hepatocytes of the mouse liver atlas (Figure S4), and expression of hallmark hepatocyte markers without high expression of immune endothelial and mesenchymal genes (Figures 1G and 2A–2E).

Comparative gene expression analysis of our data to a bulk RNA-seq dataset of laser capture microdissected zonated regions of the healthy mouse liver lobule^42^ revealed poor zonated marker distribution in the scRNA-seq dataset compared to snRNA-seq (Figure 2C), as has been observed before.^27^^,^^43^ To further resolve hepatocyte cluster identity, the datasets were compared with a spatial transcriptomics map of the rat liver from two Wistar rats. Principal-component analysis (PCA)^44^ of these samples revealed the largest dimension of variation was related to lobule zonation (Table S5). PC1 and PC2 in both samples indicate clear histological periportal to central venous zonation patterns (Figures 2D and S5). Additionally, key periportal human (*Tf*, *Hmgscs1*^27^^,^^43^) and mouse (*Ass1*, *Arg1*^42^) genes as well as periportal markers from rat proteomic studies (*Gls2*, *Srd5a1*, *Orm1*^45^) are positively enriched in PC1 and PC2. Known pericentral markers (*Oat*, *Sult1e1*, *Cyp2e1*, *Glul*^27^^,^^42^^,^^43^) are negatively enriched, reinforcing that these principal components represent zonation patterns^45^ (Figures S6 and S7; Data Portal). Pathway enrichment analysis of the PCs was performed to further validate that PC1 and PC2 represent zonation features. Periportal-biased processes such as immunity, angiogenesis, lipid beta-oxidation, fatty acid catabolism, and gluconeogenesis regulation^8^^,^^46^ are found in the positive side of PC1 while the negative side of PC2 is enriched in pericentrally biased metabolic processes, such as lipogenesis and various steroid and xenobiotic metabolic processes^9^ (Figure 2E). Examination of key markers and correlation analysis between PC1 and PC2 of the spatial transcriptomics data and snRNA-seq hepatocyte clusters shows a clear presence of periportal and pericentral hepatocyte populations (Figures 2B–2D). These findings suggest that pericentral and periportal programming is well preserved across species.

### Mesenchymal cells

The hepatic mesenchymal fraction includes populations such as hepatic stellate cells (HSCs), VSMCs, and fibroblasts (FBs).^27^ Mesenchymal cells anatomically reside between sinusoidal endothelial cells and hepatocytes and are involved in vitamin A storage, extracellular matrices (ECMs) synthesis, maintenance of hepatocyte function,^47^ and regulation of sinusoidal circulation.^48^ These populations also help regulate immune responses during inflammation,^1^ but upon activation can also be a source of maladaptive extracellular matrix deposition, as in the case with liver fibrosis.^49^

**Figure 3:**
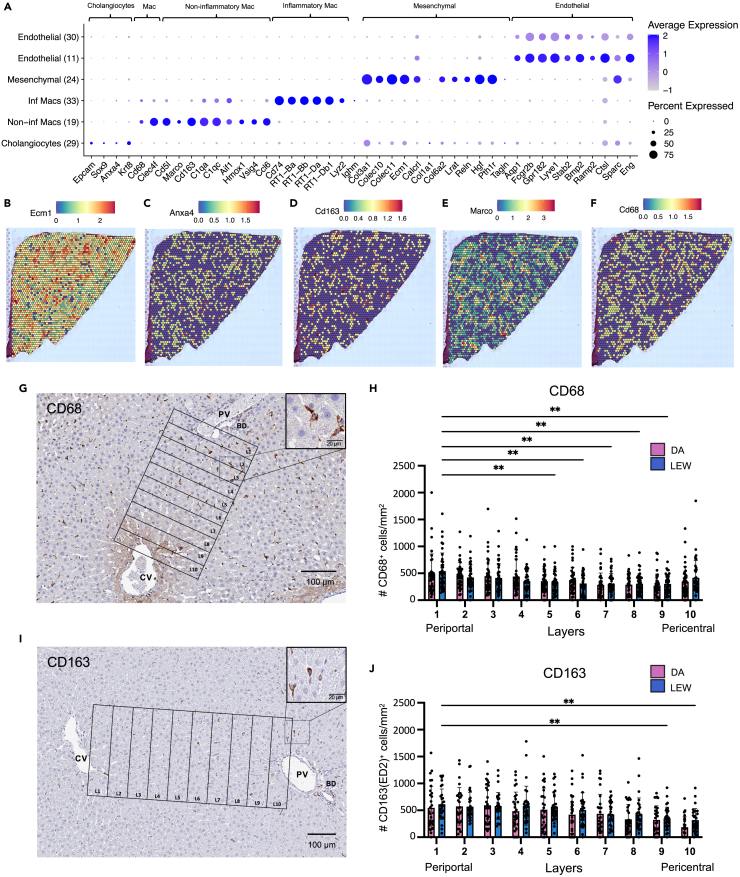
Comparisons between transcriptomic platforms and immunohistochemistry suggest zonation patterns of selected hepatic populations To provide information on the zonation of hepatic populations within hepatic lobules, key cluster markers of each cell type were examined in PC1 and PC2 of each spatial sample. (A) Dot plot indicating the relative expression of marker genes in each population of the snRNA-seq map. The size of the circle indicates the percentage of cells expressing the marker of interest, and the color represents the average expression value of the marker. Expression values of (B) mesenchymal marker *Ecm1* (C) cholangiocyte marker *Anxa4* (D) myeloid marker *Cd163* (E) non-inflammatory myeloid marker *Marco* (F) myeloid marker *Cd68*. Red and dark blue indicate higher and lower expression values in each spot, respectively. (G) Representative spatial distributions of CD68^+^ cells in the rat liver lobule. Rectangular layers 350 um wide were drawn from the portal tract (layer 1) to the central vein (layer 10) region. Digital images were scanned at 20× magnification. The scale bar represents 100 μm in the full image and 20 μm in the enhanced area. Each rectangular layer is referred to as a region of interest (ROI). (H) Quantification of CD68^+^ cell densities (#CD68+ cells/layer mm2) in the liver lobule for DA and LEW rats. 30 ROIs were assessed per strain across three animals. A higher number of CD68^+^ cells were detected near the periportal area. No significant strain-specific differences in the spatial distribution of CD68^+^ cells were noted. (I) Representative spatial distributions of CD163+ cells in the rat liver lobule. (J) Quantification of CD163+ cell densities (#CD163+ cells/layer mm2) in the liver lobule for DA and LEW rats. The statistical analysis reflects similar strain and zonation patterns as CD68. Statistical significance was determined using a two-way ANOVA followed by Sidak’s multiple comparisons test. (∗: p value <0.05, ∗∗: p value <0.01, ∗∗∗: p value <0.001,∗∗∗∗: p value <0.0001). Data are represented as mean ± SEM. Each dot in H and J represents an ROI region (n = 30). ROI: region of interest, BD: bile duct, CV: central vein, PV: portal vein.

We annotated two clusters in our scRNA-seq map (scClusters 7 and 14) and one cluster (snCluster 24) in our snRNA-seq map, as mesenchymal-like based on DE genes including extracellular matrix proteins (*Ecm1)* and type III collagen alpha 1 (*Col3a1*) (Figures 1G, 1H, 2A, and 3A) which are essential to the role of HSCs in extracellular matrix deposition and have previously been described as mesenchymal genes^27^^,^^43^ (expanded markers shown in Figure S8).

To increase resolution, mesenchymal-like clusters were subclustered and correlation analysis was performed with mouse scRNA-seq data from sorted *Pdgfrb*+ cells found in Dobie et al., 2019^50^ (Figures S9A and S9B). ScRNA-seq subclusters that appear to be derived from contaminating non-mesenchymal populations (3, 2) and expressing top myeloid (*Cd68*, *Clec4f*) and endothelial (*Lyve1*, *Fam167b*) DE genes were excluded. ScMes-5, scMes-4, and snMes-1 were denoted as FB-like due to correlation with mouse liver FBs (Figures S9C and S9D) and the expression of mouse FB genes (*Dpt*, *Enpt2*, *Col1a1*, *Col4a1*, and *Gsn*^50^) (Figures S9E and S9F). ScMes-5 expressed additional smooth muscle genes (*Acta2*, *Fn1*, *Sparcl1*, *Tagln*, and *Tpm2*^43^^,^^50^) (Figures S9E and S9F) but did not correlate strongly with known VSMC clusters, suggesting this population may be a mixed population with liver FB-like and activated myofibroblast-like cells. Expression of active pathways in retinol storage in scMes-1, scMes-0, and snMes-0 and positive correlation with mouse HSC clusters (Figures S9C–S9F) suggest that these clusters predominantly represent quiescent HSC-like populations.^43^^,^^50^ However, snMes-0 expressed additional myofibroblast-associated genes (*Acta2*, *Tagln*) (Figures S9E and S9F), suggesting there might also be myofibroblast-like cells found within this cluster. Finally, snMes-2 was not enriched for any particular gene set and is of unknown identity. Interestingly, spatial transcriptomics revealed zonation of key mesenchymal (*Ecm1*, *Col3a1*) and HSC genes (*Pth1r*, *Lrat*) to be negatively enriched in PC1/2 and highly concentrated in pericentral areas (Figure 3B; Data Portal).^43^

### Endothelial cells

The hepatic endothelium consists of LSECs and vascular endothelium (portal and central venous endothelium). LSECs are a specialized endothelial population that line the hepatic sinusoids and contribute to the regulation of hepatic blood pressure, nutrient uptake, and the maintenance of HSC quiescence.^51^^,^^52^ Immunohistochemical staining in mice has described general endothelial cells in the liver as expressing high levels of *Cd31* (*Pecam*) and *Cd103* (*Eng*), periportal LSECs as expressing high levels of *Cd36*, with low levels of *Lyve1*, and central venous LSECs as expressing high levels of *Cd32b* and *Lyve1*.^53^^,^^54^ However, in rats, endothelial zonation has yet to be reported.

We identified two populations of *Ptprc*^−^ cells in the scRNA-seq map (scClusters 3 and 11) and two populations in the snRNA-seq map (snCluster 11 and 30). These populations were annotated as endothelial-enriched based on the expression of *Calcrl* and *Ramp2*, which is involved in adrenomedullin signaling pathways^55^ (Figures 1H, 2A, 3A, and S10A). ScCluster 3, the most abundant endothelial cell population, was characterized by enriched expression of *Lyve1*, *Fcgr2b*, *Sparc*, and *Stab2* (Figures 1H and S10A) with little expression of *Vwf* (Figure S10A) suggesting an LSEC identity. By correlation analysis, both scClusters 3 and 11 were similar to mouse sinusoidal, inflammatory, and cycling endothelial populations (Figure S4). These clusters did not show differential expression of known zonated endothelial genes such as *Rspo3*^56^ and *Clec4g*, and both clusters expressed high levels of *Fcgr2b* (known to be enriched in CV LSECs^54^) and *Aqp1* (known to be enriched in periportal LSECs^57^) (Data Portal). Endothelial genes such as *Lyve1* and *Vwf* found in scCluster 11 were also found snClusters 11 and 30 (Figure 2A; Data Portal). SnRNA-seq endothelial populations were subclustered for increased resolution, and comparisons were made to our spatial transcriptomic data (Figure S10B). The resulting subcluster 3 had a stronger expression of PC1-enriched periportal markers (*Vwf* and *Ltbp4*) with little expression of *Lyve1*,^58^ while subcluster 1 and 0 expressed higher pericentral-associated genes such as *Lyve1*, *Fcgr2b*, and *Bmp2* (Figures S10C and S10D; Data Portal). Further examination of other known periportal markers in our spatial transcriptomics data did not reveal clear endothelial zonation patterns (Figure S10D; Data Portal), perhaps due to the low capture of endothelial genes by the Visium spatial transcriptomics platform.^43^

### Biliary epithelial cells

Cholangiocytes are liver-specific biliary epithelial cells whose primary function is the production and modification of bile as it flows along the biliary tract.^59^ In line with previous literature, cholangiocytes were poorly captured with scRNA-seq and were only detected by our snRNA-seq map.^43^ SnCluster 29 of the snRNA-seq map was identified as being enriched in the expression of *Epcam*, *Sox9*, *Anpep*, and *Anxa4*,^43^ resulting in a total of 108 cholangiocyte-like cells with *Anxa4* and *Epcam* showing a periportal distribution on the spatial transcriptomics map (Figures 3A and 3C).

### Myeloid cells

The liver contains more resident myeloid cells than any other solid organ in the body.^60^ Tissue-resident myeloid cells exhibit immense phenotypic plasticity and can perform a diverse set of functions. Depending on the local immune microenvironment and external stimuli, bone marrow-derived monocytes can be recruited to the liver, where they participate in both liver injury and tissue repair. In comparison, the primary function of sessile resident myeloid cells is to clear debris, in addition to mediating the tolerogenic environment of the liver in the steady state.^61^^,^^62^

Our single-cell analysis revealed multiple clusters of *Cd68*^+^ myeloid-enriched cells. *Cd68*^+^ myeloid scClusters 5 and 10 were characterized by enriched expression of *Marco*, *Vsig4*, *Cd5l*, *Cd163*, and *Hmox1* (Figure 1H, see extended gene expression in Figure S11). These clusters appear to be more Kupffer cell-like due to the expression of key genes (*Marco*, *Cd5l*, *Clec4f*) which have been previously described to annotate more tissue-resident myeloid populations.^63^ Specifically, *Vsig4* is a co-inhibitory ligand that has a hepatoprotective role in maintaining the intrahepatic tolerance required to suppress triggered immune responses^64^^,^^65^ and has been shown to be highly expressed in murine Kupffer cells (KCs),^64^^,^^65^ as well as being a core KC gene in pig and macaque KCs.^34^ These findings may suggest a tolerogenic role of *Marco*^+^*Cd5l*^*+*^*Cd68*^*+*^ cells, which are represented by snCluster 19 of our snRNA-seq map (Figure 3A). Our analysis of scCluster 9 revealed a mixed cluster of *Ptprc+* immune cells enriched for *Cd68*^*+*^myeloid cells (enrichment and subclustering of immune cells are discussed in the following). ScCluster 9 is enriched in described macrophage and monocyte markers (*Cd68*, *Cd74*, *Lyz2*, and major histocompatibility complex (MHC) class 1-related genes) without the expression of *Vsig4* and *Marco*, suggesting that it is enriched for recently recruited macrophage/monocyte populations. However, ScCluster 9 also contains additional immune populations such as T cells (*Cd3e*), conventional denditic cells (cDCs) cDC1s (*Clec9a*, *Xcr1*, *Batf3*, *Irf8*) (Figure S11), cDC2s (*Clec10a*, *Irf4*, *Sirpa*), and plasmacytoid dendritic cells [pDCs] (*Siglech*). This macrophage/monocyte cluster was represented by snCluster 33 of our snRNA-seq dataset, but due to lower capture of non-myeloid immune cells by snRNA-seq technologies,^43^ it contains only a minor *Ighm*^+^ B cell population (Figure 3A). To resolve zonation, an examination of key myeloid markers (*Cd68*, *Cd163*) and key genes of KC-like myeloid cluster genes (*Cd5l*, *Marco*, *Aif1*, *Hmox1*, *Clec4f*) in PC1 was performed. The positive enrichment in PC1 suggests the presence of myeloid cells is skewed toward the periportal areas (Figures 3D–3F; Data Portal). To validate this enrichment, quantification of immunohistochemistry stainings of *Cd163*, *Cd68*, and *Hmox1* was performed using a publicly available QuPath-based image analysis protocol.^66^^,^^67^ This analysis confirms the periportal-biased nature of non-inflammatory myeloid cells (Figures 3G–3J and S12).

### Varimax PCA analysis uncovers biological sources of variation between rat strains

**Figure 4:**
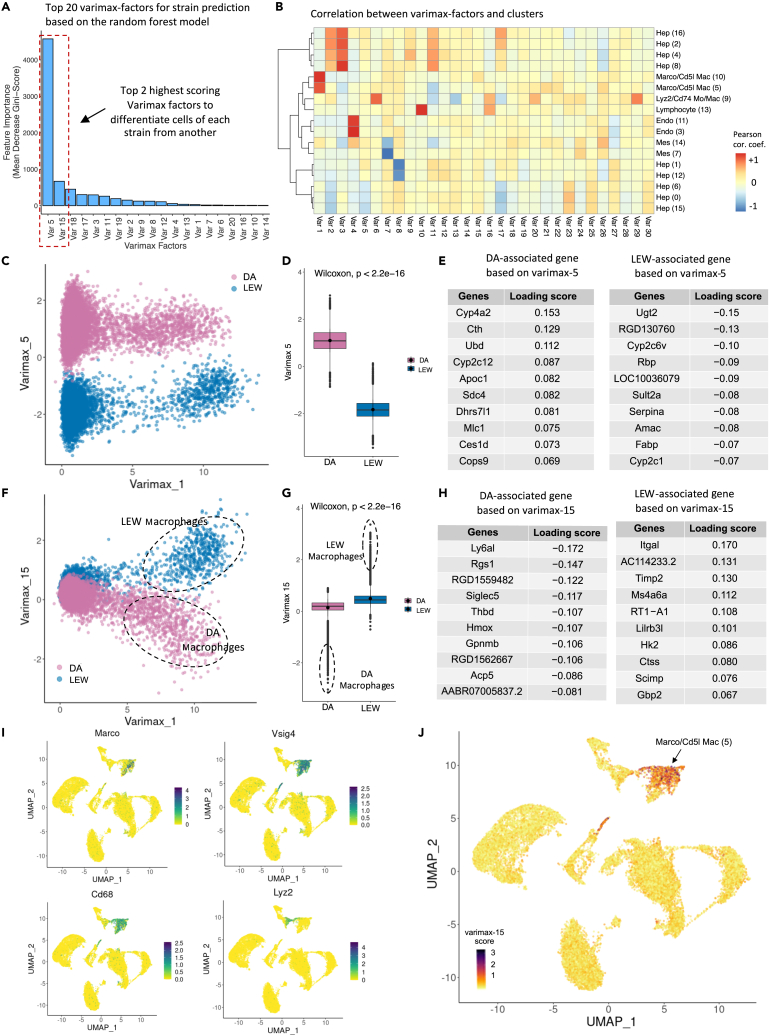
Varimax PCs capture rat hepatic cell identity signatures and strain-specific differences (A) Bar plot representing the feature importance scores (mean decrease Gini impurity) of the top 20 features (varimax factors) of the random forest model trained to predict the strain attributes of the rat hepatic cells. Varimax PC5 and 15 are the most informative features to differentiate cells of each strain from another, which indicates the two factors have captured strain-related variations within the map. (B) A correlation heatmap between the average gene expression of each cluster and the loading scores of varimax factors (capturing the contribution of all genes to a factor). Columns are varimax factors and rows are cell populations. Each cell-type cluster is defined by key marker genes, and dark red or blue indicates that the expression of a marker gene set is positively or negatively correlated, respectively, with a particular varimax factor. A high absolute correlation value indicates a match between a varimax factor and a cell-type cluster. (C) The projection of cells over varimax-1 and 5 indicates that the cells from each strain form distinct clusters over varimax-5. (D) Boxplot indicating the distribution of varimax-5 score over each strain. Cells from DA and LEW strains represent significantly different varimax-5 scores (Wilcoxon-test p value <2.2e-16), indicating that varimax-5 has captured strain differences. (E) The top 10 genes on the top (left table) and bottom (right table) of the varimax-5 loading list mainly contain known hepatocyte markers, indicating that varimax-5 has captured hepatocyte-specific strain differences. Genes with high positive scores (left table) are associated with the DA strain and genes indicating negative loading scores (right table) are LEW-related. The absolute loading scores indicate the contribution of each gene to the corresponding factor. (F) Projection of cells over varimax-1 and 15 indicates that a population of cells from each strain (dotted lines) forms distinct clusters over varimax-15. Annotation of the selected cells indicates that they are mainly from the *Marco*+ myeloid cluster 5. (G) Boxplot indicating the distribution of hepatic cells based on strain over varimax-15. (Wilcoxon-test p value <2.2e-16). The outlier data points (dotted lines) are mainly myeloid cells. (H) The top 10 genes with positive (right table) and negative (left table) varimax-15 loading scores are immune-response related. Genes with positive scores (right table) are associated with the LEW strain, and genes indicating negative loading values (left table) are DA related. The absolute loading scores indicate the contribution of each gene to the corresponding factor. (I) Expression pattern of known myeloid marker genes *Marco*, *Vsig4*, *Cd68*, and *Lyz2* over UMAP. Dark green represents high expression values. The distribution of general myeloid markers (*Cd68*, *Vsig4*) and non-inflammatory myeloid marker (*Marco*) is consistent with the varimax-15 distribution (Figure 2J). (J) The UMAP projection of cells colored based on the varimax-15 score shows the enrichment of varimax-15 over *Marco*^+^ myeloid population (cluster 5). Darker colors represent higher values of varimax-15 scores. Data are represented as mean ± SEM with each dot representing a single cell. Corrcoef.: correlation coefficient, Var: varimax PC. varimax PCs are referred to as PCs within the main text.

To better understand strain-specific differences in our map, we applied varimax PCA,^44^^,^^68^^,^^69^ a matrix factorization method, to separate DA and LEW signals (principal components, or factors) in the data from other signals for further interpretation (Figure 1B, Figure 4, Table S5). To identify factors that can explain strain-specific differences, we used a random forest to predict strain labels from the factors identified per cell and discovered the factors most important for the strain label classification (Figure 4A). We also identified principal components that explain cell-type signals using correlation analysis (Figures 4B and S13). The resulting factors were interpreted using pathway and gene set enrichment analysis (see STAR Methods). Using this approach, two main strain-specific factors (varimax PC5 and 15) were identified (Figures 4A and S14) within the scRNA-seq TLH map. The strongest strain-specific signal is observed with varimax PC5, which affects all cells in the data (Figures 4C and 4D). Genes with the strongest association with this factor are hepatocyte markers (*Apoc1*, *Fabp1*, and Cytochrome p450 genes), suggesting that this factor mainly represents strain variations within the hepatocyte populations (Figure 4E). The global association of this factor with all cells in the scRNA-seq dataset is likely a cell-dissociation procedure artifact caused by fragile hepatocytes leaking RNA into the cell homogenate before sequencing (Figure S15).^43^ DA strain-associated genes in this factor are enriched in nuclear receptors, such as *Hnf4a*, *Pparg*, and *Esr1* (Table S6) (Figure S16). *Pparg* promotes *de novo* lipogenesis and fat accumulation in hepatocytes.^70^^,^^71^ This hepatocyte-specific strain signal was confirmed in the snRNA-seq dataset (Figure S17; Table S5). The second-strongest strain-specific signal is varimax PC15, which is mainly associated with myeloid populations of both rat strains (Figures 4F and 4G), as confirmed by the genes with the strongest association with this factor (Figure 4H), the expression pattern of *Marco*, *Visg4*, *Cd68*, and *Lyz2* marker genes (Figure 4I), and the correlation with myeloid cells in our map (Figure 4J).

**Figure 5:**
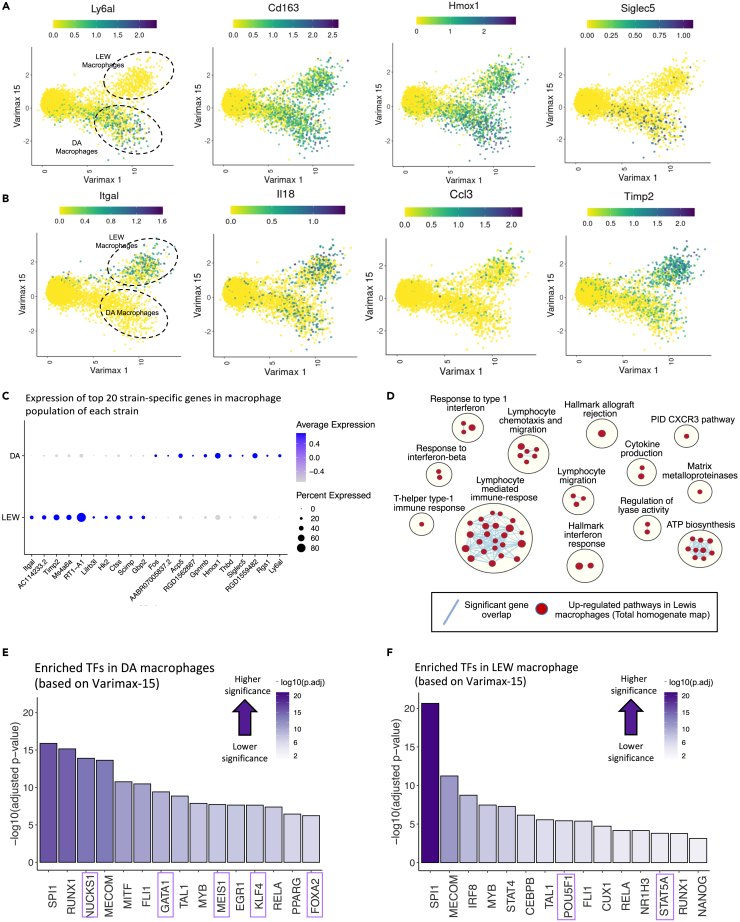
Strain-specific differences are found in intrahepatic myeloid cells (A) Expression pattern of the top DA-enriched genes (*Ly6al*, *Cd163*, *Hmox1*, *Siglec5*) over PC15 and 1. LEW and DA myeloid cells have been marked with dotted circles. Dark green indicates higher expression values. Comparison with Figure 2F confirms that the selected genes have higher expression in the DA strain compared to LEW. (B) Expression pattern of the top LEW-enriched genes (*Itgal*, *Il18*, *Ccl3*, *Timp2*) over varimax-15 and 1. Comparison with Figure 2F confirms that the selected genes have higher expression in the LEW strain compared to DA. (C) Dot plot indicating the relative expression of strain-related genes within the myeloid fraction (clusters 5, 10, 9) of each strain. The top 10 genes with positive (LEW-associated) and negative (DA-associated) varimax-15 loading scores have been selected. The size of the circle indicates the percentage of cells in each population expressing the marker, and its color shows the average expression value. (D) Pathway enrichment analysis using GSEA (gene set enrichment analysis) to examine active cellular pathways in LEW vs. DA myeloid cells based on varimax-15 loadings visualized as an enrichment map. Each circle represents a gene ontology (GO) biological process term. The size of the circles represents the number of genes in that pathway, and blue lines indicate significant gene overlap. Since PC15 is positively correlated with the LEW strain and negatively correlated with DA, red circles represent activated pathways in LEW and blue indicates upregulated pathways in DA. No pathway was significantly upregulated in DA. Transcriptional factor (TF) binding site-based gene set enrichment analysis using gProfiler on the ChEA ChIP-Seq database identifies TFs which may be activated in (E) DA and (F) LEW myeloid cells. TFs are sorted based on their enrichment significance calculated as –log10(adjusted p value). Dark purple indicates higher significance. Purple boxes highlight TFs which are uniquely enriched in that strain.

Comparing the expression level of the top varimax PC15 genes in the myeloid cells of the two strains confirms the strain-specificity of this factor (Figures 5A–5C). Pathway analysis identified higher activation of lymphocyte-mediated immune responses, lymphocyte migration and chemotaxis, response to interferon, and allograft rejection pathways in LEW compared to DA *Marco*-enriched myeloid cells (Cluster 5) (Figure 5D; Table S7). This factor is enriched in myeloid and T cell differentiation transcription factors (TFs) (Figures 5E and 5F). LEW-enriched TFs include *Irf8*, *Irf1*, *Spi1*, *Pou5f1*, *Stat4*, and *Stat5a*, which are mostly inflammatory process-associated genes present in chronic diseases like rheumatoid arthritis^72^^,^^73^^,^^74^ (Figure 5F). *Irf1*, *Irf8*, *and Spi1* (PU.1) work cooperatively to shape the chromatin landscape to polarize macrophages for inflammatory responses, while *Stat4* deficiency leads to repolarization toward alternatively activated macrophages.^72^^,^^73^^,^^74^ The DA-specific TFs include *PPAR-γ*, *Nucks1*, *Runx1*, *Mitf*, and *Gata1*, which have been described more broadly in the literature^73^^,^^75^^,^^76^^,^^77^ (Figures 5E and S18; Table S6). For example, *PPARγ* is associated with M2-like macrophage polarization, *Nucks1* and *Runx1* are implicated in immunomodulation, and *Gata1* and *Mitf* are associated with cell fate and differentiation.^78^^,^^79^^,^^80^^,^^81^

No strong myeloid-specific strain-related varimax factors were discovered using the snRNA-seq map, which can be explained by the lower representation of non-inflammatory myeloid cells within the snRNA-seq map (276 cells) compared to the scRNA-seq TLH map (1,668 cells). However, we were able to validate the myeloid-specific strain factor identified in the scRNA-seq TLH map. We selected the top 10 PC15-associated genes and calculated their enrichment within the myeloid cells of the snRNA-seq map. In line with our scRNA-seq results, varimax PC15 signatures show strain differences within the myeloid cells of the snRNA-seq samples (Figures S19A–S19F). We also evaluated the DE genes between DA and LEW within the myeloid population of the snRNA-seq samples using a generalized linear mixed model considering covariates, like sample and strain.^82^ The most DE genes include the top strain-related genes identified by the varimax analysis approach (*Itgal*, *RT1-A1*, *Timp2*, *Lilrb3l*, *RT1-T24-3*) along with some expected ambient-RNA transcripts (Figure S19G). These results suggest that the baseline hepatic microenvironment in the LEW rat is more pro-inflammatory compared to the DA strain and highlight myeloid cells as potential drivers of the enriched inflammatory pathway activation in LEW rats. We then considered whether myeloid cell frequency in the DA and LEW livers may be influencing the inflammatory status of LEW rats. Alterations in cell-type frequencies in scRNA-seq data are confounded by sample-specific dissociation efficiency. Therefore, we employed immunohistochemistry to compare the frequency of CD68^+^ cells between the two strains. However, quantification of CD68 staining showed no significant difference in the frequency of CD68^+^ cells in LEW vs. DA (Figures 3G and 3H). These results suggest that the variations in inflammatory potential are not likely caused by differences in the frequency of intrahepatic CD68^+^ cells.

### Immune enrichment maps rat lymphocyte and myeloid populations at higher resolution

**Figure 6:**
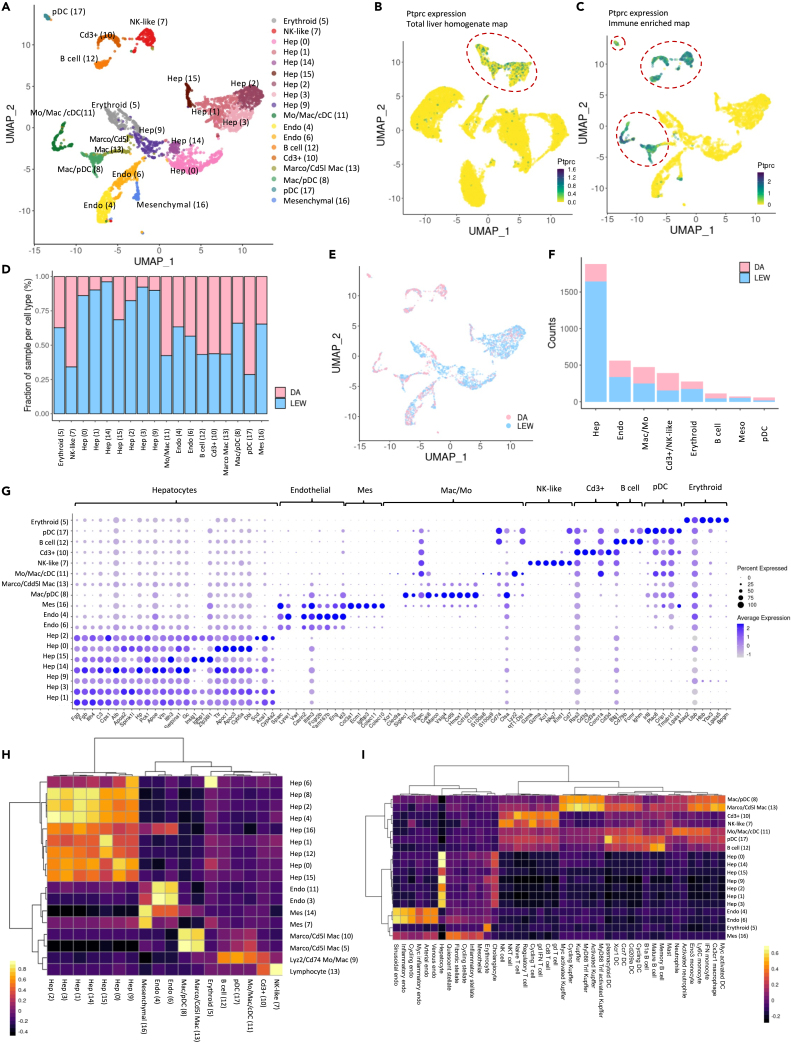
An immune-enriched scRNA-seq rat liver map provides a higher resolution of lymphocytes and myeloid populations (A) UMAP projection of immune-enriched samples where cells that share similar transcriptome profiles are grouped by colors representing unsupervised cell clustering results. As opposed to the total liver homogenate map, B cells and plasmacytoid dendritic cells (pDCs) have been well-captured in the immune-enriched map, and *Cd3*+ and NK-like cells form distinct populations. The legend indicates the unique color representing the cell-type annotation of each cluster. The cluster number is shown within the curved brackets. (B) Expression distribution of *Ptprc*, a general immune cells marker, over UMAP projection of total liver homogenate cells. (C) *Ptprc* expression over UMAP projection of immune-enriched map’s cells. Comparison with Figure 4B indicates that the immune-enriched map provides a better representation of the immune population compared to the total liver homogenate map. (D) Bar plot indicating the relative contribution of input samples to each cell population. Both samples have been represented in each of the clusters (cell types). (E) Labeling UMAP projection of cells based on the input sample indicates that cells from different samples have been well-integrated and clusters represent cell-type differences rather than sample-specific variations. (F) The number of cells in each major population colored by the contribution of each input sample. (G) Dot plot indicating the relative expression of marker genes in each population. The size of the circle indicates the percentage of cells in each population which express the marker of interest. (H) Comparison of total liver homogenate and immune-enriched rat liver maps. Rows and columns of the correlation heatmap represent the clusters within total liver homogenate and immune-enriched maps, respectively. The color of the heatmap cells indicates Pearson correlation values between the cluster average gene expressions. The top 500 highly variable genes in each map were used for correlation calculation. The dotted box indicates that the total liver homogenate map’s cluster 9 has a high correlation with B cells, pDCs, myeloid cells, and *Cd3*+ cell population of the immune-enriched map, confirming that immune-enriched map provides a higher resolution of lymphocytes and myeloid cells. Non-immune cell types of the two maps are consistent. (I) Comparison of rat healthy liver immune-enriched map and mouse healthy liver map [https://pubmed.ncbi.nlm.nih.gov/33106666/]. The rows and columns of the correlation heatmap represent the rat and mouse clusters, respectively. The color of the heatmap cells indicates Pearson correlation values between the cluster average gene expressions. The one-to-one orthologs in the top 2,000 highly variable genes of the two maps were used for correlation calculation (see STAR Methods). The comparison indicates a high consistency between the gene expression pattern of hepatic cell types between rats and mice.

Our scRNA-seq TLH map and snRNA-seq map contained hepatocyte-derived ambient RNA, as expected^43^ (Figure S20), which interfered with immune cell marker identification and resulting immune cell annotation. To provide a more detailed resource of rat hepatic immune cells, two additional immune-enriched samples were mapped (Figure 6A). These samples underwent additional washing steps and red blood cell depletion to reduce the hepatocyte-released ambient RNA (Figure S20). The percentage of cells annotated as hepatocytes decreased from 71.14% in the scRNA-seq TLH map to 49.11% in the immune-enriched map. The general immune cell marker, *Ptprc*, was expressed in 24% of the total cells in the immune-enriched map compared to 4% within the initial map (Figures 6B and 6C). Unfortunately, the scRNA-seq TLH and immune-enriched maps could not be integrated computationally, presumably due to the technical differences in their generation (Figures S21A–S21C). The varimax-based pipeline was also ineffective to deconvolute the sources of variation in the merged dataset of both sets of samples (Figure S21D). Consequently, the immune-enriched samples were analyzed separately. In total, 3,830 (1,161 + 2,669) single cells from the DA and LEW samples were integrated into the immune-enriched map after quality control (see STAR Methods; Figure S22). Similar to the TLH map, the immune-enriched samples were batch-corrected and the final clusters represent cells from both DA and LEW rats (Figures 6D and 6E). The clusters were annotated based on the same approaches used for the initial samples (Extended results; Table S8).

The immune-enriched map has captured a more diverse set of liver-resident immune cells (Figures 6A and 6F), enabling a more detailed description of these cell populations (Figure 6G) compared to the scRNA-seq TLH and snRNA-seq maps. A comparison of the scRNA-seq TLH and immune-enriched maps using correlation analysis confirmed that the immune-enriched map provides a higher resolution of lymphocytes and myeloid cells (Figure 6H). As a refinement to the immune annotations in the TLH map, individual populations of *Cd3*^+^ T cells (clusters 10), natural killer (NK)-like cells (cluster 7), B cells (cluster 12), and pDCs (cluster 17) were identified (described in the following) in the immune-enriched map (Figures 6G and 6H). Cluster 10 was characterized by enriched expression of *Cd3*^+^ T cell markers (*Cd3g*, *Cd3e*, *Cd3d*, *Coro1a*) (Figure S23). Cluster 12 identified a subset of cells enriched for B cell genes *Cd19*, *Ms4a1 (Cd20)*, *Ighm*, *Cd74*, *Cd79b*, and *Fcmr*, with no expression of *Ighd* or *Ighg*, suggesting that this cluster might be *Cd19*^+^*Cd20*^+^*IgM*^*+*^*IgD*^*-*^ immature B cells^83^ (Figure S24). The correlation heatmap (Figure 6I) indicated high gene expression similarity with the mouse^84^ B cell populations, supporting that this is a B cell population. Enriched gene expression in cluster 17 correlates with both monocyte-like macrophages (*Cd74* and *Tyrobp*), and pDCs (*Siglech*,^34^ *Ptprcap*,^85^ and *Ptcra*^85^) (Figure S25). When comparing the expression of this cluster to the mouse liver cell atlas, we see a high correlation with pDCs (Figure 6I), suggesting that the predominant cellular population of this cluster may be pDC enriched.^84^ Cluster 11 displays a correlation with monocytic macrophages and dendritic cells and similar DE genes as scCluster 9 suggesting it is a mixture of recently recruited immune populations (Figure S26). DE genes in cluster 7 include *Tbx21* [aka T-bet], *Ncr1*, *Prf1*, *Nkg*7, *Ccl5*, *Cd8a*, *Gzmk*, *Klrd1*, and *Cd7*, with low expression o*f Cd3d*, suggesting it is an NK-like population^7^^,^^26^^,^^27^^,^^28^^,^^29^^,^^30^^,^^86^ (Figure S27). The expression of top genes in this cluster correlated with the NK cell population in the mouse dataset (Figure 6I) reinforcing that this cluster is an NK-enriched cluster. The *Ptprc*^+^ clusters of the immune-enriched map were subclustered for further evaluation (Figures S28–S33; Table S9). Upon subclustering of the *Ptprc*^+^ clusters, cDCs (cDC1: *Clec9a*, *Xcr1*, *Batf3*; cDC2: *Clec10a*, *Tmem176b*^87^^,^^88^^,^^89^), which were mixed with other immune populations in the TLH, formed a separate subcluster indicating a higher resolution result (Figures S25, S26, and S33). Analysis of subcluster 5 (77 cells) (Figure S33) revealed enriched expression of recently recruited monocyte/macrophage markers Cst3 and Cd74, as well as cross-presenting DC markers Xcr1, Clec9a, and Tlr3.^90^ When looking at expression of these DC markers in our uniform manifold approximation and projections (UMAPs), we see that a subpopulation on the right side of this subcluster had enriched expression of cDC1 genes (Xcr1, Clec9a^34^) and the subpopulation on the left is enriched in cDC2 markers (Clec10a,^34^ Tmem176b^87^^,^^89^), suggesting that this subcluster may contain a mixture of cDC1- and cDC2-like cells.

Comparison of previously published mouse liver data with the rat single-cell atlas indicates high consistency of the majority of the cell types between these two species (Figure 6I). We also attempted to determine if we could capture the strain-specific factors identified based on the TLH scRNA-seq map (Figure S34) in the scRNA-seq immune-enriched map. Similar to the snRNA-seq validation, we selected the top 10 genes which represented each factor and evaluated their enrichment pattern within the immune-enriched map. In line with our previous predictions, both varimax PC5 and 15 signatures show strain differences within the immune-enriched samples and are specific to hepatocyte and myeloid populations, respectively. Using immunohistochemistry, we then examined if the presence of infiltrating T cells (CD3, CD8) correlates with the differences in inflammatory potential. A periportal-biased presence of T cells was detected, but no significant frequency differences between strains were observed (Figure S12). In summary, the immune-enriched map represents a more detailed evaluation of the immune landscape of the healthy rat liver and provides additional information on B cells, DCs, *Cd3*^+^ T cells, and NK-like populations in comparison to the TLH scRNA-seq map.

### Validation of computationally inferred strain-specific inflammatory differences

**Figure 7:**
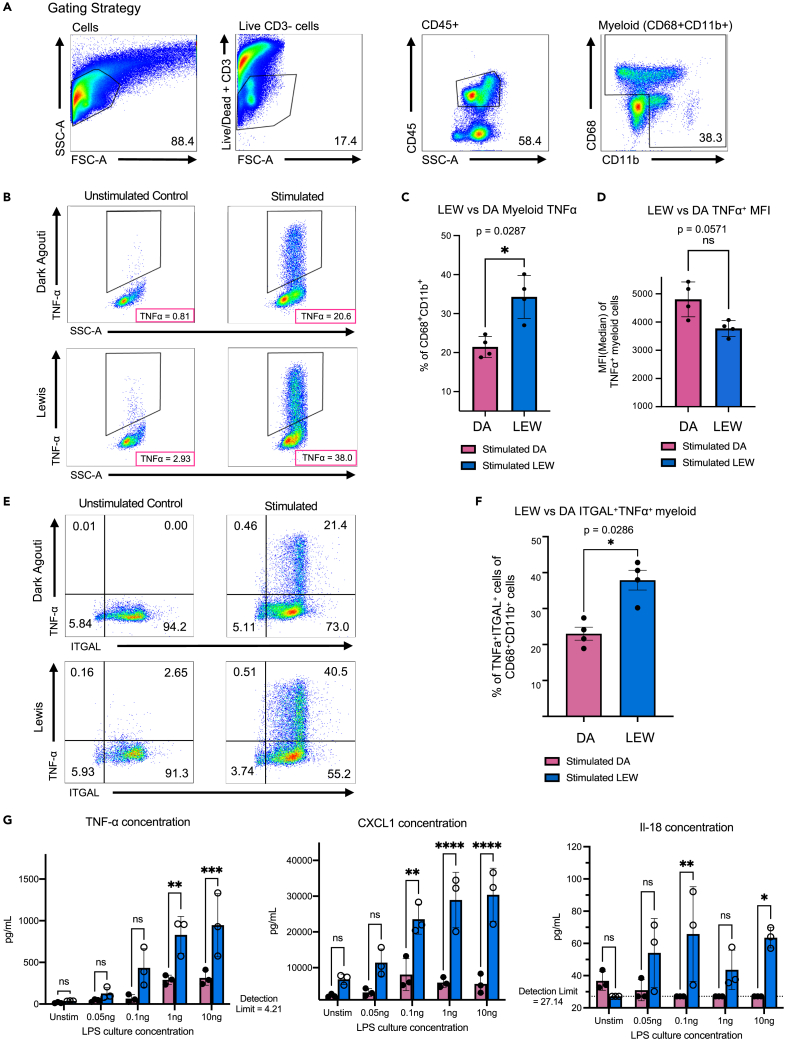
The inflammatory potential of myeloid cells found in LEW rats is greater than that found in DA rats Myeloid cell inflammatory potential was evaluated after lipopolysaccharide (LPS) stimulation of freshly isolated liver-resident non-parenchymal cells. LPS-induced TNFα secretion was measured via intracellular cytokine staining (ICS). The non-parenchymal liver cell dissociate was obtained via a gentle enzymatic perfusion process and differential centrifugation. The resulting cells were plated in 12 well plates for 3.5 h before being stimulated for 6 h under a concentration of 1 ng/mL of LPS in the presence of 1:1000 concentration of Monensin and Brefeldin. (A) Flow cytometry plots showing the gating strategy for macrophages. (B) Percentage of TNFα^+^ secreting CD68^+^CD11b^+^ myeloid cells in the unstimulated control and stimulated conditions of Dark Agouti and Lewis macrophages. (C) Summary graphs of Lewis versus Dark Agouti total TNFα as a percentage of CD68^+^CD11b^+^myeloid, (D) and of the mean fluorescence intensity (MFI) of Lewis vs. Dark Agouti TNFα. (E) Representative flow cytometry plot of TNFα secretion patterns based on ITGAL subpopulations. (F) and summary graph ITGAL expressing CD68^+^CD11b^+^ myeloid subpopulations. Plotted are the values from all 4 experimental replicates. Statistical significance for ICS was determined using a non-parametric 2 tailed Mann-Whitney test. (n = 4) (G) Cytometric bead array (LEGENDplex) was performed to quantify the level of cytokines (TNFα, Il-18, CXCL1) on culture supernatants of enriched CD68^+^ myeloid cells after 24 h of stimulation in various LPS concentration conditions (0, 0.05, 0.1, 1, 10 ng/mL). Three technical replicates were used per animal. Statistical significance of the CBA was determined using a two-way ANOVA and Sidak’s multiple comparisons test (n = 3) Data are represented as mean ± SEM with each dot representing a single animal. (∗: p value <0.05, ∗∗: p value <0.01, ∗∗∗: p value <0.001,∗∗∗∗: p value <0.0001) DA: dark agouti, LEW: lewis, SSC-A: side scatter area, FSC-A: forward scatter area.

To functionally validate the computationally identified strain-specific differences in the inflammatory potential of hepatic myeloid cells, we performed *ex vivo* LPS stimulations followed by intracellular cytokine staining. In these assays, we LPS-stimulated fresh non-parenchymal cells separated by differential centrifugation from flushed, enzymatically dissociated LEW and DA rat livers. We examined cytokine secretion from tissue-resident myeloid cells via intracellular cytokine staining for tumor necrosis factor alpha (TNFα) (see STAR Methods; Figure S35). We found a higher frequency of LEW intrahepatic myeloid cells (CD45^+^CD68^+^CD11b^+^) secreting TNFα in response to LPS stimulation compared to DA liver-resident myeloid cells (Figures 7A–7C), which suggests, in agreement with the computational findings (Figures 5C–5F), that the inflammatory potential of the hepatic myeloid cells in LEW rats (% TNFα positive = 35.25 ± 3.18 (SEM)) is higher than that of DA rats (%TNFα positive = 22.25 ± 1.45 (SEM)). However, despite the overall higher per-cell TNFα response in LEW myeloid cells, the overall difference in the TNFα^+^ mean fluorescence intensity (MFI) did not reach significance (Figure 7D). In the computational analysis, the higher inflammatory potential of LEW liver myeloid cells was accompanied by the relative enriched expression of *Itgal* transcripts (Figure 4H), which corresponds to the protein Integrin Subunit Alpha L (ITGAL). ITGAL is a component of Lymphocyte function-associated antigen 1 (LFA-1), the expression of which is associated with inflammation and several autoimmune conditions.^91^ Further examination of the post-stimulation intracellular cytokine data revealed that the strain-specific pro-inflammatory differences rested primarily within ITGAL^+^ myeloid cells, reflecting bioinformatic analysis that the LEW liver possesses a more inflammatory CD68^+^ CD11b^+^ myeloid population (Figures 5, 7E, and 7F). We also observe a lack of strain-specific differences in the frequency of either CD68^+^ITGAL^+^ or CD68^+^ myeloid cells in the flow cytometry analysis (Figure S36). This finding is consistent with previous studies showing that DA liver myeloid cells exhibit less inflammatory characteristics, and a muted ability to stimulate T cell proliferation in comparison to LEW myeloid cells in mixed lymphocyte alloreaction assays.^92^ To expand on the characterization of myeloid function in these strains, CD68^+^ magnetic bead-based myeloid cell purification was performed on three pairs of LEW and DA rat liver TLH cell suspensions (Figures S37 and S38). The pro-inflammatory cytokine production of these cells in response to a series of LPS concentrations was then measured via a multiplexed rat cytometric bead array (CBA). Although hepatic myeloid cells from both strains displayed a dose-dependent cytokine response to LPS stimulation (Figure S39), LEW myeloid cells secreted significantly more interleukin-18 (IL-18), a LEW-enriched gene in varimax PC15 (Figure 5B), compared to DA myeloid cells (Figure 7G). Moreover, inflammatory cytokines (IL-6, IL-1α, GM-CSF, CXCL1) that are regulated by TFs positively enriched in varimax PC15, such as *PU.1*,^93^^,^^94^ *Irf8*, *Irf1*,^95^ *C/EBP-β*,^96^^,^^97^ and *Stat4*^98^^,^^99^ (Figures 5E and 5F), are also elevated in the stimulated LEW versus DA myeloid cells (Figures 7G and S39). Examination of these strain-specific inflammatory potential differences may serve as a point of focus for further investigation of the mechanisms behind immune-regulated hepatic disease susceptibility such as hepatic neoplasia, and liver transplant rejection.

## Discussion

In this study, we used a multi-platform approach to create a multi-strain atlas of the healthy rat liver. This resource helps identify rat hepatic cell types and serves as a useful baseline for hypothesis generation or to identify cellular alterations in liver disease models. We identified key immune and parenchymal populations in the healthy rat liver and their marker genes and examined their zonation tendencies within hepatic lobules. We also identified *in silico* strain-specific differences in hepatic myeloid populations isolated from DA and LEW rats, findings which we validate using *ex vivo* assays. This study illustrates cellular and molecular sources that may contribute to strain differences and highlights the potential role of myeloid cells in contributing to the baseline inflammatory state in the LEW model liver compared to the DA liver.

Tissue dissociation is a major challenge in single-cell studies of the liver, as different cell types respond differently to dissociation protocol conditions.^43^ We mitigated this challenge by using a combination of multiple dissociation conditions, multiple single-cell mapping technologies (scRNA-seq and snRNA-seq), and spatial transcriptomics to capture populations not well represented by either technology individually. This combined approach enabled us to better capture the diverse set of liver cell types and their zonation signatures.

Matrix factorization methods, such as varimax-rotated and standard PCA, enabled us to identify cellular identity and strain-related differences within our scRNA-seq dataset, in addition to identifying lobule zonation signatures within the spatial transcriptomics data. We found that myeloid cells from LEW livers have higher inflammatory potential than those from DA livers. We demonstrated this at the transcriptional level via scRNA-seq and confirmed this with snRNA-seq, an approach which is more resistant to dissociation-induced biases. These findings were functionally verified *in vitro* through intracellular cytokine staining and via the measurement of secreted cytokines following LPS stimulation of DA and LEW myeloid cells. We speculate that there is a baseline higher inflammatory milieu in the LEW rats that drives the strain-specific differences in these animals.

To examine the relevance of these identified strain differences, future rat liver atlasing efforts should include disease states such as fibrosis, ischemia reperfusion injury, or transplant rejection. Longitudinal atlasing of the liver microenvironment in these scenarios will provide valuable insights into disease-promoting populations, potentially leading to new targets to limit hepatic inflammation. Our data support the notion that reprogramming hepatic myeloid cells may be an attractive avenue to target and modulate inflammation in the rat liver.^100^ Taken together, our transcriptomic maps of the rat liver microenvironment contribute to our understanding of the cellular basis of the rat liver function in addition to uncovering hepatic differences between rat strains. They also provide a framework to investigate new therapeutic options in this model animal, which can be ultimately transferred to humans to cure and prevent hepatic inflammation.

### Limitations of the study

We recognize several limitations in our study. First, increasing the scRNA-seq datasets’ sample size could provide higher statistical power. We opted to use an independent snRNA-seq dataset to increase the robustness of our findings. Compared to the former, snRNA-seq is less prone to dissociation-sensitive bias and can better capture sensitive parenchymal populations such as cholangiocytes. Rat studies are generally limited by a lack of immunological tools available, which limits the scope of *in vitro* validation strategies. This issue can be improved upon by testing and optimizing tools from other model systems for cross-reactivity and producing rat-specific antibodies. As well, ambient RNA is a major technical issue for studying liver tissues using sc/snRNA-seq technologies and can mask liver biological signals. This background noise was prominent in rats compared to humans, possibly due to the smaller vasculature, leading to more challenging tissue dissociation. As a result, we relied on factorization approaches, such as varimax PCA, to identify and separate biological and technical signals. Current computational methods for ambient RNA removal are limited^101^ and were unable to remove the technical contamination while preserving the biological signal. Improvements in ambient RNA removal methods in the future will be beneficial to liver single-cell studies. We annotate the key DE genes expressed in each cluster and acknowledge that contamination by additional populations cannot always be excluded. Refinement of cell population and strain variation annotation, including rare cell populations, is of interest for future studies. Single-cell-resolution spatial transcriptomics methods will be useful for this. The integration of single-cell sequencing assay for transposase-accessible chromatin (scATAC-seq) and cellular indexing of transcriptomes and epitopes by sequencing (CITE-seq), which can capture epigenomics, transcriptomics, and protein expression, will also lead to more refined annotations of rare cell populations. Our map only includes male samples. The inclusion of female samples will be important to understand sex-related differences in the liver.

## STAR★Methods

### Key resources table

**Table:** 

REAGENT or RESOURCE	SOURCE	IDENTIFIER
**Antibodies**		

Zombie Aqua™ Fixable Viability Kit	BioLegend	Cat# 423101
Mouse anti-rat CD45 BV786; Clone: OX-1	BD Biosciences	Cat#740914; RRID: AB_2740556
Mouse anti-rat CD11b V450; Clone: WT.5	BD Biosciences	Cat# 562108; RRID: AB_10898164
Mouse anti-rat CD3 PE; Clone: 1F4	BD Biosciences	Cat# 550353; RRID: AB_393632
Mouse anti-rat CD3 BV510; Clone: 1F4	BD Biosciences	Cat# 624289
Mouse anti-rat CD68 AF700; Clone: ED1	Novus Biologicals	Cat# NB600-985AF700
Mouse anti-rat CD11a PE; Clone: ED1	BD Biosciences	Cat# 550972; RRID: AB_393985
Recombinant anti-mouse/rat TNFα; Clone: EPR21753-109	AbCam	Cat# ab283321
Mouse anti-rat CD32; Clone: D34-485	BD Biosciences	Cat# 562189; RRID: AB_11153308
Recombinant anti-rat CD68; Clone: REA237	Miltenyi Biotec	Cat# 130-102-723; RRID: AB_2659012
LEGENDplex™ Rat Inflammation Panel Detection Antibodies V02	Biolegend	Cat# 740266; RRID: SCR_001134
Rabbit anti-CD3; Clone: 2GV6	Roche Diagnositics	Cat# 05278422001; RRID: AB_2335978
Mouse anti-rat CD8α; Clone: OX-8	Bio-Rad	Cat# MCA48G; RRID: AB_321476
Mouse anti-Bovine/Dog/Human/Mouse/Rat Hmox1; Clone: HO-1-1	Thermo Fisher Scientific	Cat# MA1-112; RRID: AB_2536823
Rabbit anti-Mouse/Rat CD68; Clone: Polyclonal	AbCam	Cat# ab125212; RRID: AB_10975465
Mouse-anti rat CD163; Clone: ED2	BioRad	Cat# MCA342GA

**Chemicals, peptides, and recombinant proteins**		

Heparin	LEO	Cat# 006174-0
20G cannula	Braun	Cat# 4252535-02
Hank’s Balanced Salt Solution	Gibco	Cat# 14170161
EGTA	Bioshop	Cat# EGT101
DMEM with HEPES	Gibco	Cat#21063045
Fetal bovine serum	Cytiva	Cat# SH3039603
Penicillin-streptomycin	Sigma-Aldrich	Cat# P4333
Lipopolysaccharide; Lot# 028M4094V	Sigma-Aldrich	Cat# L2880-250MG
Golgiplug (Brefeldin)	Biolegend	Cat# 555029
Golgistop (Monensin)	Biolegend	Cat# 554724
Collagenase	Sigma-Aldrich	Cat# C5138
CaCl2	Sigma-Aldrich	Cat# 21115
Tris-HCl pH 7.5	Thermo Fisher Scientific	Cat# 15567027
NaCl	Sigma-Aldrich	Cat# 59222C
MgCl_2_	Sigma-Aldrich	Cat# M1028
Ultrapure RNase/DNase free distilled water	Thermo Fisher Scientific	Cat# 10977023
SYBR Green	Thermo Fisher Scientific	Cat#S7564
CHAPS hydrate	Sigma-Aldrich	Cat# C3203
Bovine Serum Albumin	New England BioLabs	Cat# B9000S
HEPES	Gibco	Cat# 15630080

**Critical commercial assays**		

Transcription Factor and Staining Buffer Set	eBioscience	Cat# 00-5523-00
Chromium Single Cell Reagents 3′ Library & Gel Bead Kit v2, 16 rxns	10x Genomics	Cat# PN-120237
Cytofix/Cytoperm	BD Biosciences	Cat# 20554714
Single Cell Reagents 3ʹ GEM, Library & Gel Bead Kit v3, 16 rxns	10x Genomics	Cat# PN-1000075
Chromium Next GeM Chip A	10x Genomics	Cat# PN-120236
EasySep™ FITC Positive Selection Kit II	Stem Cell Technologies	Cat# 17682
Visium Spatial Tissue Optimization Slide Kit v1	10x Genomics	Cat# PN-1000191
Visium Spatial Tissue Optimization Reagent Kit v1	10x Genomics	Cat# PN-1000192
Library Construction Kit	10x Genomics	Ca# PN-1000190
Visium Spatial Gene Expression Slide Kit	10x Genomics	Cat# PN-1000185
Dual Index Kit TT Set A	10x Genomics	Cat# PN-1000215
Chromium Next GEM Chip G B	10x Genomics	Cat# PN-1000073

**Deposited data**		

Raw and analyzed data	This Paper	GEO: GSE220075
Interactive sc/snRNA-seq and spatial maps	This Paper	https://rat-liver-atlas.cells.ucsc.edu

**Experimental models: Organisms/strains**		

Rat LEW/SsNHsd	Envigo	Cat#017
Rat DA/OlaHsd	Envigo	Cat#092
Rat Wistar	Envigo	Cat#012

**Software and algorithms**		

GraphPad Prism 9	GraphPad Software	https://www.graphpad.com/
FlowJo 10	Treestar	https://www.flowjo.com/solutions/flowjo
Qognit™ LegendPlex™ Software	Biolegend	https://legendplex.qognit.com/
Qupath 0.2.3	Qupath	https://qupath.github.io/
10x Genomics CellRanger v3.1.0	Zheng et al.^102^	https://www.10xgenomics.com/
10x Genomics SpaceRanger v1.1.0	N/A	https://www.10xgenomics.com/
R v4.0.3	N/A	https://www.R-project.org/
Seurat v4.0.2	Hao et al.^103^	https://github.com/cran/Seurat
Harmony v1.0	Korsunsky et al.^40^	https://github.com/immunogenomics/harmony
scDblFinder v1.10.0	Germain et al.^104^	https://github.com/plger/scDblFinder
ggplot2	Wickham.^105^	https://github.com/tidyverse/ggplot2
scClustViz	Innes and Bader.^106^	https://github.com/BaderLab/scClustViz
NEBULA v1.3.0	He et al.^82^	https://github.com/lhe17/nebula
randomForest v4.6.14	Breiman.^107^	https://github.com/cran/randomForest/
caTools v1.18.2	N/A	https://CRAN.R-project.org/package=caTools
UCell v1.0.0	Andreatta and Carmona.^108^	https://github.com/carmonalab/UCell
Gene Set Enrichment Analysis (GSEA)	Subramanian et al.^109^	software. broadinstitute.org/GSEA
gProfile	Reimand et al.^110^	https://biit.cs.ut.ee/gprofiler/gost
Cytoscape v3.8.2	Shannon et al.^111^	https://cytoscape.org/
EnrichmentMap v3.3.2	Merico et al.^112^^,^^113^	https://apps.cytoscape.org/apps/enrichmentmap
AutoAnnotate v1.3.4	Kucera et al.^112^^,^^113^	https://apps.cytoscape.org/apps/autoannotate
biomaRt v2.46.2	Durinck et al.^114^	https://github.com/grimbough/biomaRt
Analysis code	This Paper	https://github.com/BaderLab/HealthyRatLiverMap

**Other**		

LSR Fortessa	BD Biosciences	N/A
NovaSeq 6000	Illumina	N/A
HiSeq 2500	Illumina	N/A
EasyEights™ EasySep™ Magnet	Stem Cell	Cat# 18103
Epredia™ CryoStar™ NX70 Cryostat	Fisher Scientific	Cat#14-071-407
Leica Aperio AT2 whole slide scanner	Leica Microsystems	N/A
Rat/Mouse Chow	Harland-Teklad	Cat#LM-485 7912.15

### Resource availability

#### Lead contact

Further information and requests for resources and reagents should be directed to and will be fulfilled by the lead contact, Sonya MacParland (sonya.macparland@uhnresearch.ca).

#### Materials availability

This study did not generate new unique reagents.

### Experimental model and study participant details

#### Experimental animals

Healthy male Dark Agouti (DA), Lewis (LEW) rats were purchased from Envigo and bred under the Animal Research Center at Krembil Research Institute in a specific pathogen free facility. Rats were maintained under 12 hour light-dark cycles with free access to chow (Harlan-Teklad) and water. 16–18 week old LEW (300-350g) and DA (260-300g) rats were age matched to be selected for experiments. LEW rats weighed more than DA rats across all ages. All experimental procedures followed principles and guidelines for the care and use of animals established by the Animal Resources Centre (ARC) at the University Health Network and are in accordance with the guidelines of the Canadian Council of Animal Care. Rat experiments were performed at the Toronto General Research Institute, Toronto, ON, Canada under the approval of the Institutional Committee on Animal Bioethics and Care (AUP 5840). All surgery was performed under isofluorane anesthesia, and all efforts were made to minimize suffering.

### Method details

#### Rat liver tissue collection for snRNA-seq and fresh single cell suspension preparation

All rats were anesthetized with 5% isoflurane with an anesthetic apparatus, and the abdominal cavity was opened. Heparin (LEO Pharma) is directly injected into the Inferior Vena Cava (IVC). The median lobe (ML) was tied and the left side of the ML was collected for snap freezing in liquid nitrogen for downstream snRNA-seq applications. The IVC is then cannulated with a 20G cannula (Braun) and flushed with a 4°C HBSS Ca2^+^Mg2^+^ Free solution (Gibco) solution with 0.01mM EGTA (Bioshop) at a rate of 10 mL/min for 5 minutes, followed by a warm 37°C HBSS Ca2^+^Mg2^+^Free solution (Gibco) with 0.01M of HEPES(Gibco), 1.35 mM of CaCl_2_ (Sigma-Aldrich) and 0.04% Collagenase (Sigma-Aldrich) at a rate of 5 mL/min for 12 minutes. The digested liver is excised into a 4°C HBSS solution, and the Glisson capsule is shaken and opened to release the resulting single cell suspension. This total liver homogenate is filtered by a 70 um mesh filter (Falcon) before scRNAseq submission. Further processing for the immune-enriched samples involves the removal of parenchymal cells via differential centrifugation at 60xG for 10 minutes. The supernatant is then washed and resuspended as NPCs for scRNA-seq submission. Additionally, NPCs prepared in this method were also used for cell culture.

#### Nuclei preparation for snRNA-seq

4-mm^2^ liver pieces were snap frozen in liquid nitrogen. The pieces were retrieved for nuclei extraction within 1 year of snap freezing. Nuclei processing was performed according to the CHAPS with salts and Tris (CST) protocol published by the Broad Institute.^116^ A 2X stock of salt-Tris solution (ST buffer) composed of pH 7.5 20 mM Tris-HCl (Thermo Fisher Scientific), 292 mM NaCl (Sigma-Aldrich), 2 mM CaCl2 (Sigma-Aldrich) and 42 mM MgCl2 (Sigma-Aldrich) in Ultrapure water (Thermofisher) was made. 1X ST buffer was prepared via an additional 1:1 dilution with Ultrapure water. CST detergent buffer was prepared with 1ml 2X ST stock solution, 0.0049% CHAPS (Sigma-Aldrich), and 0.0001% BSA (New England BioLabs). Each liver piece was chopped using spring scissors for 10 minutes in the CST buffer. The solution was washed with 1X ST, poured through a 40uM mesh filter (Falcon), spun down at 500g for 5 minutes and filtered again for 10x Genomics snRNA-seq submission.

#### Visium spatial transcriptomics slide processing

Healthy Wistar rat liver tissue was embedded in optimal cutting temperature (OCT), frozen and stored at −80°C. The frozen tissues were cryosectioned at a 16-um thickness at −14°C (Cryostar NX70 HOMP) and placed on a chilled Visium Tissue Optimization Slide (10x Genomics). A 9 minute tissue permeabilization was performed and samples were prepared according to the manufacturer’s guidelines.

#### 10x sample processing and cDNA library preparation

Samples were prepared as outlined by the 10x Genomics Single Cell 3′ v2 (scRNA-seq TLH samples) and v3 (immune-enriched samples and snRNA-seq samples) Reagent Kit user guidelines.^7^^,^^43^ Briefly, following cell counting (using Trypan blue exclusion for single cell and SYBR green II for single nuclei), we targeted the capture of 9000 cells and loaded them onto the 10x Genomics Single cell A Chips for the total liver homogenate, and B Chips for the immune-enriched and snRNA-seq samples. Visium spatial transcriptomics were sequenced at 60,000 reads per spot with 2400 and 2500 number of spots for sample A1 and B1, respectively. cDNA libraries were prepared as per the Single Cell 3′ Reagent Kits v3 user guide for scRNA-seq and snRNA-seq and, the Visium Spatial Gene Expression Reagent Kits user guide was used for spatial transcriptomics. scRNA-seq TLH were sequenced on a HiSeq 2500. Visium spatial transcriptomics, snRNA-seq and immune-enriched samples were sequenced on a NovaSeq 6000. Sequencing QC summaries for each liver profile are found in Table S1.

#### Visium spatial transcriptomics analysis

The two Visium spatial transcriptomic data (A, B) of the healthy Wistar rat liver were sequenced to a depth of 211,345,626 and 249,614,915 reads, a saturation of 72.2% and 71.4% respectively. These reads were mapped to the reference genome Rattus_norvegicus.custom_6.0.98 and expression was quantified with the spaceranger-1.1.0. Further processing and visualization were performed with Seurat. Each sample was separately processed and genes with detection frequency of less than 0.05 and maximum read capture of less than 3 were removed during the quality control. Samples were normalized using the SCTransform function and PCA was applied to each data to identify the principal components that represent zonation patterns.

#### Quality control, normalization, and map integration

All the fastq files were run on 10 Genomics cell ranger 3.1.0 pipeline with reference genome Rattus_norvegicus.custom_6.0.98. The CellRanger (10x Genomics) analysis pipeline was then used to construct the gene expression matrix from all rat samples. The resulting raw gene expression matrix was filtered based on established quality control criteria (library size, mitochondrial transcript ratio, and the number of expressed genes per cell) using R (version 4.0.3) [https://www.R-project.org/]. Parameters for all quality control criteria were optimized for each sample using a parameter scan and parameter effectiveness was evaluated by manual inspection of the quality of the resulting clustering, visualization, and cell-type annotation, as established.^41^ Parameters were optimized separately for each sample of the total liver homogenate and immune-enriched maps, as each had different quality levels (Figure S1). Various parameters were tested for each sample to maximize low-quality cell (indicated based on library size and mitochondrial gene transcript ratio) removal while minimizing the loss of viable cells. Cell filtering was performed as follows: cells with low (total liver homogenate: [DA-1, LEW-1, LEW-2 <1500; DA-2 <2000], immune-enriched: <1000, snRNA-seq map: <1000) library size and high (total liver homogenate: [DA-1>30; DA-2>20; LEW-1>40; LEW-2>40], immune-enriched: >50, snRNA-seq: >10) mitochondrial gene transcript ratio were removed. The distribution of quality control covariates over the three maps indicates that no cluster is highly enriched in these covariates (Figures S1, S2, and S22). As expected, hepatocyte clusters have slightly higher mitochondrial gene expression. We also evaluated three different mitochondrial fraction cut-offs for the total homogenate map to ensure that our map was robust at all mitochondrial cut-offs (Table S2). Because of additional washing steps and removal of ambient RNA applied to immune-enriched samples, the immune-enriched map had a higher baseline quality, therefore, less stringent QC parameters needed to be applied. The final version of the scRNA-seq total liver homogenate, immune-enriched and snRNA-seq maps includes 23036 (cells per sample: 6623; 7112; 5457; 3844), 3830 (cells per sample: 1161; 2669) and 12497 (cells per sample: 2200; 2552; 5252; 2493) cells respectively. The median expressed genes per cell ranged from 768 to 974 for the total liver homogenate, from 662 to 1020 for the snRNA-seq map and the immune-enriched map’s values were 1138 and 1228.

Normalization and clustering of the data were performed using the Seurat (version 4.0.2)^103^ software. Each input sample was normalized using Seurat’s default ‘scTransform’^117^ normalization method, which implements a regularized negative binomial regression model for each gene. Samples were then concatenated (merged) to construct the total liver homogenate (n = 4 samples), snRNA-seq (n = 4 samples), immune-enriched (n = 2) maps. After scaling the merged gene expression matrices, principal component analysis (PCA)^44^ was used to reduce the number of dimensions representing each cell. A scree plot was used to determine the number of principal components to use for our data set, based on selecting an elbow, as established. 15 principal components were used. Harmony (version 1.0)^40^ integration was then applied to the principal components of each map to remove the technical batch variations. Non-linear dimension reduction methods, and Uniform Approximation and Projection method (UMAP)^118^ were applied to Harmony-adjusted top components for visualization.

Doublet detection was performed using the scDblFinder (1.10.0) package.^104^ Predicted doublets had a uniform distribution within the maps and were not removed (Figure S1H).

#### Cell clustering, differential expression, cluster annotation

Seurat’s shared nearest neighbor Louvain clustering algorithm was used to cluster the cells, based on the Harmony-corrected principal components. Differentially expressed (DE) genes associated with each cluster were identified using Seurat’s FindMarkers (logfc.threshold = 0, min.pct = 0, min.pct = 0, min.cells.group = 1) implementation of the non-parametric Wilcoxon rank-sum test. scClustViz^106^ was incorporated into the clustering pipeline to help find the optimal clustering resolution manually, based on known cell annotations.^41^ Resolution 2.5 was chosen for the single nuclei map, and resolution 0.6 was chosen for both the single cell total liver homogenate and immune-enriched maps. The *Ptprc*^+^ clusters of the immune-enriched map were subclustered to examine cell subtypes, and in this case resolution, 1.0 was used. Resolutions 0.4 and 1.0 were used for subclustering of the mesenchymal population of the TLH scRNA-seq and snRNA-seq maps, respectively. Endothelial populations of the snRNA-seq map were subclustered using resolution 0.8. Manual cell annotation involved evaluating the top DE genes based on known markers according to the literature. Differential expression between the DA and LEW strains within the myeloid population of the snRNA-seq map was performed using a generalized linear mixed model implemented in the NEBULA (version 1.3.0) package.^82^ Strain and sample were modeled as fixed and random effects, respectively and library size was used as an offset. Top strain-related genes were then identified by scoring the output based on the -log10(p values)∗log (Fold change) score (Figure S19).

#### Matrix factorization using varimax PCA

We used matrix factorization to separate out and study the hidden patterns (factors) within our scRNA-seq data,^119^ which may represent factors such as cell type gene expression program or a technical factor. Matrix factorization decomposes the gene expression matrix into the product of two lower-dimension matrices: 1) the loading matrix, which defines the relationship between the genes and the factors and can be used for pathway analysis and gene expression marker discovery; and 2) the score matrix, which represents the relationship between the factors and the cells and can be used for cluster analysis and dataset visualization. Here, we used a matrix factorization method called varimax PCA^68^ to identify the hidden factors within our healthy single cell RNA-seq rat liver maps, as it worked better than standard PCA. Standard PCA identifies orthogonal dimensions that capture the maximum amounts of variation in the data. Varimax PCA applies an orthogonality-maintaining rotation to the PCA loading matrix with the goal of improving the interpretability of the PCs. This higher interpretability is mathematically achieved by maximizing the variance of the squared loadings in each factor.^68^

Varimax PCA was applied to the normalized total liver homogenate, immune-enriched and snRNA-seq gene expression matrices separately. Interpretation of the varimax factors starts with matching factors with cell clusters and known covariates of interest (e.g., strain, sex). Varimax factors were serially plotted against PC1, to create a two-dimensional plot to help visually identify whether a separation on the basis of a specific cluster or strain was evident. For instance, different distributions of DA and LEW-derived cells over the factor of interest indicate that it has captured strain-specific variations (Figures 4C and 4F). Other factors visually correlated with known cell types (Figures S13 and 4B).

We used correlation analysis and random forest (RF) binary classifiers to automate the factor interpretation process. The correlation between the average gene expression of each cell cluster and the loading scores of each varimax factor was calculated. The top 10 differentially expressed genes of each cell population (cluster) were used to calculate the Pearson correlation scores. The results were plotted as a heatmap (Figure 4B), which was used to match each cluster with one or more varimax factors with a high absolute correlation value. The resulting matched factor and cluster pairs were robust to the number of selected top DE genes (10, 20, 30, 50). A Random Forest model was used to identify the varimax factors that capture strain-specific variations. This classifier was trained to predict the strain attributes of each cell by using varimax factors as input features. Evaluating the feature importance of the trained model uncovers the most informative varimax factor to predict the strain of interest. The model was implemented by the randomForest^107^ (version 4.6.14) package and evaluated using the caTools (version 1.18.2) [https://CRAN.R-project.org/package=caTools] library (Accuracy: 0.9995, Sensitivity: 0.9994, Specificity: 0.9996). The feature matrix (varimax factors) and the corresponding labels were split in a 3:1 ratio using the ‘sample.split’ function of the caTools package into the train and test sets, and the feature importance of the trained models was assessed. The factor with the highest feature importance score (as measured by the mean decrease in Gini score^120^) was chosen as the best-matched factor for the predicted covariate.

To deconvolve strain-associated biological variations from sample-related confounding factors, at least two samples per strain are required. Consequently, the immune-enriched map’s strain-specific varimax factors were disregarded. Two strain-specific factors were identified from the total liver homogenate map. To assess whether the varimax PCs represent technical or biological signals, the correlation between each factor and three major technical covariates, including library size, number of expressed genes, and percentage of mitochondrial gene expression was calculated. All the strain-specific components indicated a near-zero correlation with these technical covariates (Figure S14C). The hepatocyte-specific strain variation captured by varimax PC5 was separately discovered in the snRNA-seq atlas (varimax PC16) after running the same matrix factorization pipeline on this dataset (Figure S17). Due to lower representation of non-inflammatory myeloid cells within the snRNA-seq map (276 cells) compared to the scRNA-seq TLH map (1668 cells), we did not re-capture a strong myeloid-specific strain related varimax factor within the snRNA-seq liver atlas. Alternatively, to further confirm the biological relevance of strain-specific factors found in the total liver homogenate map, we created a gene signature for each strain-related factor (PC5 and PC15) by selecting the top 10 positively and negatively loaded genes for each and used these to score each cell within each strain sample of the snRNA-seq and immune-enriched maps, using the UCell^108^ package (version 1.0.0) (Figures S19E, S19F, and S34).

#### Pathway and gene set enrichment analysis

Gene-set enrichment and pathway analysis methods were used to study the biological signatures represented by each factor. The gene scores corresponding to the factors of interest were selected from the loading matrix to order the list of genes from most to least contribution to the given factor. Pathway enrichment analysis was performed on the ordered list of genes using Gene Set Enrichment Analysis (GSEA))^109^ software from the Broad Institute (software. broadinstitute.org/GSEA) using default parameters (parameters: collapse = false, nperm = 1000, scoring_scheme = weighted, plot_top_x = 20, rnd_seed = 12345, set_max = 200, set_min = 15) the Gene Ontology Biological Process gene set database (Rat_GOBP_Allpathways_no_GO_iea_May_01_2021_symbol.gmt from http://baderlab.org/GeneSets). To identify activated transcription factors, the gProfiler^110^ [https://biit.cs.ut.ee/gprofiler/gost] enrichment tool was used with the CHEA-2016 gene set database [https://maayanlab.cloud/Enrichr/#stats]. GSEA results were visualized using the EnrichmentMap^112^^,^^113^ (version 3.3.2) and AutoAnnotate apps^112^^,^^113^ (version 1.3.4) in Cytoscape^111^ (version 3.8.2).

#### Rat/mouse hepatic zonation correlation analysis

Rat-mouse orthologous genes were identified from the Ensembl database using the biomaRt^114^ packages (version 2.46.2). Using the significantly (q value < 1e-25) differentially expressed genes identified by the Halpern et al.^42^ study for nine layers of mouse liver cells, we selected 107 genes detected in both snRNA-seq rat liver map and Halpern et al. mouse dataset. Expression values of each gene among the hepatocytes clusters of rat datasets and nine layers of mouse liver cells were scaled and centered (separately in rat and mouse) by z-scores. Finally, Pearson correlation was calculated using z-scores across all the selected genes to compare our rat hepatocytes clusters with the nine layers of mouse liver cells in Halpern et al. (Figure 2C)

#### Rat/mouse liver map comparison

Previously published mouse^84^ healthy liver map was downloaded to compare with both TLH and immune-enriched maps (Figure 6I and S4). The specific pathogen-free (SPF1-3) samples from the mouse liver data were selected and pre-processed using Seurat’s standard pipeline. Rat and mouse orthologs were identified using Ensembl biomaRt as described above. In each pairwise cross-species cell type comparison, the one-to-one orthologs in the top 2000 highly variable genes of the two maps were used for Pearson correlation calculation (final number of one-to-one orthologs genes in each comparison: rat total liver homogenate-mouse: 623, rat immune-enriched-mouse: 670). The final heatmap was clustered using Ward’s hierarchical clustering.

#### Total liver homogenate map’s mesenchymal population correlation analysis

The mesenchymal cluster of the total liver homogenate (clusters 7 and 14) and snRNA-seq (cluster 24) were subclustered to perform correlation analysis with mesenchymal subpopulations of Dobie et al., 2019 (Figure S9). The average gene expression of the mesenchymal populations of each single cell transcriptomics map was calculated. Pearson correlation was performed based on the one-to-one orthologs in the top highly variable genes of the maps (final number of one-to-one orthologs genes in each comparison: rat total liver homogenate map - Dobie et al., 2019 : 136, snRNA-seq - Dobie et al., 2019: 324)

#### Intracellular cytokine stimulation assay

To examine the inflammatory potential of myeloid cells in Lewis vs. Dark Agouti rats, NPC fractions generated from 4 pairs of rats were cultured and adhered in culture media consisting of HEPES DMEM (Gibco), 10% fetal bovine serum (Cytiva) and penicillin-streptomycin (Sigma-Aldrich) for 4 hours at 37°C, washed and subsequently stimulated for 6 hours in 12-well tissue culture plates with 1 ng/mL of LPS (Sigma-Aldrich) in the presence of Golgiplug/Brefeldin (Biolegend) and Golgistop/Monensin (Biolegend) solution. The cells were harvested and intracellular secretion of TNFα was examined using flow cytometry.

#### Myeloid purification and cytometric bead array (CBA)

To obtain a myeloid-only immune fraction, 3 pairs of DA and LEW rats were used to generate TLH as detailed above. Three serial centrifugation of 50 g at 4°C for 4 minutes was performed to remove hepatocytes and generate NPC. The resulting NPC was stained with a FITC conjugated anti-rat CD68 recombinant antibody (Miltenyi, Clone:REA237). CD68 expressing cells were then positively selected using an anti-FITC magnetic bead enrichment kit (Stem Cell). The isolation was performed in accordance with the manufacturer’s guidelines, but incubation temperatures of each step were modified to 4°C. Purity was verified via flow cytometry post-enrichment. CD68^+^ cells were adhered in culture media onto 12 well plates at 1.25 million cells per well for 4 h at 37°C, washed, and stimulated with control media or media containing 0.05 ng/mL, 0.1 ng/mL, 1 ng/mL and 10 ng/mL of LPS(Sigma-Aldrich) for 24 hours. The resulting supernatant was collected and frozen down at −80°C for cytometric bead array (LEGENDplex™). Assay was performed as per the manufacturer’s instructions in technical triplicates.

#### Flow cytometry

##### Myeloid purification check and intracellular cytokine staining

Purified cells were first stained with Live/Dead Zombie Aqua dye (Biolegend) to exclude non-viable cells from the analysis and stained with fluorophore-conjugated antibodies against surface markers: anti-CD45-BV786 (BD Bioscience, Clone: OX-1), anti-CD11b-V450(BD Bioscience, Clone: WT.5), and anti-CD3-PE(BD Bioscience Clone: 1F4). The cells were then fixed and permeabilized using the Transcription Factor and Staining Buffer Set (eBioscience), and stained with intracellular antibody monoclonal CD68-AF700 (Novus Biologicals, Clone: ED1). For intracellular cytokine staining, anti-CD3-BV510(BD Bioscience, Clone: 1F4) was also used in lieu of PE. Additional surface antibody anti-CD11a-PE (BD Bioscience, Clone: WT.1) and additional intracellular antibody anti-TNFα-AF488 (AbCam, Clone: EPR21753-109) was also used. Each surface staining and intracellular staining step was accompanied by a rat Fc Blocking step via an anti-CD32(BD Bioscience, Clone: D34-485).

#### Acquisition

All events were acquired on a 5-laser custom BD Fortessa X20 analyzer. The gating strategy for both cell surface markers and intracellular markers was based on Fluorescence Minus One (FMO) controls for each marker. Intracellular cytokine TNFα gating strategy was based on the fluorescence seen in both FMO and the unstimulated control (Figures S35 and S37). Event analysis was performed using FlowJo. Events collected for cytometric bead arrays were automatically gated and analyzed using the manufacturer’s proprietary software (Qognit).

#### Immunohistochemical staining

Paraffin-embedded sections from rat liver were stained by the Pathology Research Program (PRP) at the Toronto General Hospital according to standard histological procedures. Paraffin-embedded rat tissues were stained with antibodies for CD3 (Roche, 2GV6), CD8 (Bio-Rad, OX-8), Hmox1 (Thermofisher, HO-1-1), CD163 (BioRad, ED2) and CD68 (Abcam, ab125212). The stained slides were scanned by the University Health Network Advanced Optical Microscopy Facility using a Leica Aperio AT2 whole slide scanner (Leica Microsystems, Carlsbad CA), and converted into digital images. QuPath software version 0.2.3 software was used to zonate individual lobules into 10 regions of interest, and to perform blinded quantification of stain positive cells on the images. A detailed guide and scripts can be found on protocols.io.^66^

### Quantification and statistical analysis

Visualizations and statistical analysis of assay data were performed using the GraphPad Prism 9 software. All data were presented as means ± SEM and included individual data points representing biological replicates. Statistical details of each experiment are included in the figure legends. The means of technical replicates denoted the measurement of each biological replicate(n). Non-parametric Mann-Whitney (Wilcoxon rank-sum) test was used to evaluate means between two groups. Differences between two groups with multiple treatments were evaluated using a two-way analysis of variance (ANOVA) with a post-hoc Šídák’s test. p values <0.05 indicated statistically significant differences.

## Data Availability

• Raw and processed data files have been deposited at GEO and are publicly available as of the date of publication. Accession numbers are listed in the key resources table. Interactive atlases of the total liver homogenate, immune-enriched, immune-subclustering, snRNA-seq maps and the spatial samples are available through the UCSC Cell Browser^115^ Interface: https://rat-liver-atlas.cells.ucsc.edu Additional microscopy and flow cytometry data beyond what is reported in this paper will be shared by the lead contact upon request. • All original code has been deposited at https://github.com/BaderLab/HealthyRatLiverMap. • Any additional information required to reanalyze the data reported in this paper is available from the lead contact upon request.

## Supplemental information

Document S1. Figures S1–S39 and Table S2

Table S1. Data QC summary table, related to Figures 1, 2, and 6

Table S3. Annotation marker list, related to Figures 1, 2, and 6

Table S4. Differentially expressed gene lists - TLH map, related to Figure 1

Table S5. Varimax/PCA loading tables, related to Figures 2 and 4

Table S6. TF enrichment results - varimax PC5, PC15, related to Figure 5

Table S7. Varimax PC15 (scRNA-seq TLH map) detailed pathway analysis description, related to Figure 5

Table S8. Differentially expressed gene lists - Immune-enriched map, related to Figure 6

Table S9. Differentially expressed gene lists - Immune-subclustering map, related to Figure 6
