# Supplementary material for: A rat liver cell atlas reveals intrahepatic myeloid heterogeneity
Source: iScience. 2023 Oct 14;26(11):108213. doi: 10.1016/j.isci.2023.108213 (PMC10651689; doi:10.1016/j.isci.2023.108213)
Supplement: Document S1. Figures S1–S39 and Table S2 [file mmc1.pdf]

iScience, Volume *26*

Supplemental information

A rat liver cell atlas reveals intrahepatic myeloid heterogeneity

Delaram Pouyabahar, Sai W. Chung, Olivia I. Pezzutti, Catia T. Perciani, Xinle Wang, Xue-Zhong Ma, Chao Jiang, Damra Camat, Trevor Chung, Manmeet Sekhon, Justin Manuel, Xu-Chun Chen, Ian D. McGilvray, Sonya A. MacParland, and Gary D. Bader

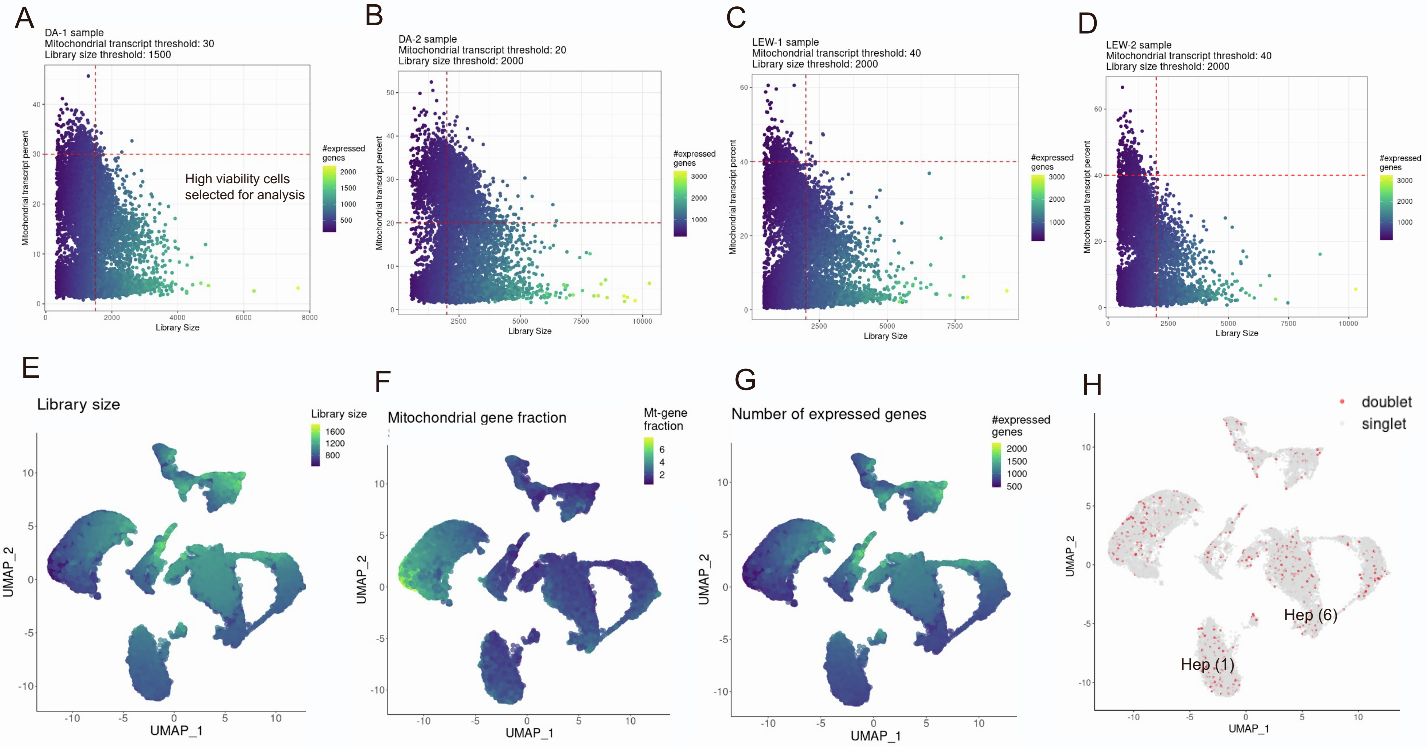

Figure S1. Four healthy rat total liver homogenate scRNA-seq samples quality control and selection of high viability cells. Related to Figure 1. Viable cells were identified from the single-cell gene-expression data based on having a minimum library size of [DA-1, LEW-1, LEW-2] 1500 and [DA-2] 2000 transcripts and a maximum of [DA-1: 30; DA-2: 20; LEW-1: 40; LEW-2: 40] percent mitochondrial transcript proportion. Viable cell selection for A) DA-1 B) DA-2 C) LEW-1 D) LEW-2 rat healthy liver sample. UMAP projection of total liver homogenate map where cells are colored based on E) library size, F) mitochondrial transcript proportion and G) the total number of expressed genes in each cell. Yellow indicates higher values and dark blue indicates a lower value of the QC-covariates. H) A doublet detection algorithm was applied to the rat liver map to identify potential doublets. We decided not to remove these "supposed doublets" since the detected doublet cells had a uniform distribution within the map. Moreover, there are naturally occurring binucleotide hepatocytes in the liver and it's very challenging to distinguish true binucleotide cells from doublets. The distribution of doublets was not denser in the hepatocyte clusters 1 and 6 compared to the other cell populations, rejecting the hypothesis that these clusters have a high density of erythrocyte and hepatocyte doublets.

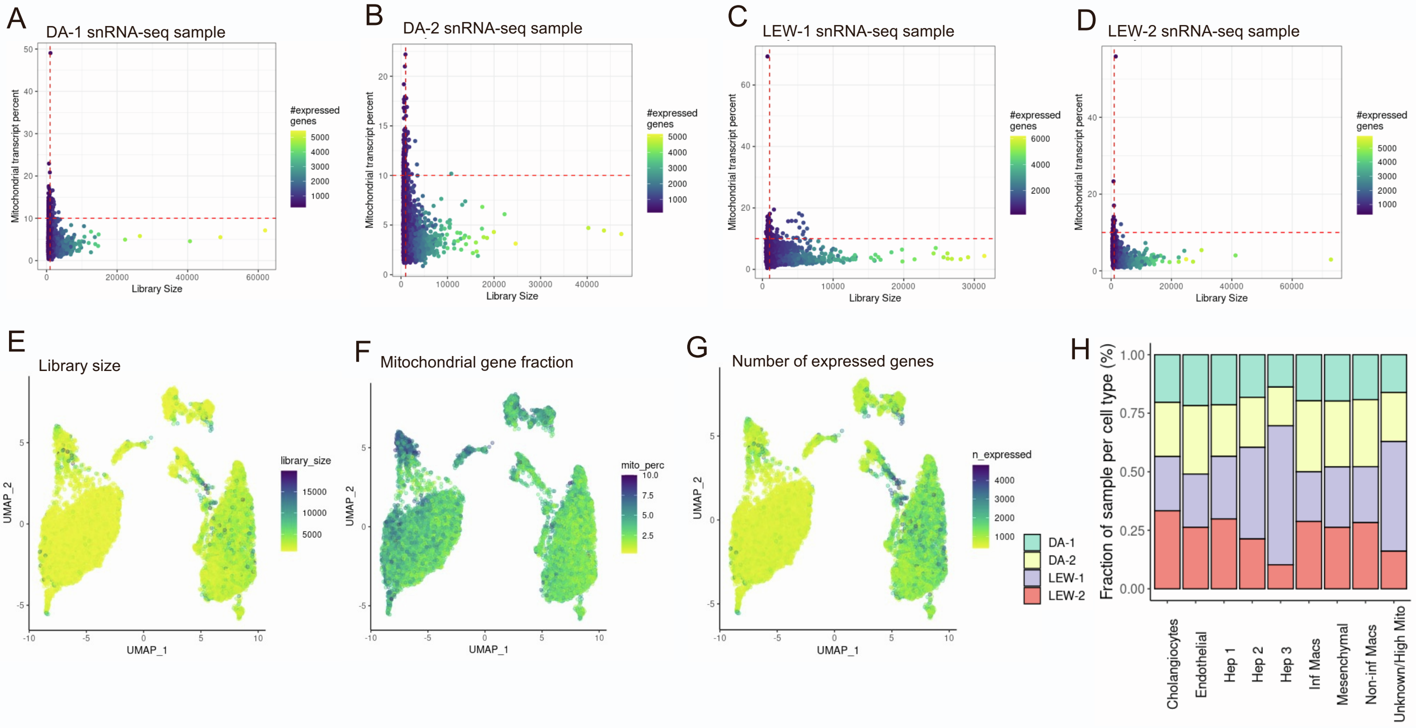

Figure S2. Four healthy rat liver snRNA-seq samples quality control and annotation. Related to Figure 2. Viable cells were identified from the snRNA-seq data based on having a minimum library size of 1000 transcripts and a maximum of 10 percent mitochondrial transcript proportion in the A) DA-1 B) DA-2 C) LEW-1 D) LEW-2 rat healthy liver snRNA-seq samples. UMAP projection of snRNA-seq liver map where cells are colored based on E) library size, F) mitochondrial transcript proportion and G) the total number of expressed genes in each cell. H) Bar plot indicating the relative contribution of input samples to each cluster. All samples are represented in each cluster.

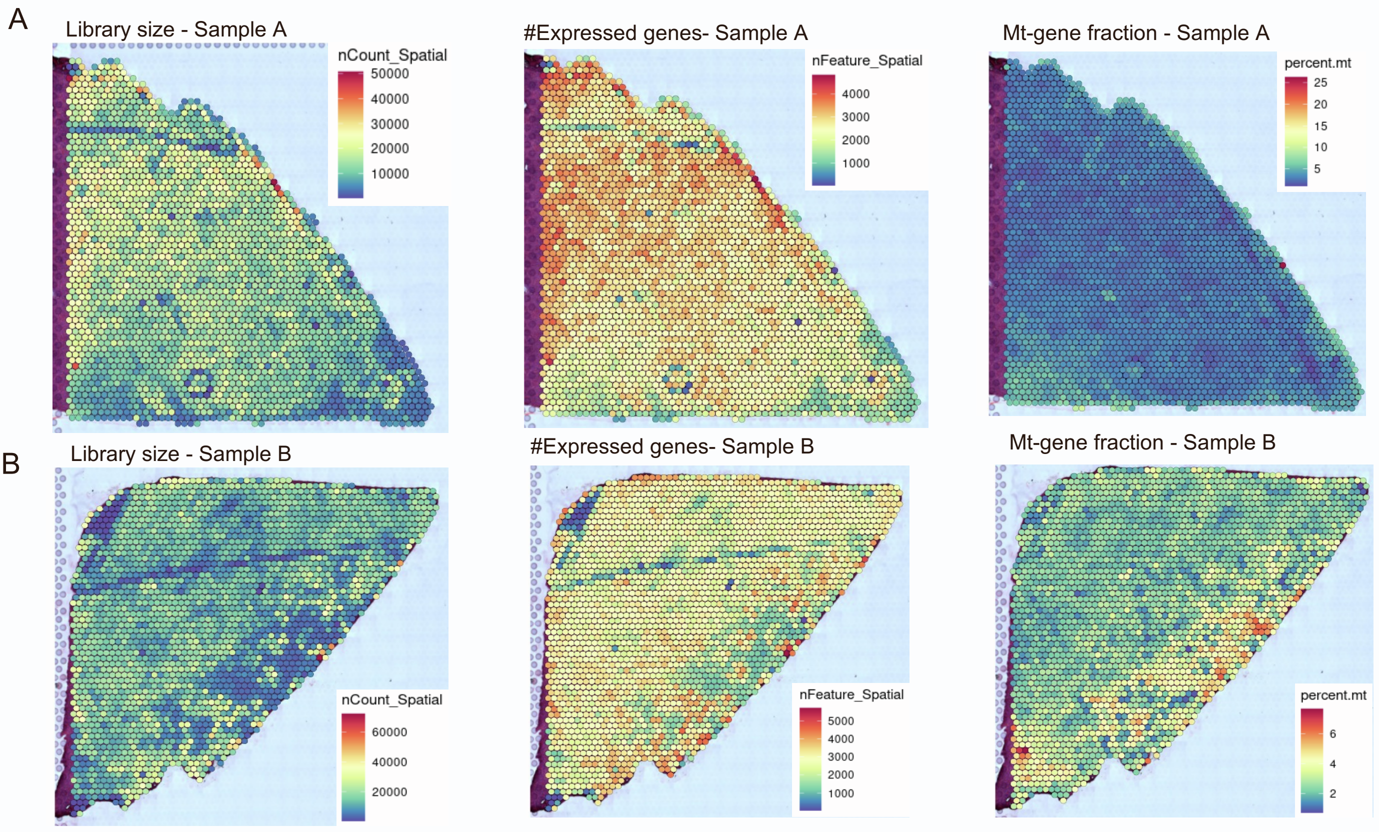

Figure S3. Healthy rat liver spatial transcriptomic data quality control. Related to Figure 2. Projection of quality control covariates across the spatial transcriptomics spots of two healthy Wistar rat liver cryosections, where spots are colored based on library size, the total number of expressed genes, and mitochondrial transcript proportion in each cell in A) sample A and B) B, respectively. Red indicates higher values and dark blue indicates a lower value of the QC-covariates.

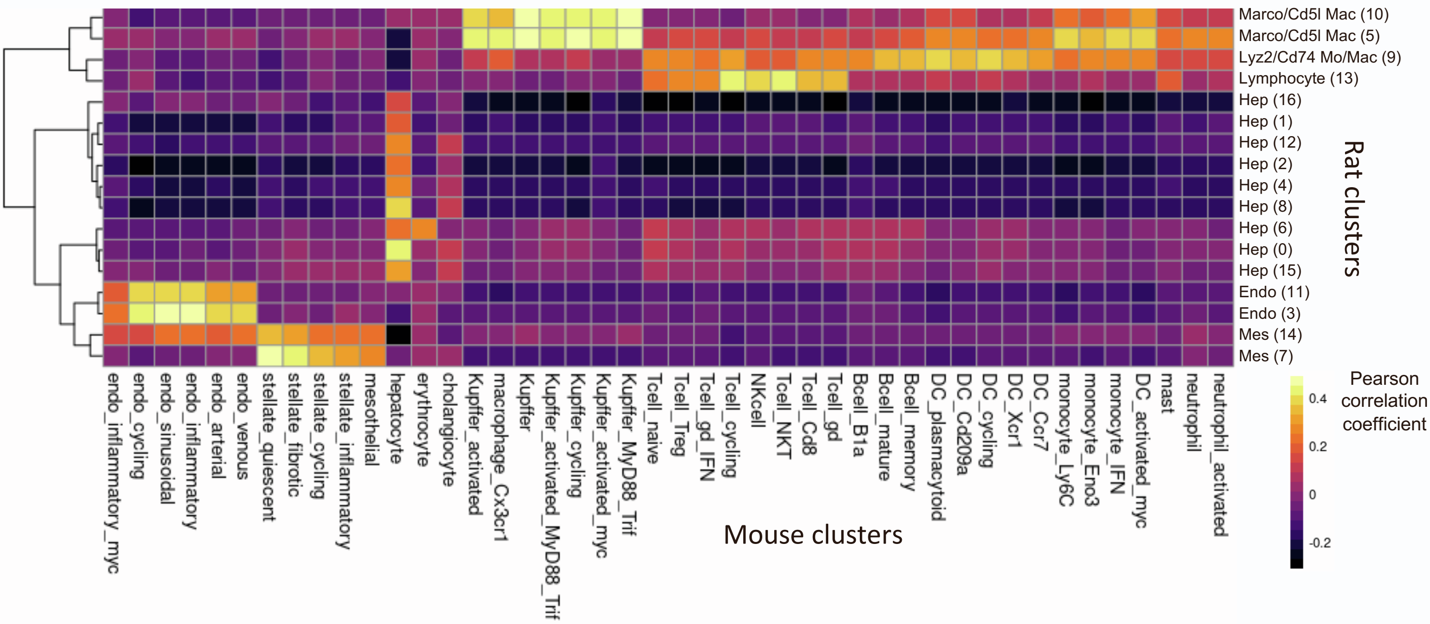

Figure S4. Comparison of rat TLH scRNA-seq and mouse liver atlases. Related to Figure 1. Comparison of the total liver homogenate map and mouse healthy liver map [Kolodziejczyk et al. 2020]. Rows and columns of the correlation heatmap represent the rat and mouse clusters, respectively. The color of the heatmap cells indicates Pearson correlation values between the cluster average expressions. The one-to-one orthologs in the top 2000 highly variable genes of the two maps were used for correlation calculation (see: methods). The comparison indicates a high consistency between the gene expression pattern of hepatic cell types between rats and mice.

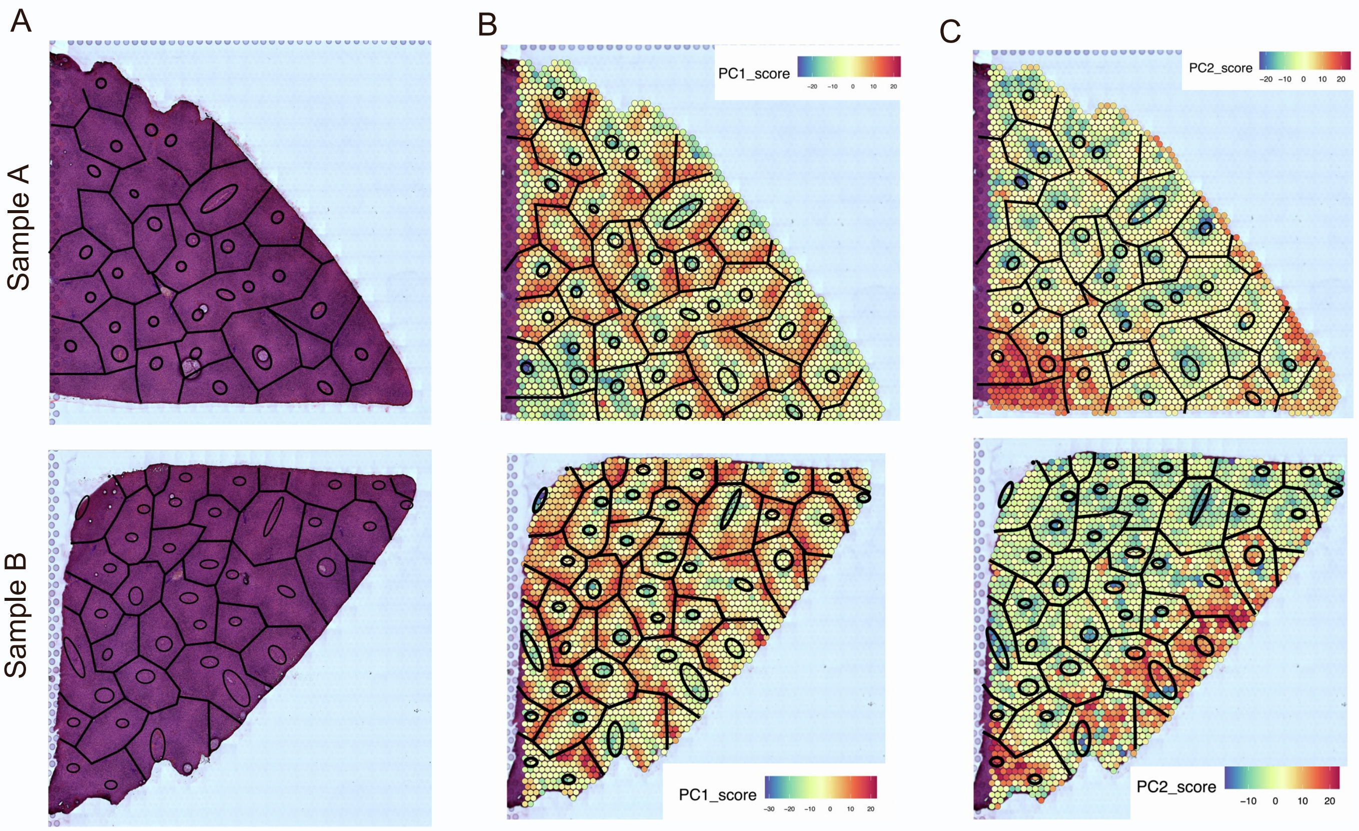

Figure S5. Histological hepatic lobule zonation and corresponding zonation signatures of the spatial transcriptomics samples (Wistar) (10x genomics Visium platform). Related to Figure 2. A) Hematoxylin and Eosin (H&E) staining of healthy rat liver cryosection (Wistar) on the spatial transcriptomics slide was histologically analyzed for hepatic lobule divisions. Pericentral lobule regions were identified and denoted by oval outlines and periportal lobule limits are outlined by straight outlines. Superimposed histologically annotated lobule divisions of H&E onto B) PC1 and C) PC2 signatures.

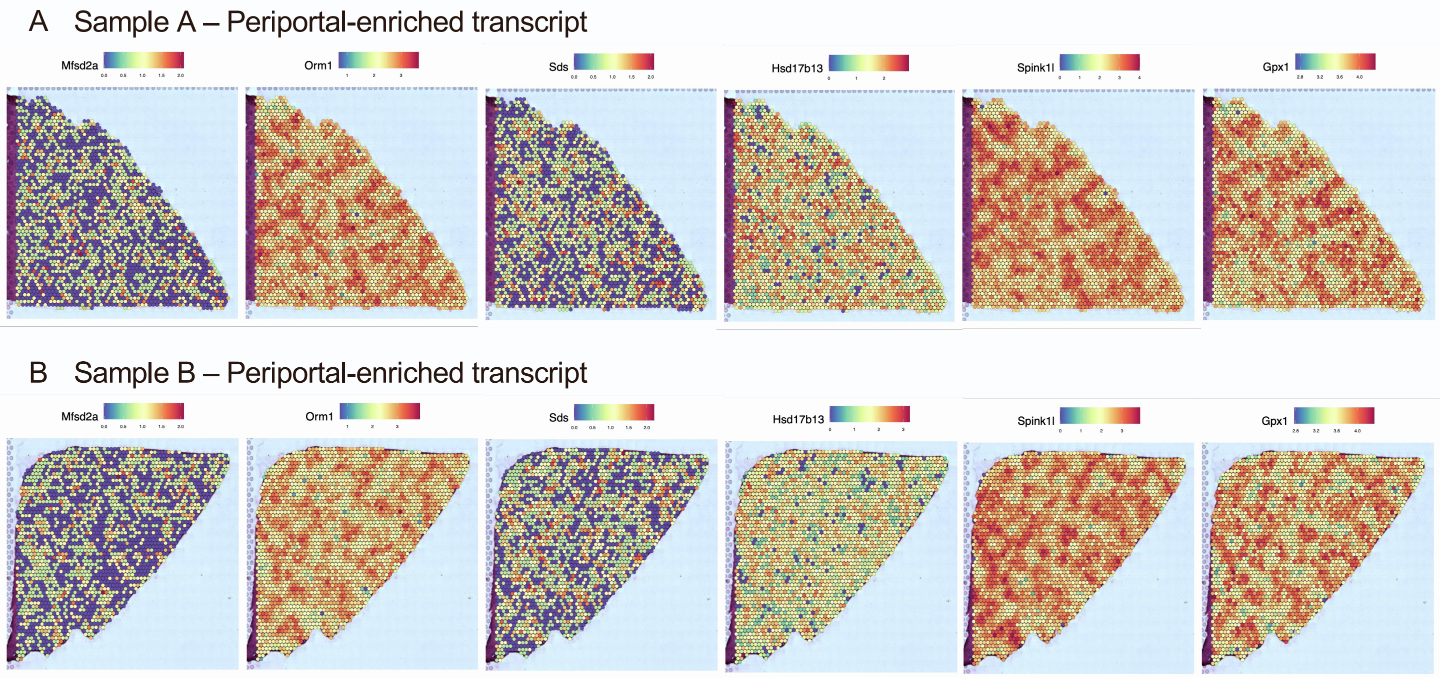

Figure S6. Spatial distribution of landmark periportal markers across the healthy rat liver using spatial transcriptomics (10x genomics Visium platform). Related to Figure 2. Spatial distribution of common (*Orm1*, *Hsd17b13*, *Sds*) and discovered rat (*Mfsd2a*, *Spink1l*, *Gpx1*) markers showing a periportal distribution within hepatic lobules. Red indicates higher values and dark blue indicates a lower expression value in each spot.

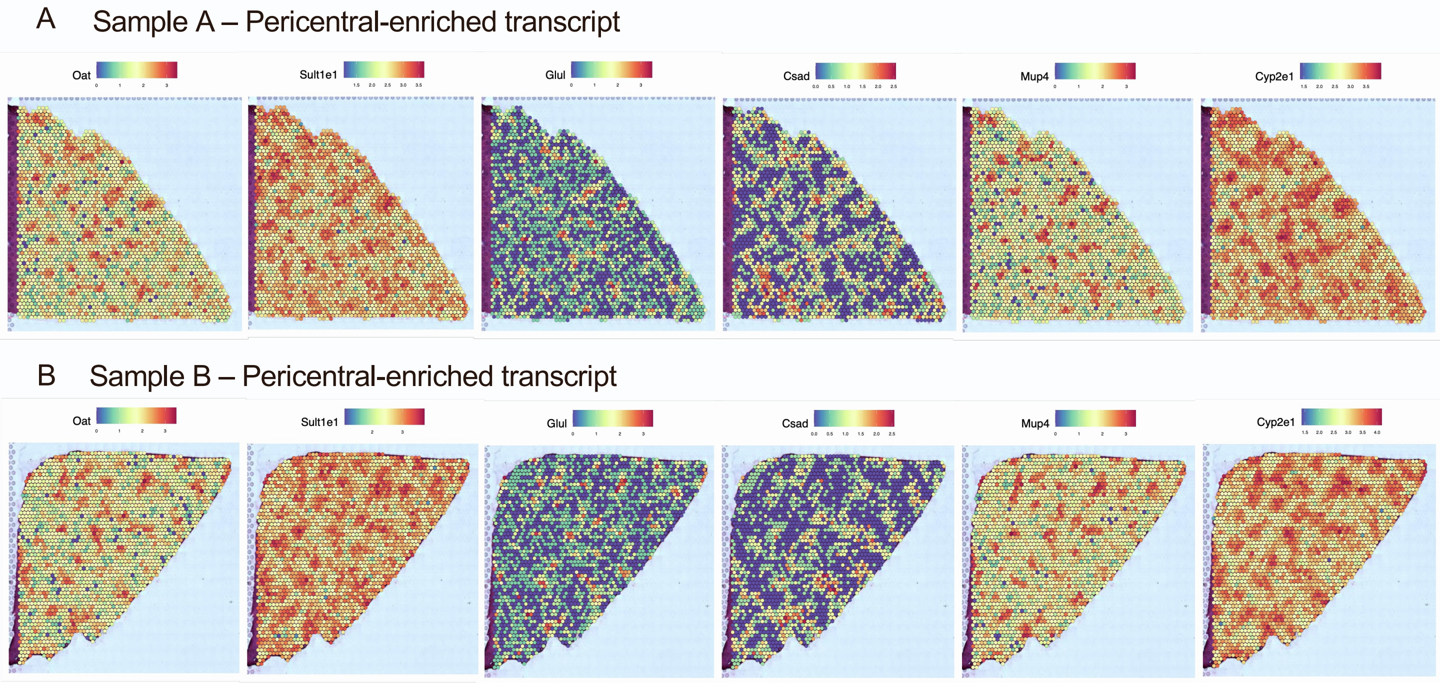

Figure S7. Spatial distribution of landmark pericentral markers across the healthy rat liver using spatial transcriptomics (10x genomics Visium platform). Related to Figure 2. Spatial distribution of common (*Sult1e1*, *Glul*, *Cyp2e1*) and discovered rat (*Oat*, *Csad*, *Mup4*) markers showing a pericentral distribution within hepatic lobules. Red indicates higher values and dark blue indicates a lower expression value in each spot.

Mesenchymal markers (Clusters 7, 14) - TLH map

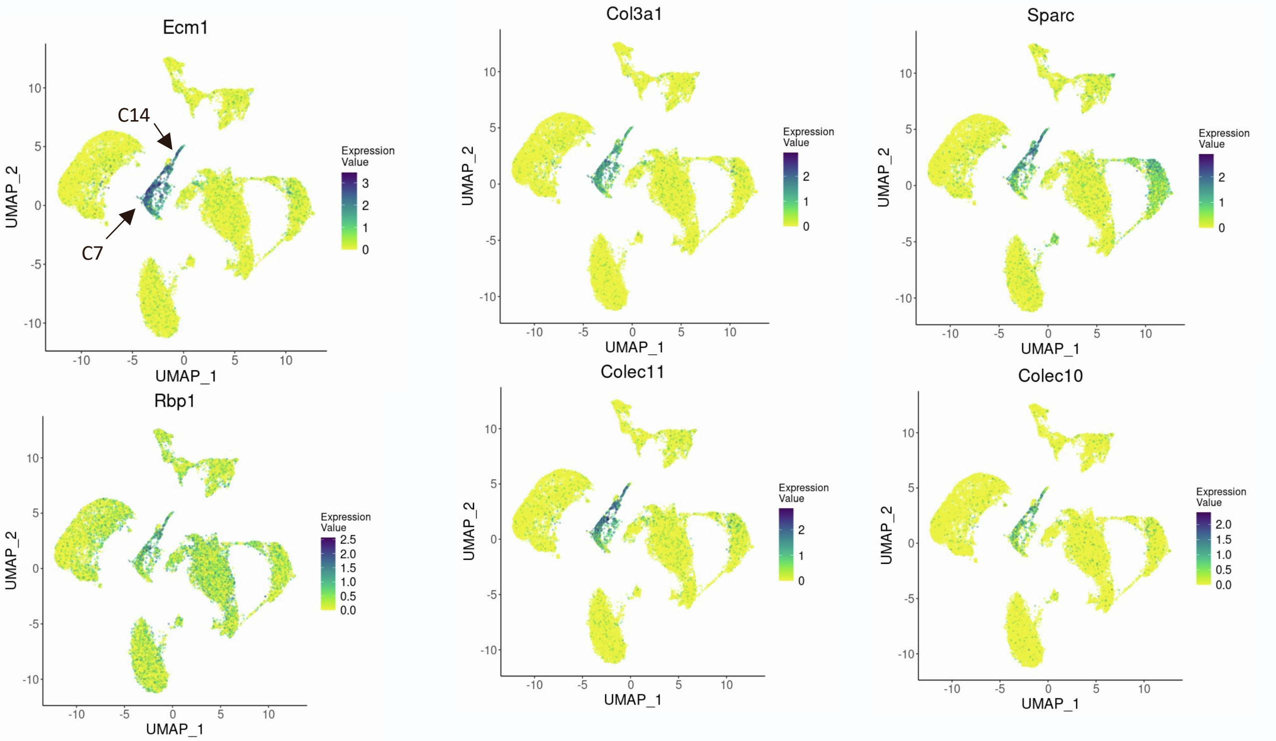

Figure S8. UMAP plots showing the relative distribution of commonly expressed mesenchymal genes (clusters 7, 14) in the healthy rat total liver homogenate map. Related to Figure 1. Legend for the relative expression of each marker from lowest expression (yellow dots) to highest expression (dark blue dots) is placed on the right. C: cluster.

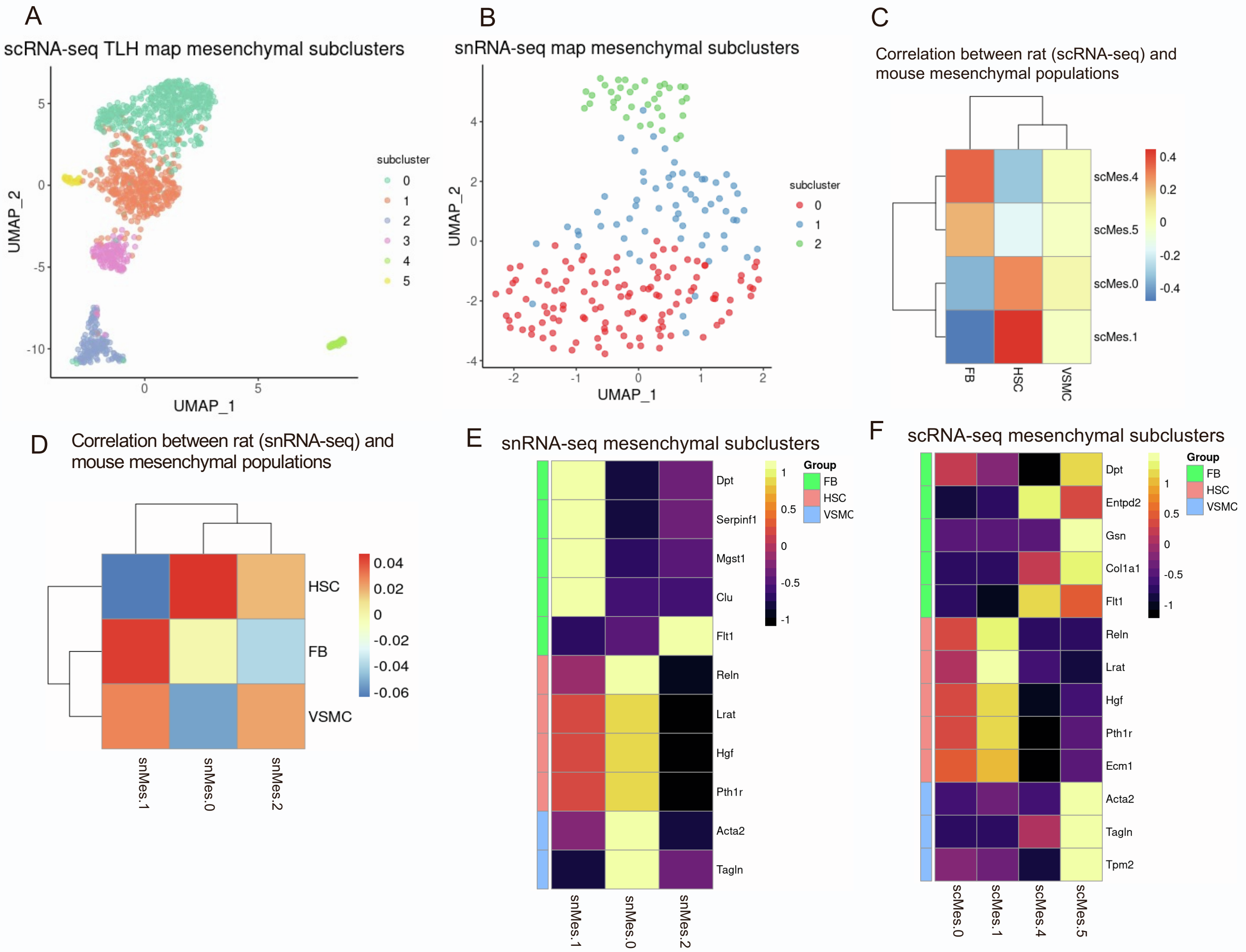

Figure S9. Subclustering and comparison of TLH scRNA-seq and snRNA-seq maps' mesenchymal cells with mice (Dobie et al., 2019). Related to Figure 1. The *Col3a1* and *Ecm1* expressing mesenchymal population (clusters 7 and 14) of the single cell RNA-seq total liver homogenate map and cluster 24 of the single nucleus RNA-seq map were subclustered (clustering resolution = 0.4, 1 respectively) to increase the resolution. UMAP plot of A) scRNA-seq TLH and B) snRNA-seq mesenchymal subclustering colored by the subcluster number. C) Comparison of scRNA-seq rat and mouse (Dobie et al., 2019) mesenchymal subpopulation. Rows and columns of the correlation heatmap represent the rat and mouse subclusters, respectively. The color of the heatmap cells indicates Pearson correlation values between the cluster average gene expressions. Rat mesenchymal subcluster 4 and 5 (scMes-4, scMes-5) are correlated with liver fibroblast (FB) cluster in mice and annotated as fibroblast-like due to enrichment of key fibroblast genes *Dpt*, *Gsn*, *Enptd2* marker and collagen pathway enrichment. scMes-0 and scMes-1 are correlated with mice HSC (hepatic stellate cells) and annotated as HSC-like due to the expression of HSC-associated retinol storage genes and enrichment of lipid metabolism and fibrin clot pathways and inflammation-associated genes. D) Comparison of snRNA-seq rat and mouse mesenchymal subpopulation. Rows and columns of the correlation heatmap represent the mouse and rat subclusters, respectively. snRNA-seq mesenchymal subcluster 1 (snMes-1) indicates a correlation with a mouse fibroblast (FB) cluster and enrichment of key fibroblast genes *Dpt*, *Serpfin1*. snMes-0 was denoted as HSC-like due to similar gene expression patterns of scMes-0, scMes-1 found in scRNA seq. Annotation of rat snMes-2 is of unknown identity due to a lack of correlation and low gene expression. Heatmap of key HSC, FB, and VSMC (vascular smooth muscle cells) markers in E) snRNA-seq, and F) scRNA-seq.

Endothelial markers (Clusters 3, 11) – scRNA-seq TLH map

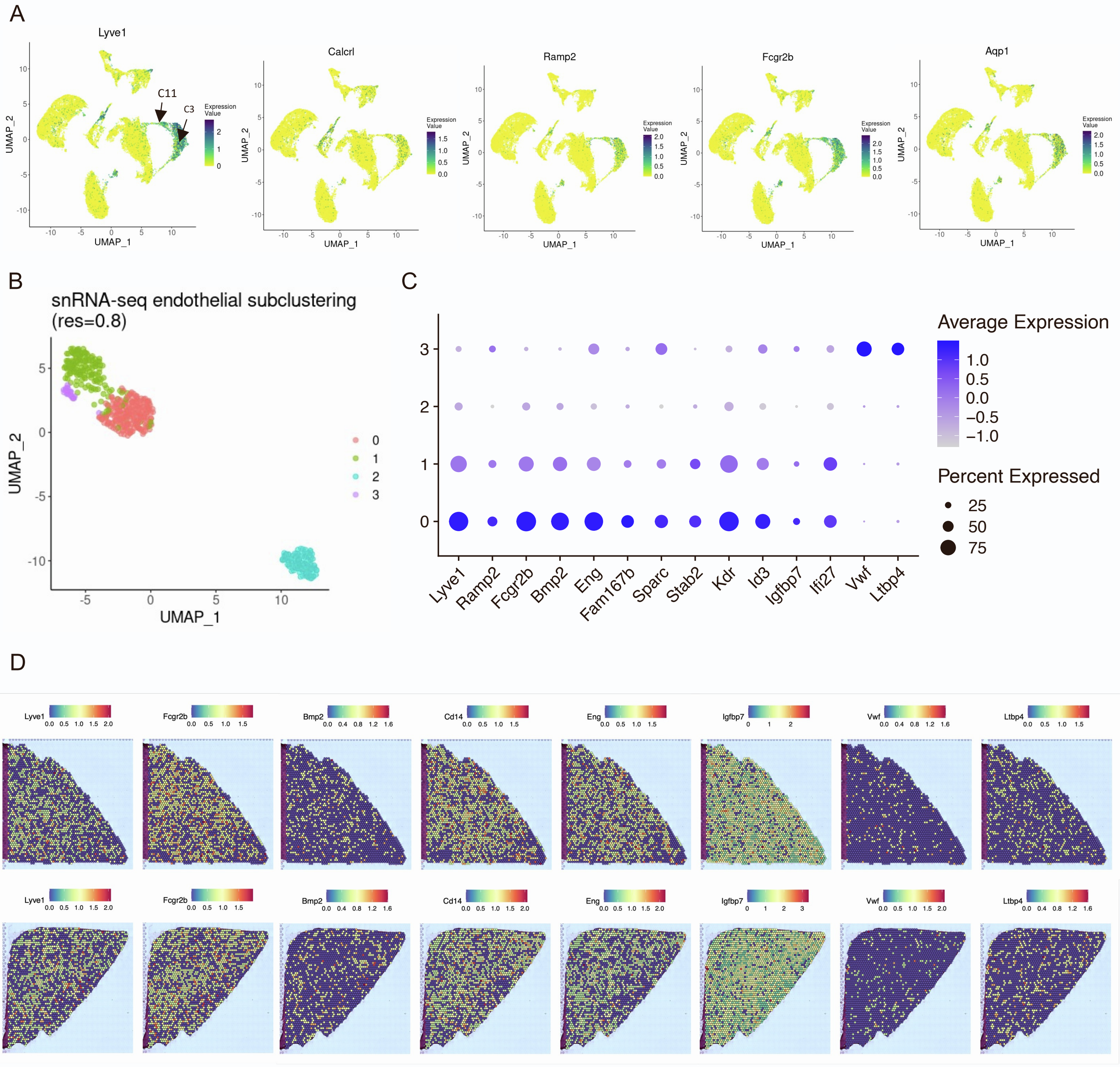

Figure S10. Endothelial population of TLH scRNA-seq and snRNA-seq map. Related to Figure 1, 2 and 3. A) UMAP plots showing the relative distribution of commonly expressed endothelial genes (clusters 3, 11) in the healthy rat total liver homogenate map. Legend for relative expression of each marker from lowest expression (yellow dots) to highest expression (dark blue dots) is placed on the right. B) UMAP projection of endothelial population of the snRNA-seq map colored based on subclustering groups. C) Dot-plot indicating the relative expression of marker genes in each snRNA-seq subcluster population. D) Spatial distribution of endothelial markers in samples A (top row) and B (bottom row). TLH: total liver homogenate, C: cluster

Myeloid markers (Clusters 5, 9) – scRNA-seq TLH map

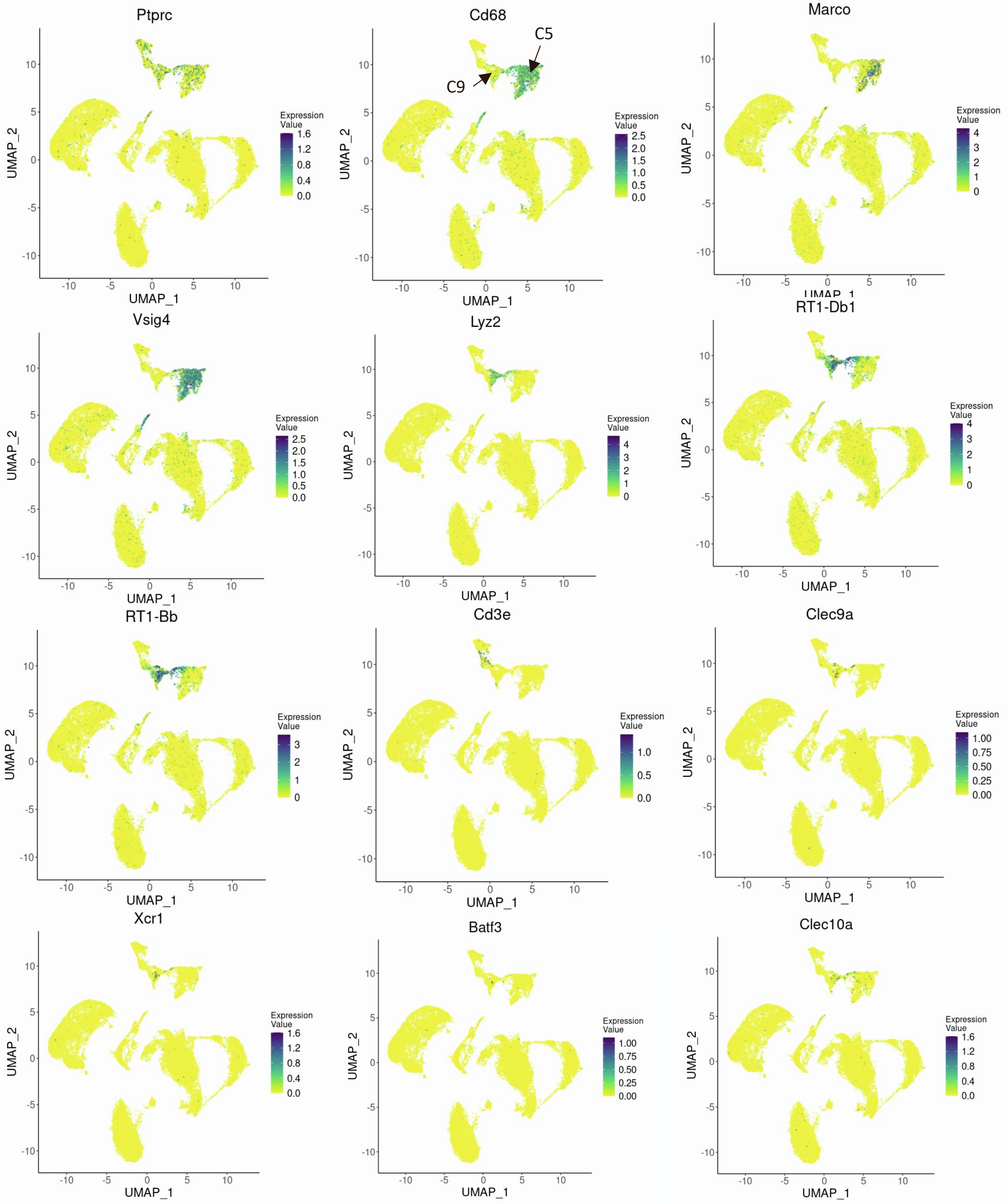

Figure S11. UMAP plots showing the relative distribution of commonly expressed myeloid genes (clusters 5, 9) in the healthy rat total liver homogenate map. Related to Figure 1, and Figure 4. Legend for the relative expression of each marker from lowest expression (yellow dots) to highest expression (dark blue dots) is placed on the right. TLH: total liver homogenate, C: cluster

A

B

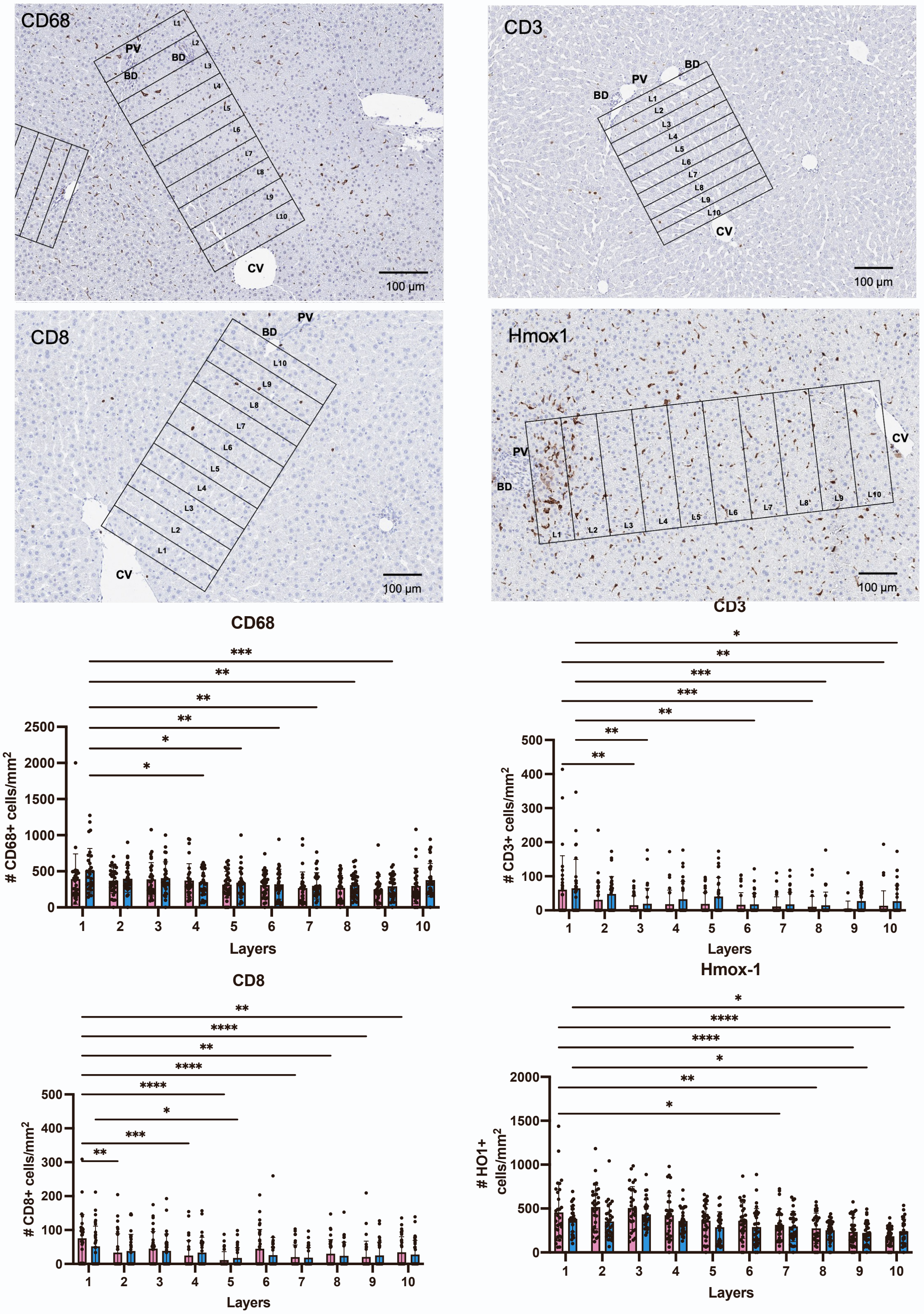

Figure S12. Immune cells zonation trend towards a periportal bias. Related to Figure 3. To validate the enrichment of immune signatures towards the periportal area, histological staining for myeloid cells (CD68, CD163), non-inflammatory myeloid marker (Hmox1), and T cells (CD3, CD8) were performed. A) periportal layers (L1) to pericentral layers (L10) areas were divided into 10 layers per region of interest (ROI) B) summary graph of all 30 ROI's cell positivity per mm^2. Each point represents an individual ROI. Data is represented as mean ± SEM. Statistical significance was determined using a two-way ANOVA followed by a Šídák's multiple comparisons test. (* : p-value < 0.05, ** : p-value < 0.01, *** : p-value < 0.001,**** : p-value < 0.0001) ROI: region of interest, BD: bile duct, CV: central vein, PV: portal vein. n=30

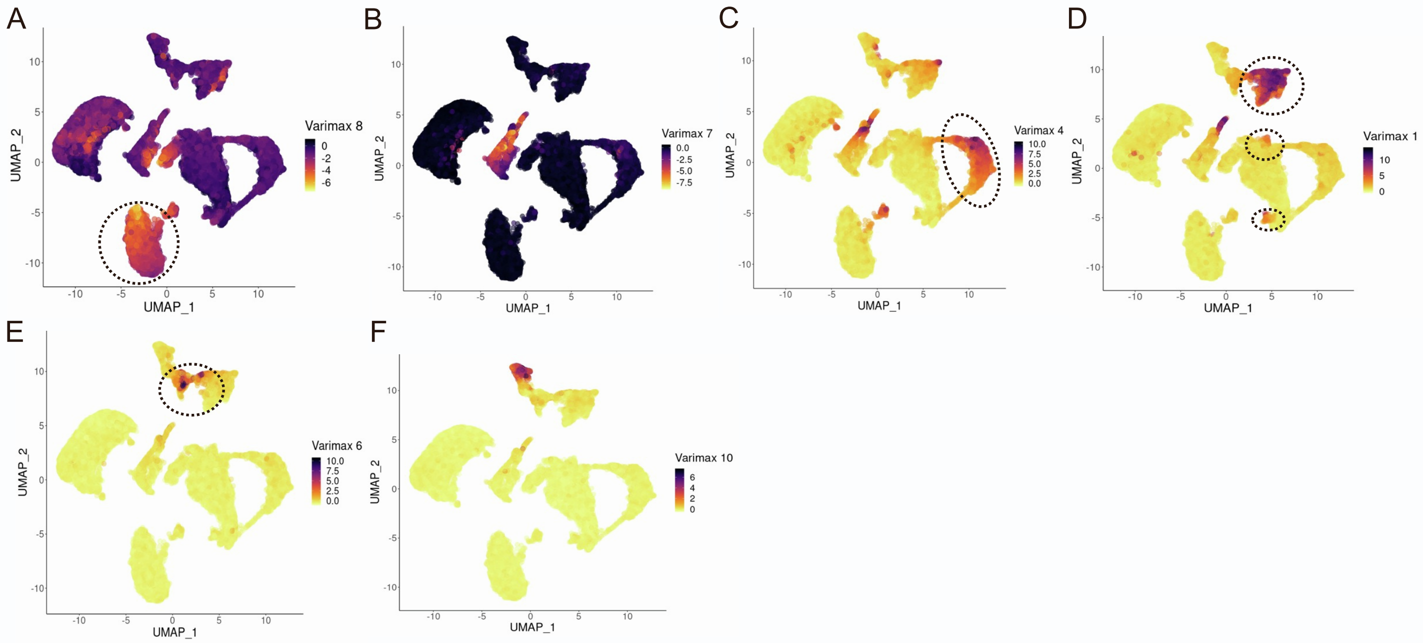

Figure S13. Varimax factors capture the total liver homogenate map cell type signatures. Related to Figure 4. Overlying A) varimax-8 B) varimax-7 C) varimax-4 D) varimax-1, E) varimax-6, F) varimax-10 scores upon scRNA-seq TLH map UMAP plot. Legend for the relative score of each cell from low values (yellow dots) to high (dark purple dots). The Boxplots on the right represent the distribution of varimax factors over each cluster.

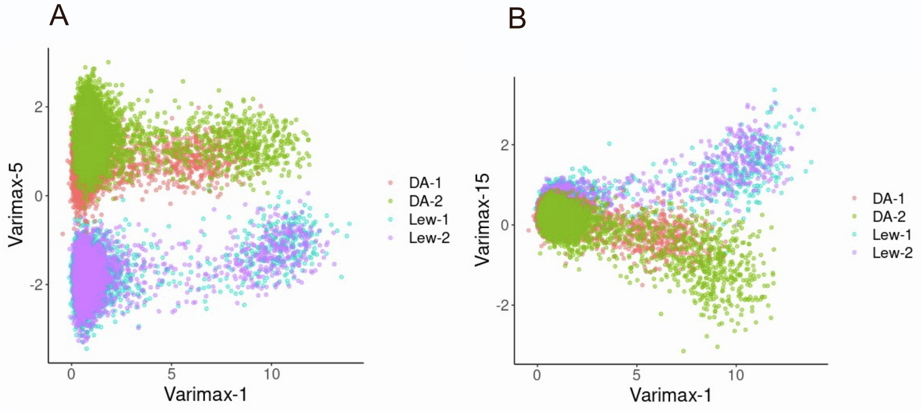

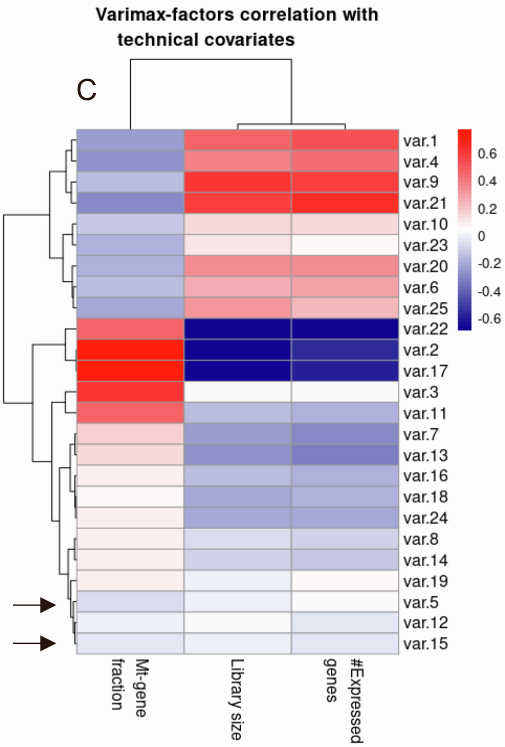

Figure S14. Varimax factors 5 and 15 capture biological, rather than technical variations in the healthy rat liver map. Related to Figure 4. Figures A and B represent the distribution of rat hepatic cells (TLH map) over varimax 5/15 (y-axis) and varimax-1 (x-axis). Although all four samples went through similar sample preparation, cells have been divided based on strain (DA/LEW) and the two samples from the same rat strain are overlapping. Figure C indicates the correlation heatmap of rat liver map (total liver homogenate map) top 25 varimax factors with common technical covariates (Library size, number of expressed genes, and mitochondrial-transcript proportion). The near-zero correlation of varimax 5 and 15 with technical factors suggests that both have been able to capture biological signatures and do not represent technical batch effects. Red and blue indicate positive and negative correlations respectively.

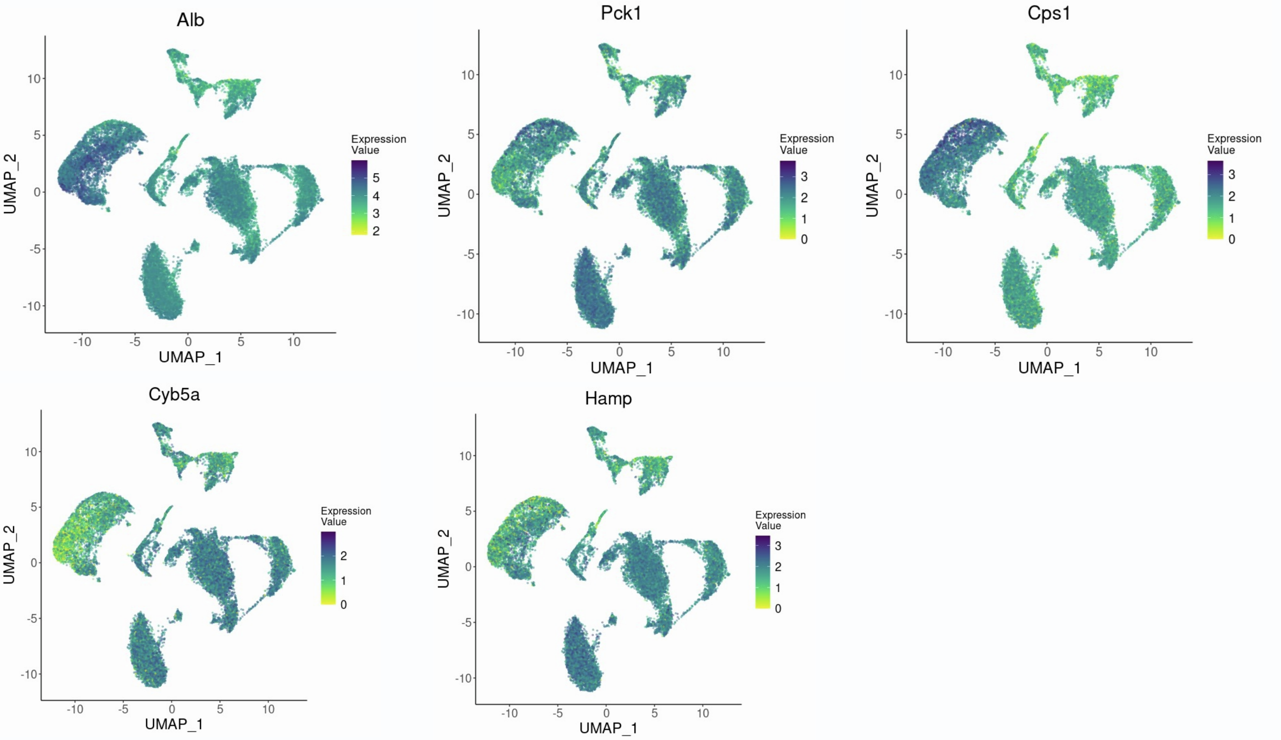

Figure S15. Ambient RNA is released by hepatocyte populations. Related to Figure 1. High expression values of hepatocyte-specific markers (*Alb*, *Pck1*, *Cps1*, *Cyb5a*, *Hamp*) over all the populations within the map confirmed that the RNA released by fragile hepatocytes is the main source of ambient RNA contamination within the map.

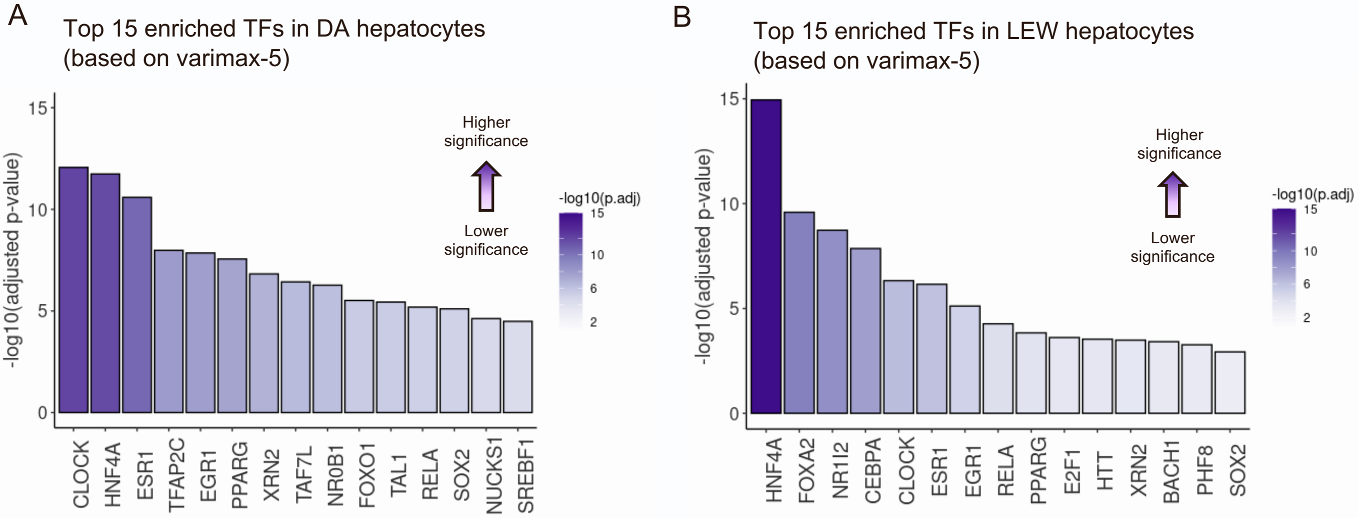

Figure S16. Enrichment analysis of Varimax-5 hepatocyte-related strain-specific differences based on Varimax-5. Related to Figure 4. A) Gene-set enrichment analysis using gProfiler on ChIP-Seq-based ChEA dataset to unravel the activated transcription factors (TF) in DA healthy rat liver. Enrichment results have been calculated based on the top 300 genes on the positive side of varimax-5. Activated TFs are sorted based on –log10(adjusted p-value). Dark purple indicates higher significance. B) Gene-set enrichment analysis using gProfiler on ChIP-Seq-based ChEA dataset to unravel the activated transcription factors (TF) in LEW healthy rat liver. Enrichment results have been calculated based on the top 300 genes on the negative side of varimax-5. Activated TFs are sorted based on –log10(adjusted p-value). Dark purple indicates higher significance.

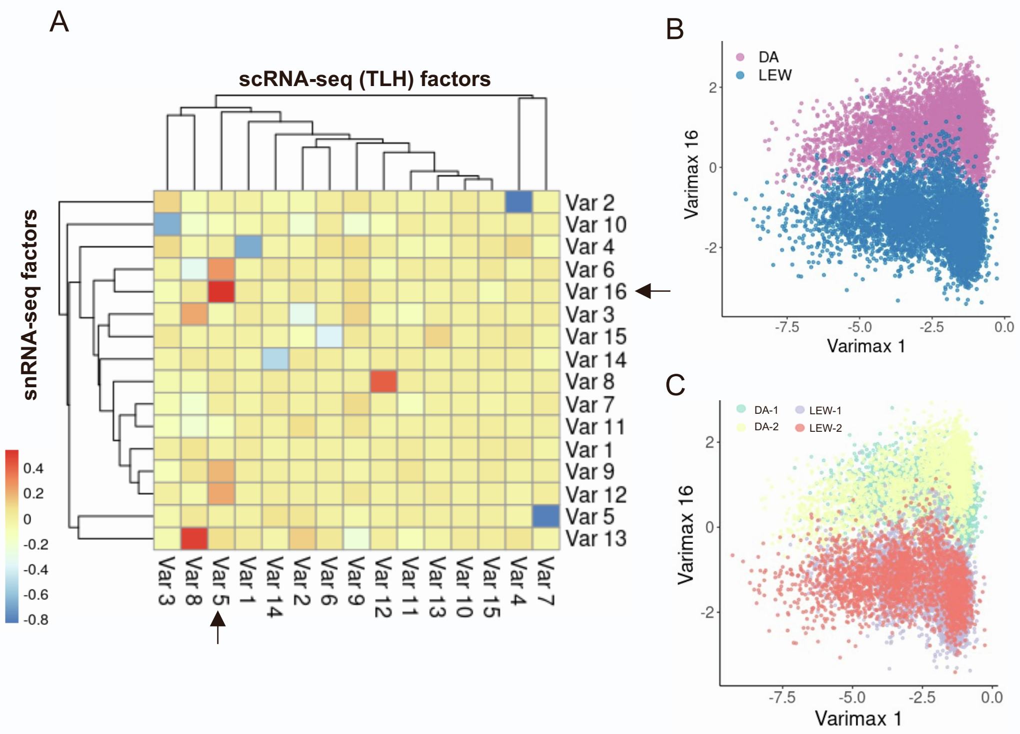

D

DA-associated gene based on varimax-16

Gene	Loading score
Cyp4a2	0.209
RT1-A2	0.157
AY172581.9	0.147
AY172581.24	0.107
Dhrs7l1	0.094
Ahcy	0.089
Ces1d	0.088
Creg1	0.088
Cdh1	0.087
RT1-CE10	0.083

LEW-associated gene based on varimax-16

Gene	Loading score
Ugt2b	-0.144
Cyp2d5	-0.098
Cyp2c6v1	-0.091
Cyp2c13	-0.081
Apoa4	-0.078
RT1-A1	-0.076
Fabp1	-0.070
AABR07026311.1	-0.070
Itih3	-0.069
Cyp8b1	-0.068

Figure S17. Hepatocyte strain variations are rediscovered in the snRNA-seq rat liver map. Related to Figure 4. A) The correlation between the varimax factors identified using the scRNA-seq TLH map (columns) and the factors identified based on applying varimax to the snRNA-seq (rows) atlas. The Hepatocyte-specific varimax factor 5 in the TLH map is highly correlated with the Var-16 factor from the snRNA-seq map. Projection of cells over Varimax factors 1 and 16 indicates that although all four samples went through similar sample preparation, B) cells have been divided based on strain (DA/LEW) and C) the two samples from the same rat strain are overlapping. D) The top 10 genes on the top and bottom of the Varimax-16 loading list mainly contain known hepatocyte markers, confirming that Varimax-16 has captured hepatocyte-specific strain differences. Genes with high positive scores (top table) are associated with the DA strain and genes indicating negative loading scores (bottom table) are LEW-related. Mitochondrial genes have been removed from the list. The absolute loading scores indicate the contribution of each gene to the corresponding factor.

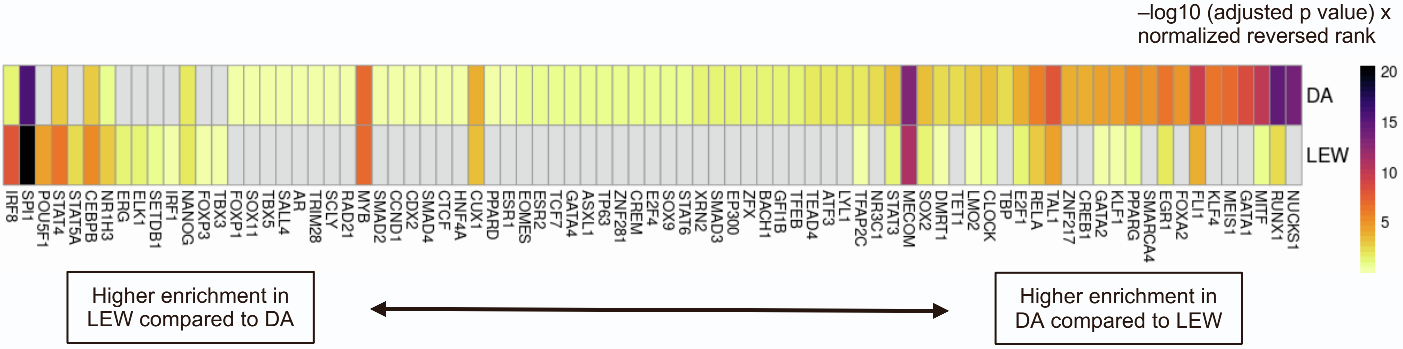

Figure S18. Differential enrichment of strain-specific TFs based on Varimax-15. Related to Figure 5. Heatmap representing the ranked enrichment of strain-related transcription factors in DA and LEW rats. The TFs on the right, indicate higher enrichment in the DA strain compared to LEW and the TFs on the left represent the reversed enrichment. Significantly enriched TFs in both strains have been scored based on -log10 (adjusted p value) normalized reversed rank. For each TF, enrichment scores in DA and LEW were subtracted from each other and the resulting values were used to sort the TFs. Purple and yellow represent higher and lower enrichment scores, respectively. The grey color indicates that the TF is not enriched in the given strain.

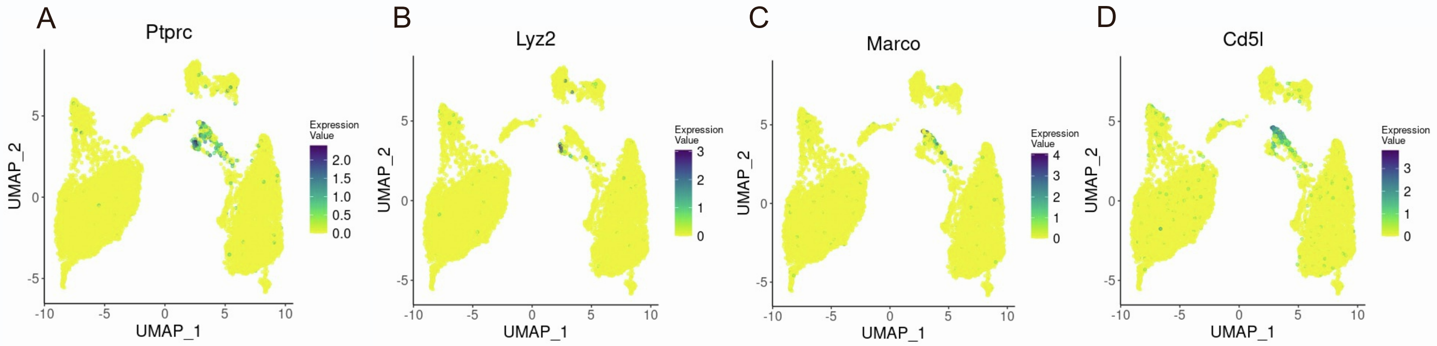

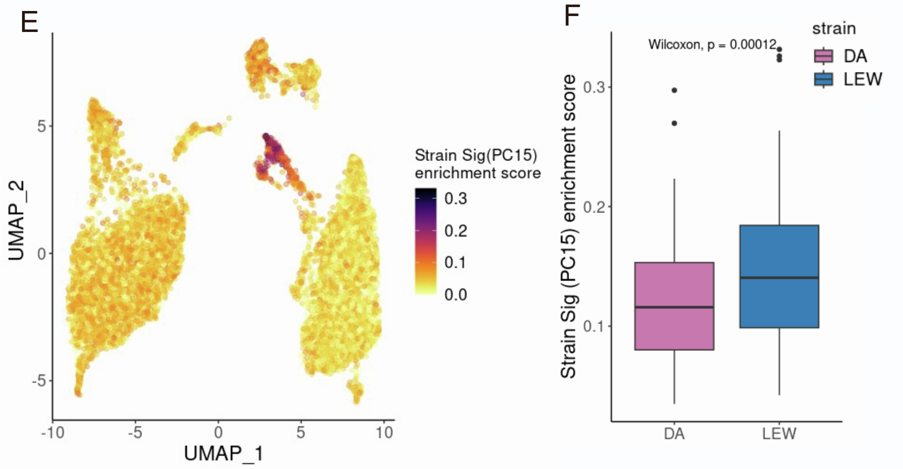

G

genes	p value	logFC(LEW/DA)
Ugt2b	5.98e-11	2.50
* Itgal	1.77e-06	2.49
* RT1-A1	6.05e-11	1.28
LOC103695118	1.99e-05	2.08
* Timp2	2.39e-03	3.14
Amacr	7.98e-04	2.51
Slc35c2	4.03e-04	2.23
Apoa4	1.68e-04	1.93
* RT1-T24-3	4.96e-06	1.36
Mcfd2	2.89e-04	1.58
* Lilrb3l	3.96e-03	2.25

Figure S19. snRNA-seq profiling of rat liver validates myeloid strain variations. Related to Figure 2 and Figure 5. Expression pattern of known myeloid and immune marker genes A) *Ptprc*, B) *Lyz2*, C) *Marco*, and D) *Cd5l* on the UMAP of all rat liver snRNA-seq. Dark green represents high expression values. E) Overlying the varimax PC15 signature enrichment score upon rat snRNA-seq UMAP. Cells with high enrichment of this geneset are colored dark purple and cells with zero enrichment are indicated as yellow. The distribution of the enrichment scores over snRNA-seq clusters confirms that similar to the findings based on TLH samples, the strain-specific signature represented by varimax PC15 is myeloid-specific. F) Boxplot indicating the enrichment scores of varimax PC15 signature within the myeloid population of each strain. In line with our predictions based on the scRNA-seq TLH map, varimax-15 top positive genes are more enriched in the snRNA-seq LEW myeloid compared to DA (Wilcoxon-test p value= 0.00012). G) Performing differential expression between the two strains within the myeloid subcluster indicates the presence of multiple strain-related genes identified by varimax PC15. The table indicates the top DE genes between the strains. The genes present in the top varimax PC15 loadings (top 10: *Itgal*, *RT1-A1*, *Timp2*, *Lilrb3l*; top 130: *RT1-T24-3*) are labeled with a red asterisk. The unlabelled top genes are mainly hepatocyte transcripts, resulting from ambient RNA. Data are represented as mean ± SEM with each dot representing a single nuclei.

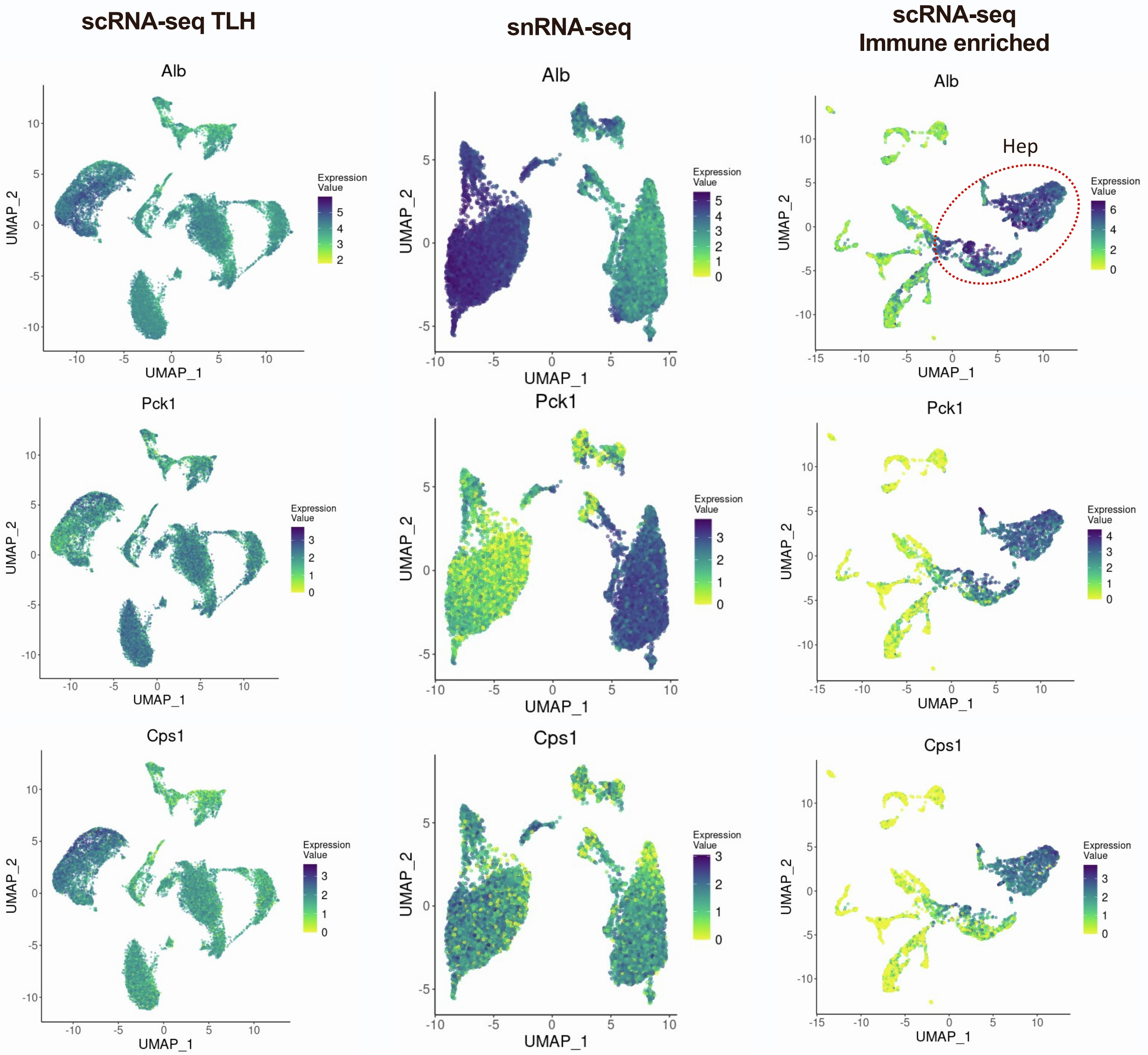

Figure S20. Hepatocyte-released ambient RNA decreased in the immune-enriched map. Related to Figures 1, 2 and 6. Expression distribution of hepatocyte markers *Alb*, *Pck1*, and *Cps1* in the scRNA-seq total liver homogenate, snRNA-seq, and scRNA-seq immune-enriched maps indicate that hepatocyte markers are mainly specific to hepatocyte clusters in the immune-enriched map compared to the other two rat liver atlases**.** This comparison confirms that the level of ambient RNA has been decreased in the immune-enriched map likely due to the additional washing steps.

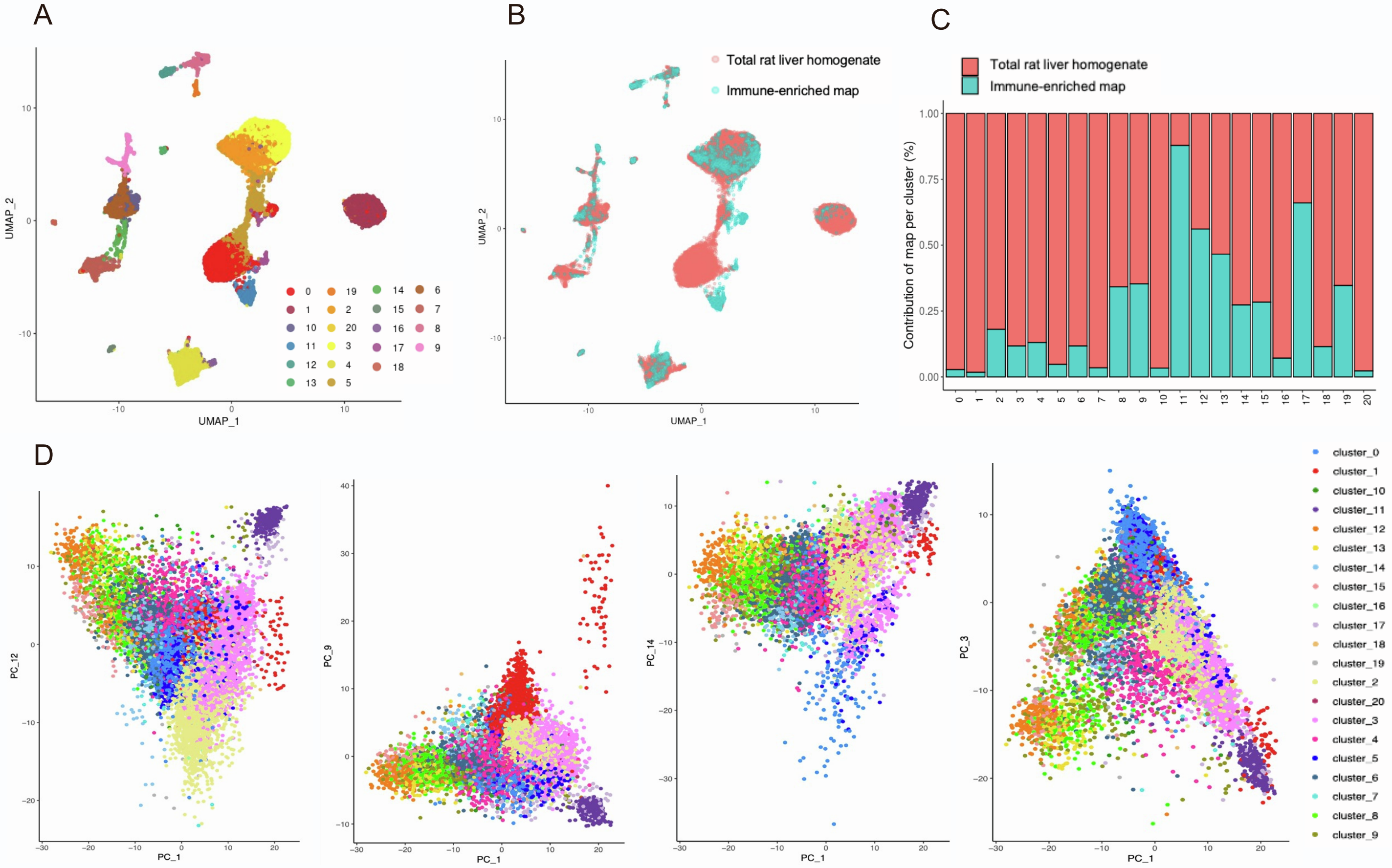

Figure S21. The total liver homogenate and immune-enriched samples could not be well-integrated with standard methods. Related to Figure 1 and Figure 6. The initial four total liver homogenate samples and the two immune-enriched rat livers were merged, batch corrected, and clustered. A) UMAP projection of the merged (total liver homogenate and immune-enriched) rat samples where cells that share similar transcriptome profiles are grouped by colors representing unsupervised clustering results. B) Labeling UMAP projection of cells based on the input sample set indicates that cells from the total liver homogenate and immune-enriched maps do not form well-integrated clusters. C) Bar plot indicating the relative contribution of input samples to each cluster. Although low clustering resolution has been chosen, Clusters 0, 1, 5, 7, 10, and 20 are restricted to cells from the total liver homogenate map and cluster 11 is mainly represented by immune-enriched, indicating that the two maps have not been well-integrated. D) The varimax pipeline was applied to the merged total liver homogenate and immune-enriched maps. Distribution of cells over varimax-PC1 (x-axis) and varimax-PC12, varimax-PC9, varimax-PC1, and varimax-PC3 on the y-axis are represented as examples. As indicated, it's not possible to associate the resulting factors with any cluster or covariate. The varimax pipeline has been unable to separate the sources of variation within the merged map into interpretable factors and therefore, was ineffective.

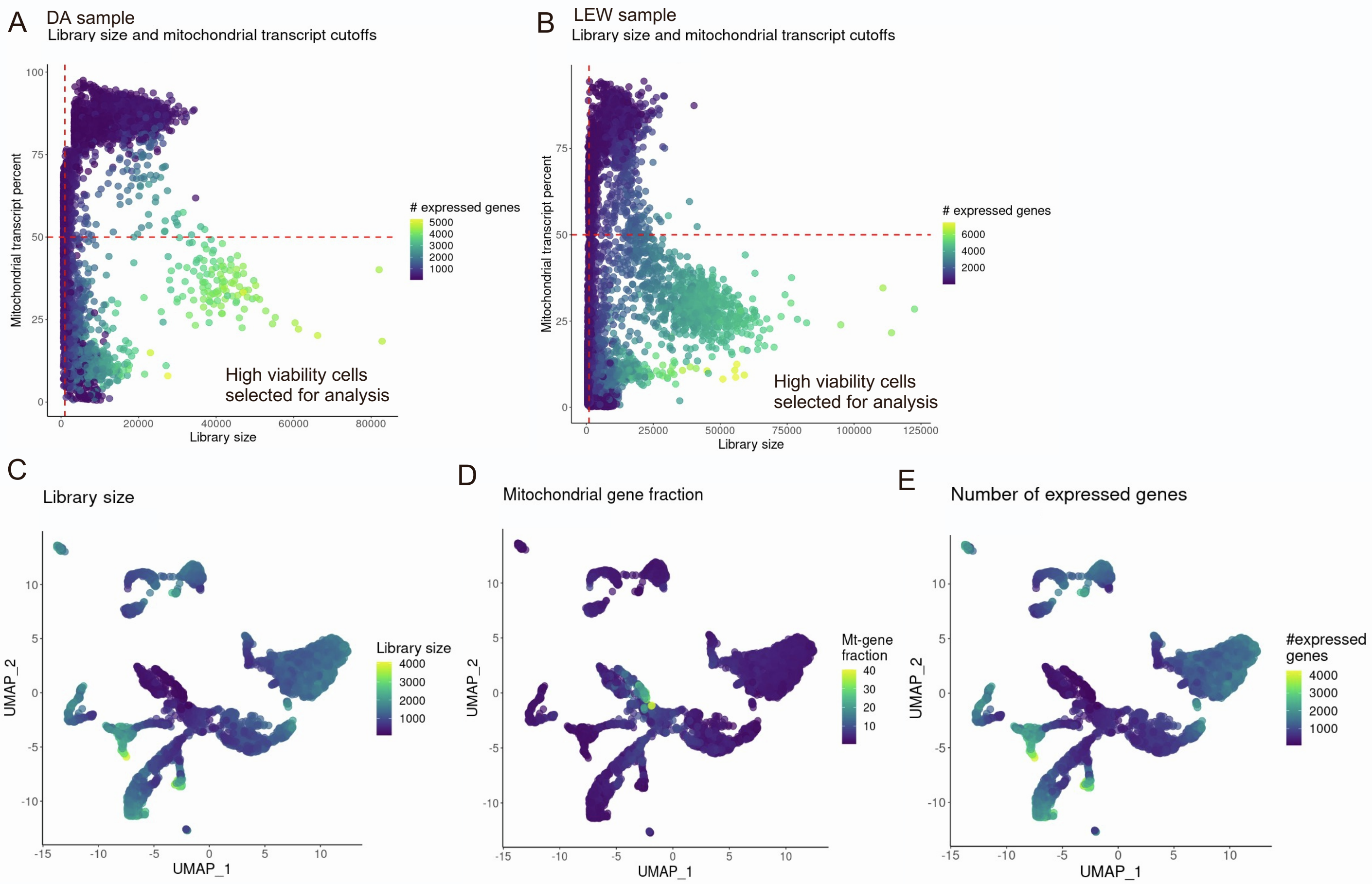

Figure S22. Immune-enriched liver samples quality control and selection of high viability cells. Related to Figure 6. Viable cells for the immune-enriched map were identified from the single-cell gene-expression data based on having a minimum library size of 1000 transcripts and a maximum of 50% mitochondrial transcript proportion. A) DA B) LEW rat healthy liver sample. UMAP projection of immune-enriched map where cells are colored based on C) Library size, D) mitochondrial transcript proportion and E) the total number of expressed genes in each cell. Yellow indicates higher values and dark blue indicates lower values of the QC-covariates.

Cd3+ T cell markers (Cluster 10) – Immune-enriched map

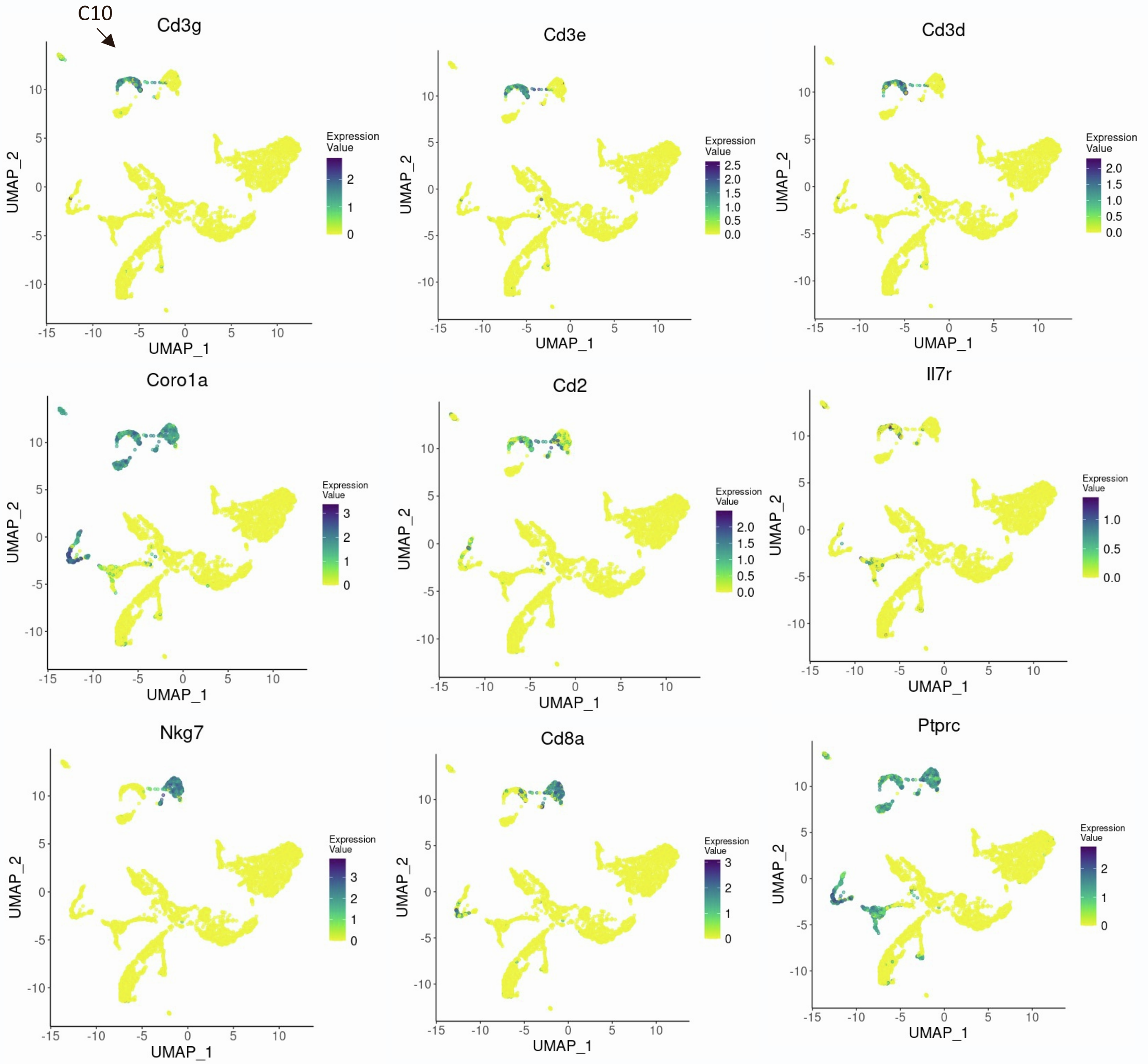

Figure S23. UMAP plots showing the relative distribution of commonly expressed Cd3+ T cell genes (cluster 10) in the healthy rat immune-enriched map. Related to Figure 6. Legend for the relative expression of each marker from lowest expression (yellow dots) to highest expression (dark blue dots) is placed on the right. C: cluster

B cell markers (Cluster 12) – Immune enriched map

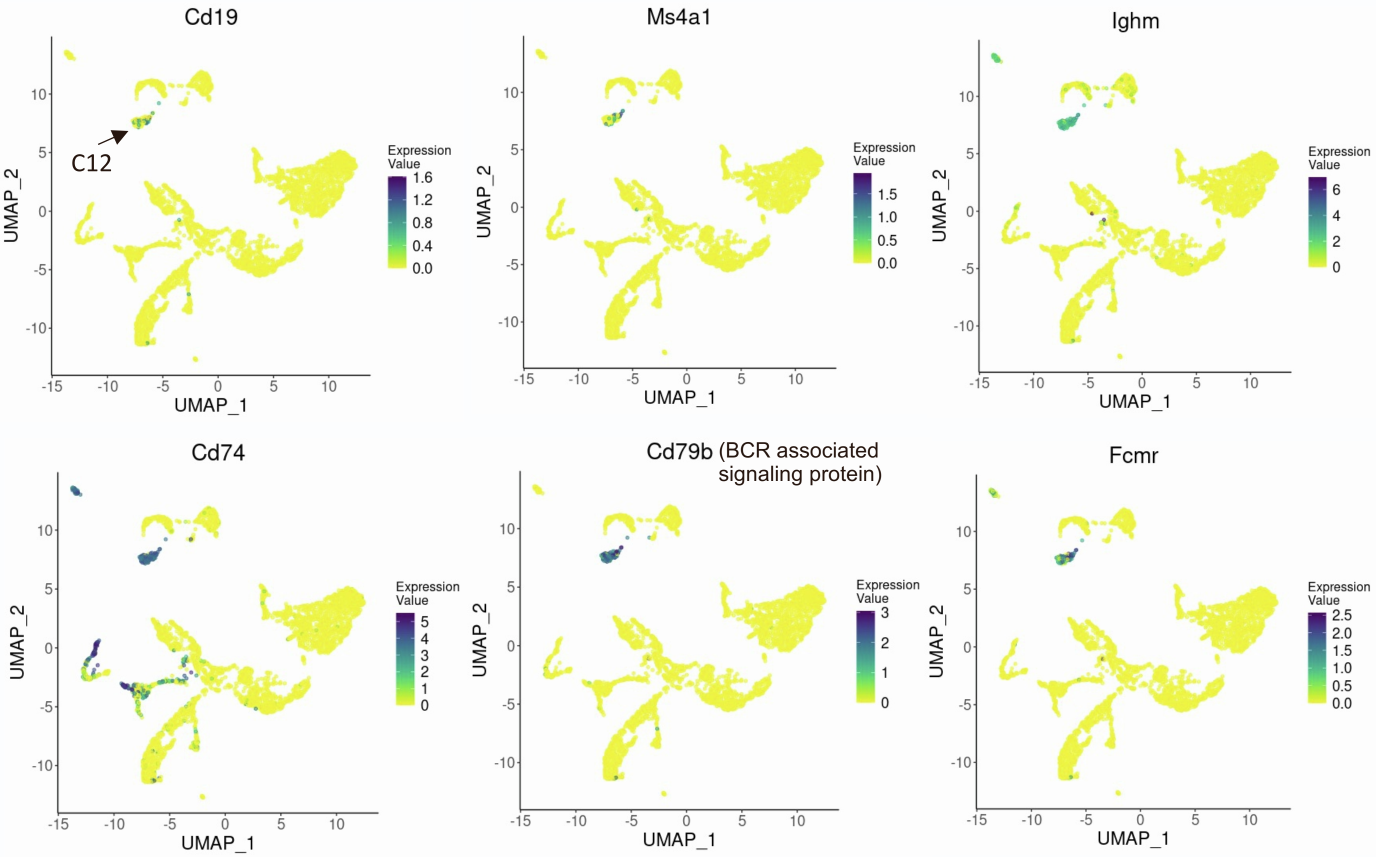

Figure S24. UMAP plots showing the relative distribution of commonly expressed B cell genes (cluster 12) in the healthy rat immune-enriched map. Related to Figure 6. Legend for relative expression of each marker from lowest expression (yellow dots) to highest expression (Purple dots) is placed on the right. C: cluster

pDC-enriched markers (Cluster 17) – Immune enriched map

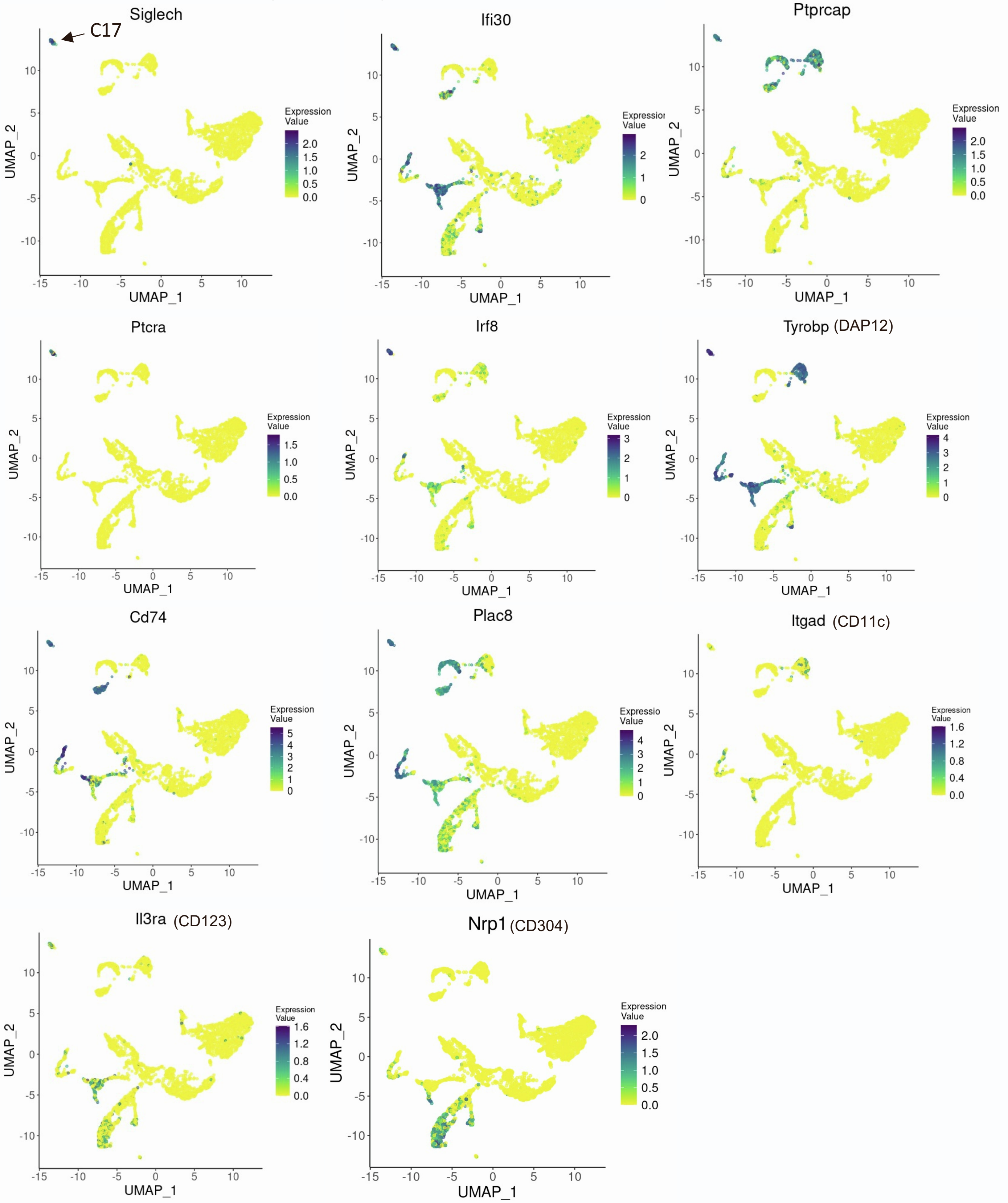

Figure S25. UMAP plots showing the relative distribution of commonly expressed pDC genes (cluster 17) in the healthy rat total liver homogenate map. Related to Figure 6. Legend for the relative expression of each marker from lowest expression (yellow dots) to highest expression (dark blue dots) is placed on the right. C: cluster

cDC cell markers (part of cluster 11) – Immune enriched map

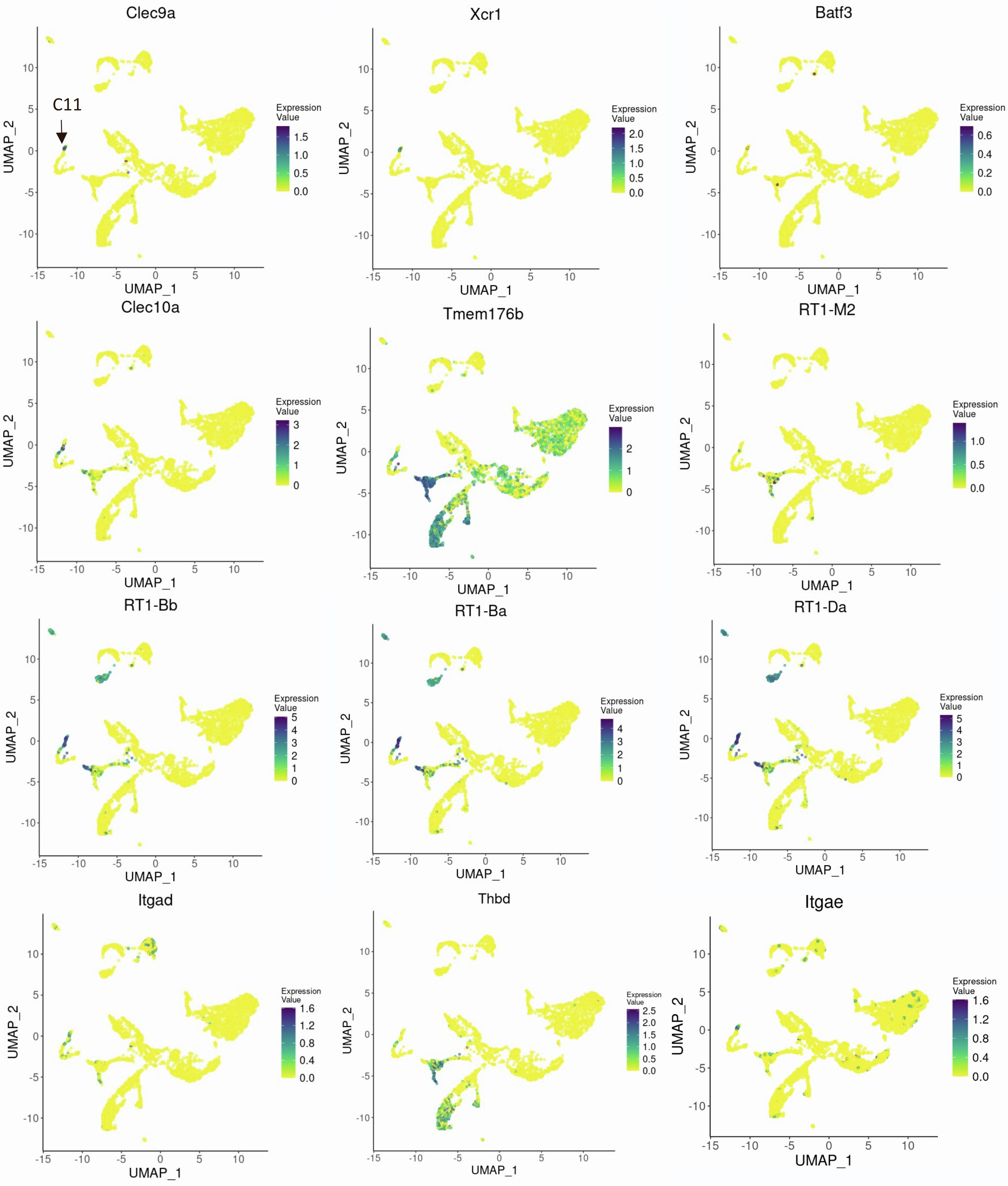

Figure S26. UMAP plots showing the relative distribution of commonly expressed cDC genes (cluster 11) in the healthy rat total liver homogenate map. Related to Figure 6. Legend for the relative expression of each marker from lowest expression (yellow dots) to highest expression (dark blue dots) is placed on the right. C: cluster

NK-like cell markers (Cluster 7) – Immune enriched map

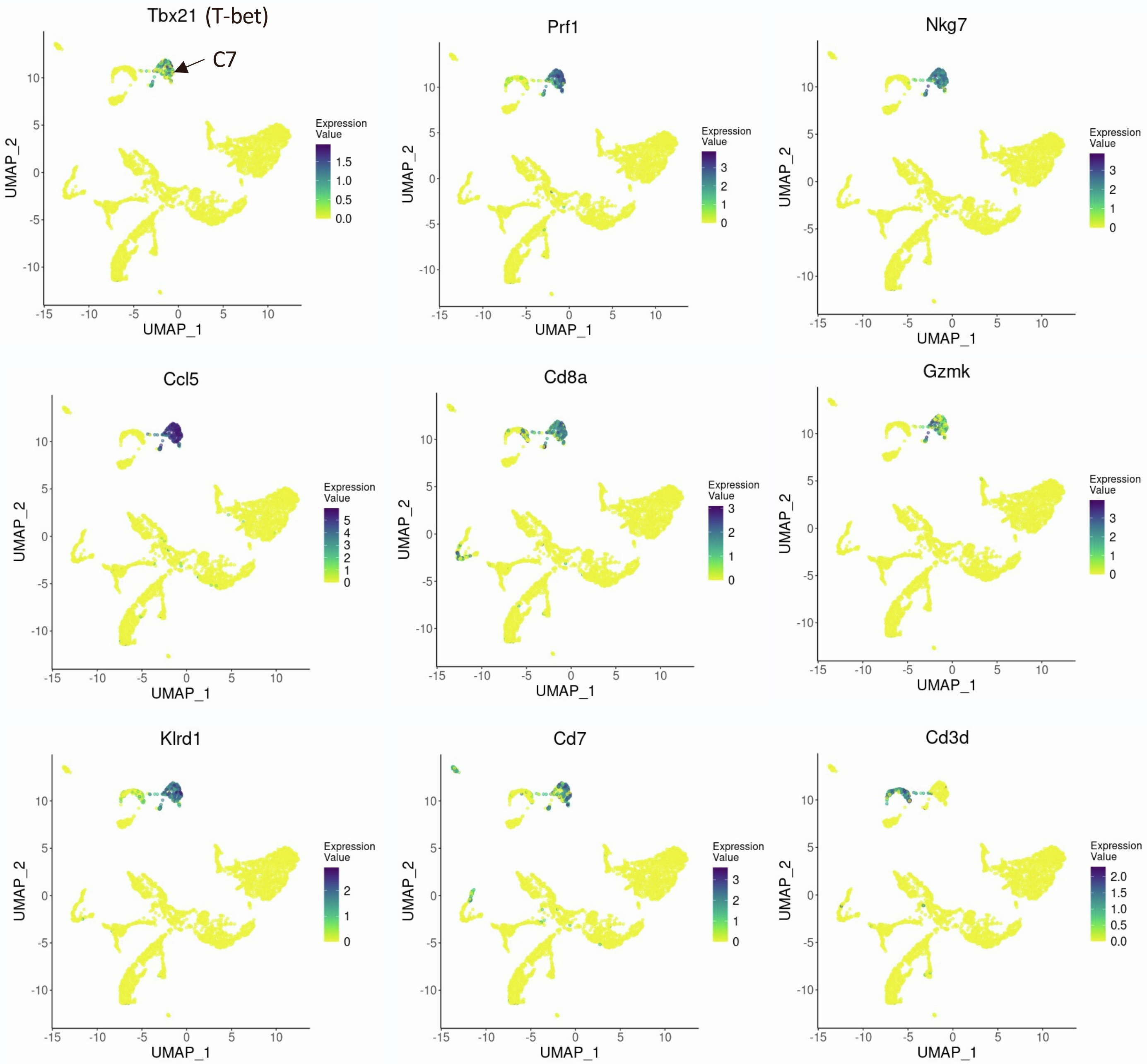

Figure S27. UMAP plots showing the relative distribution of commonly expressed NK-like cell genes (cluster 7) in the healthy rat total liver homogenate map. Related to Figure 6. Legend for the relative expression of each marker from lowest expression (yellow dots) to highest expression (Purple dots) is placed on the right. C: cluster

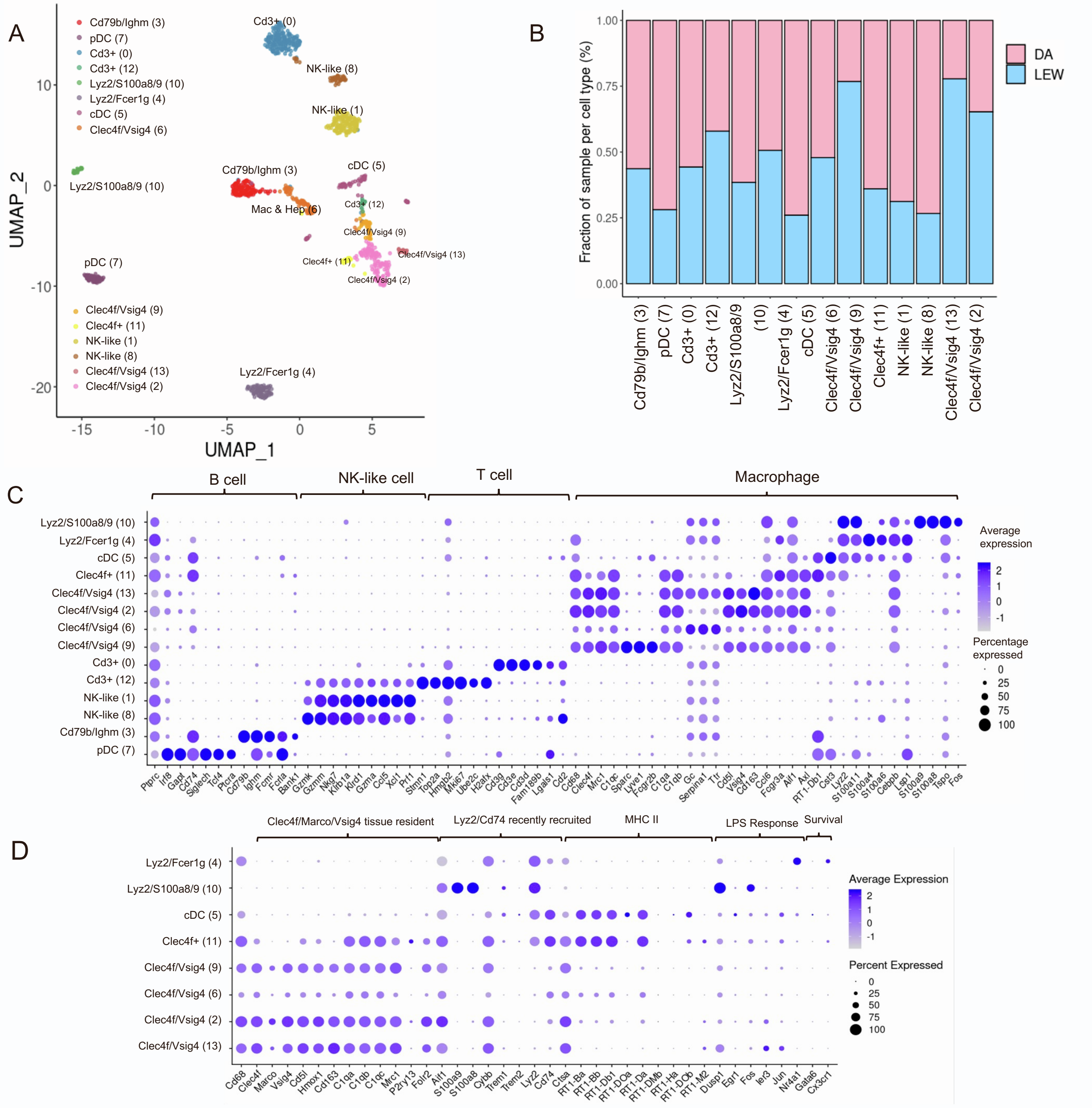

Figure S28. Subclustering of *Ptprc*+ cell types indicates 14 immune cell populations. Related to Figure 6. A) UMAP projection of the *Ptprc*+ subpopulation of the immune-enriched samples. The *Ptprc*+ clusters of the immune-enriched map were subclustered to provide a more detailed representation of immune subtypes. Colors indicate different subclusters. B) Bar plot indicating the relative contribution of input samples to each subcluster. All samples have been represented in each of the subclusters. C) Dot-plot indicating the relative expression of marker genes in each immune subcluster. Evaluation of the top markers subclusters 6 and 9 indicate that a few LSECs and hepatocytes might have been mixed within the immune clusters. The x-axis represents marker genes, and the y-axis represents the annotated subclusters within the map. The size of the circle indicates the percentage of cells in each population which express the marker of interest and the color indicates the average expression value (dark purple: high expression, grey: low expression). D) Dot plot demonstrating the expression pattern of different macrophage markers. Macrophage markers have been grouped into 6 categories (non-inflammatory, inflammatory, MHC II, LPS response, and survival) based on their potential role in macrophage function. The interpretation of the size and color of the circles is the same as in previous dot plots.

T cell markers (subcluster 0, 12) – Immune subclustering

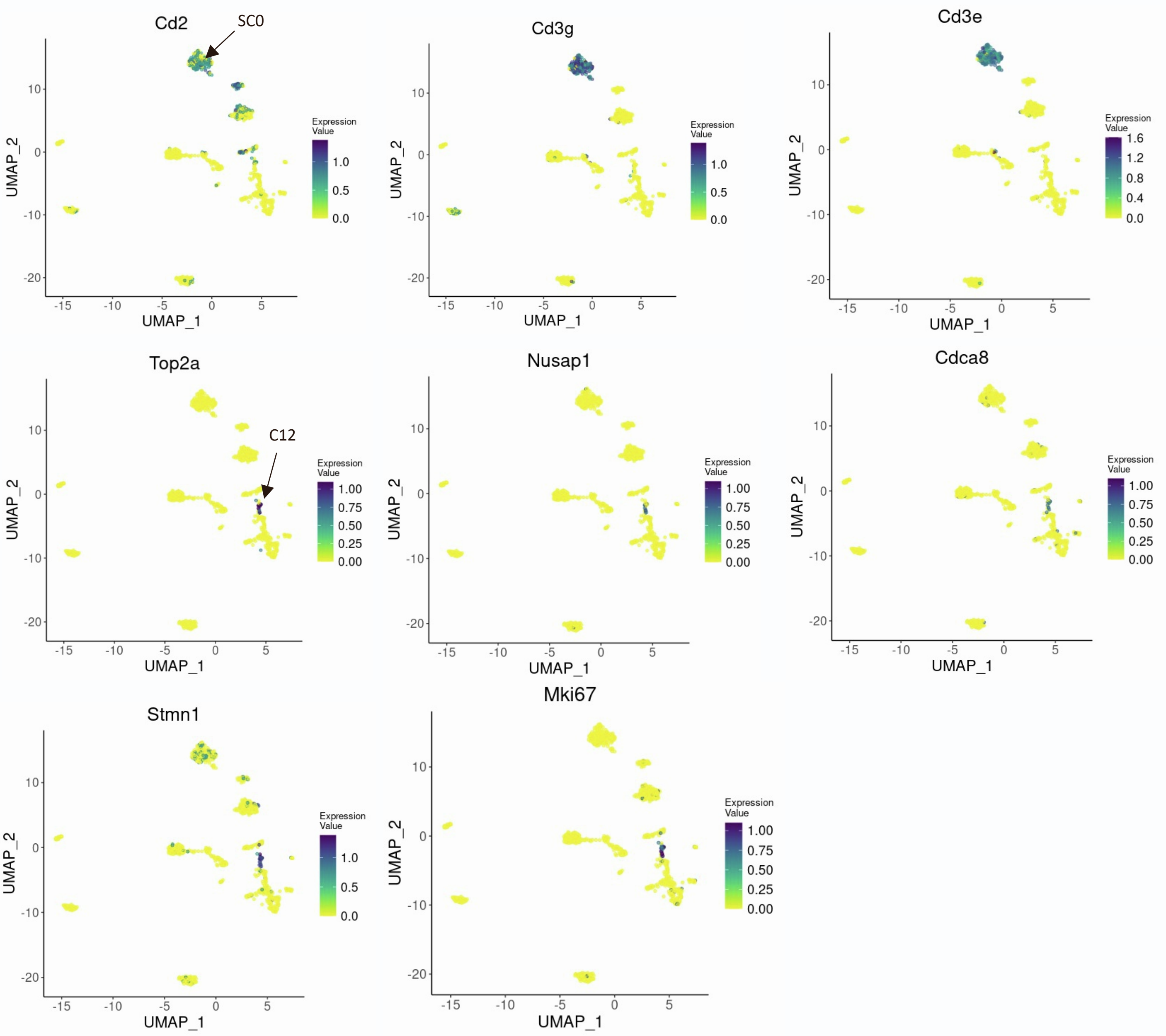

Figure S29. UMAP plots showing the relative distribution of commonly expressed T cell markers (subclusters 0, 12) in the healthy rat immune population subclustering map. Related to Figure 6. Subclusters 0 and 12 were identified as *Cd3*+ T cell populations.The most abundant T cell subcluster (Subcluster 0) was identified based on the enriched expression of *Cd3* genes (*Cd3g, Cd3e, Cd3d, Cd2*) suggesting that this subcluster is a *Cd3*+ T cell population. This T cell subcluster included ribosomal protein genes such as *Rpl12, Lef1*, and *Rps16*. Subcluster 12, the second population characterized as *Cd3*+ T cell, showed enriched expression of γδ T cell gene *Tbx21* (aka T-bet), and phosphoantigen reactive γδ T cell genes *Top2a, Nusap1,* and *Cdca8*. The T cells in this subcluster appeared to be highly proliferative as the most highly expressed DE genes *Stmn1* and *MKi67* which are expressed in proliferating and dividing T cells. Legend for the relative expression of each marker from lowest expression (yellow dots) to highest expression (dark blue dots) is placed on the right. SC: subcluster

NK-like cell markers (subcluster 1, 8) – Immune subclustering

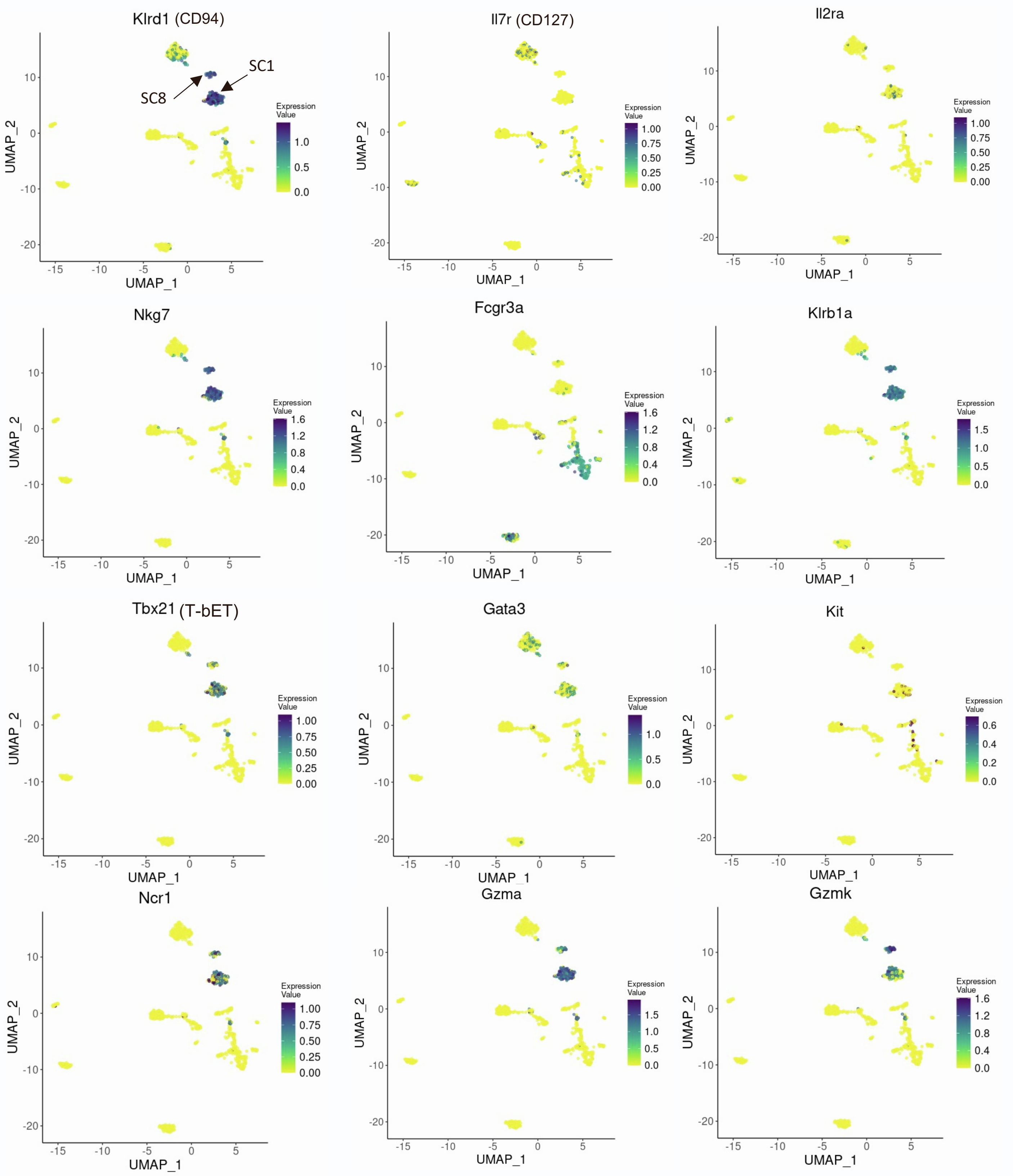

Figure S30. UMAP plots showing the relative distribution of commonly expressed NK-like cell markers (subclusters 1, 8) in the healthy rat immune population subclustering map. Related to Figure 6. Subclusters 1 and 8 were characterized as NK-like cell populations by expression of *Klrd1, Ncr1*, and *Gzmk* without upregulation of *Fcgr3a* or *Itga1*. Expression of *Gzmk* and *Prf1* genes is essential to NK cell identification, as literature has indicated that hepatic NK cells respond to antigens by releasing lytic granules expressing high levels of granzyme and perforin genes. Legend for the relative expression of each marker from lowest expression (yellow dots) to highest expression (Purple dots) is placed on the right. SC: subcluster

B cell markers (subcluster 3) – Immune subclustering

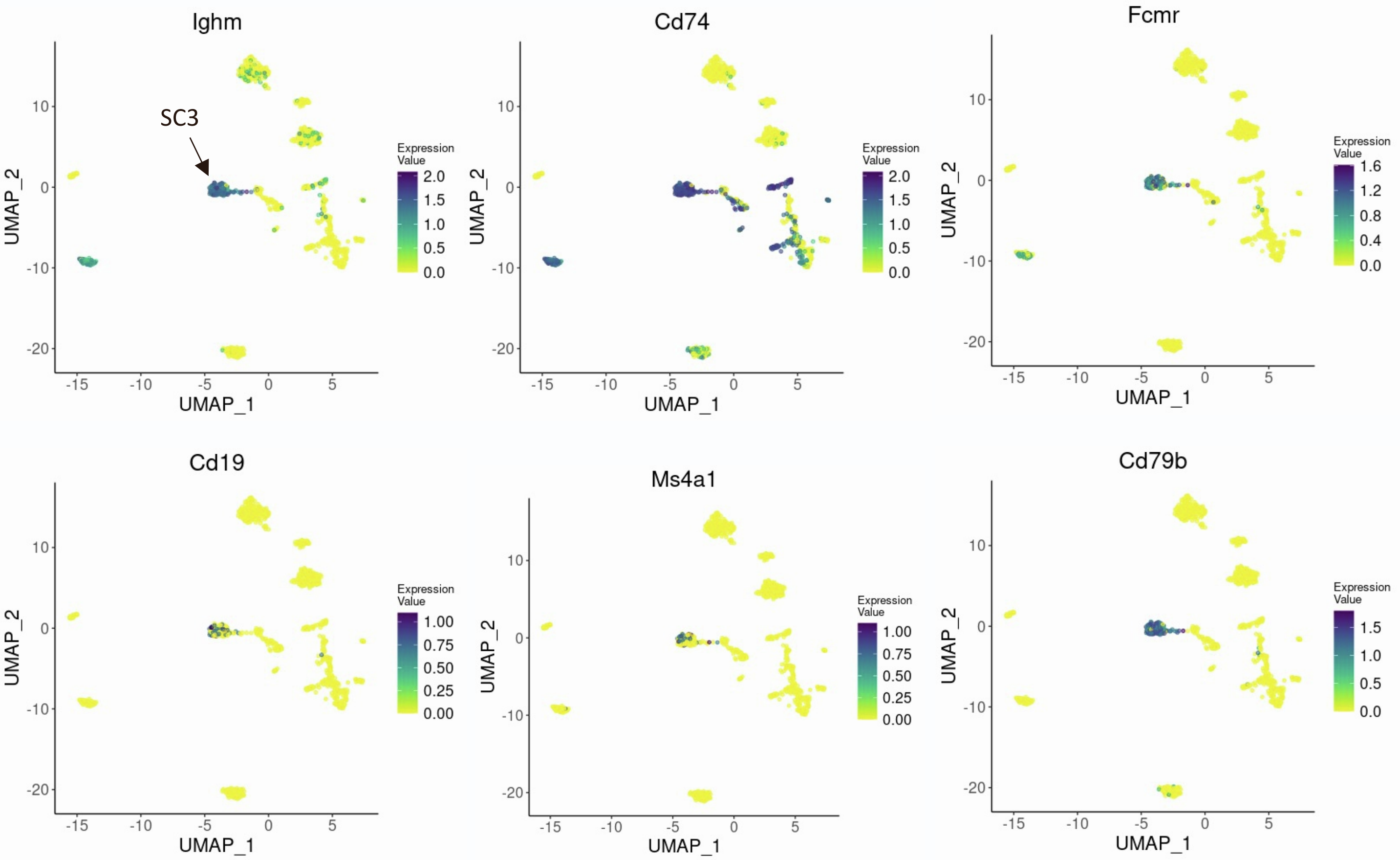

Figure S31. UMAP plots showing the relative distribution of commonly expressed B cell markers (subcluster 3) in the healthy rat immune population subclustering map. Related to Figure 6. Subcluster 3 was characterized as *Cd79b+Ighm+* B cell-like population by enriched expression of *Ighm*, *Cd74, Fcmr, Cd19, Ms4a1(Cd20)*, and *Cd79b*, with no expression of *Ighd* or *Ighg,* suggesting that this subcluster might be Cd19+Cd20+*IgM+IgD-* immature B cells. Legend for the relative expression of each marker from lowest expression (yellow dots) to highest expression (dark blue dots) is placed on the right. SC: subcluster

Myeloid cells markers (subcluster 2, 4, 6, 9, 11, and 13) – Immune subclustering

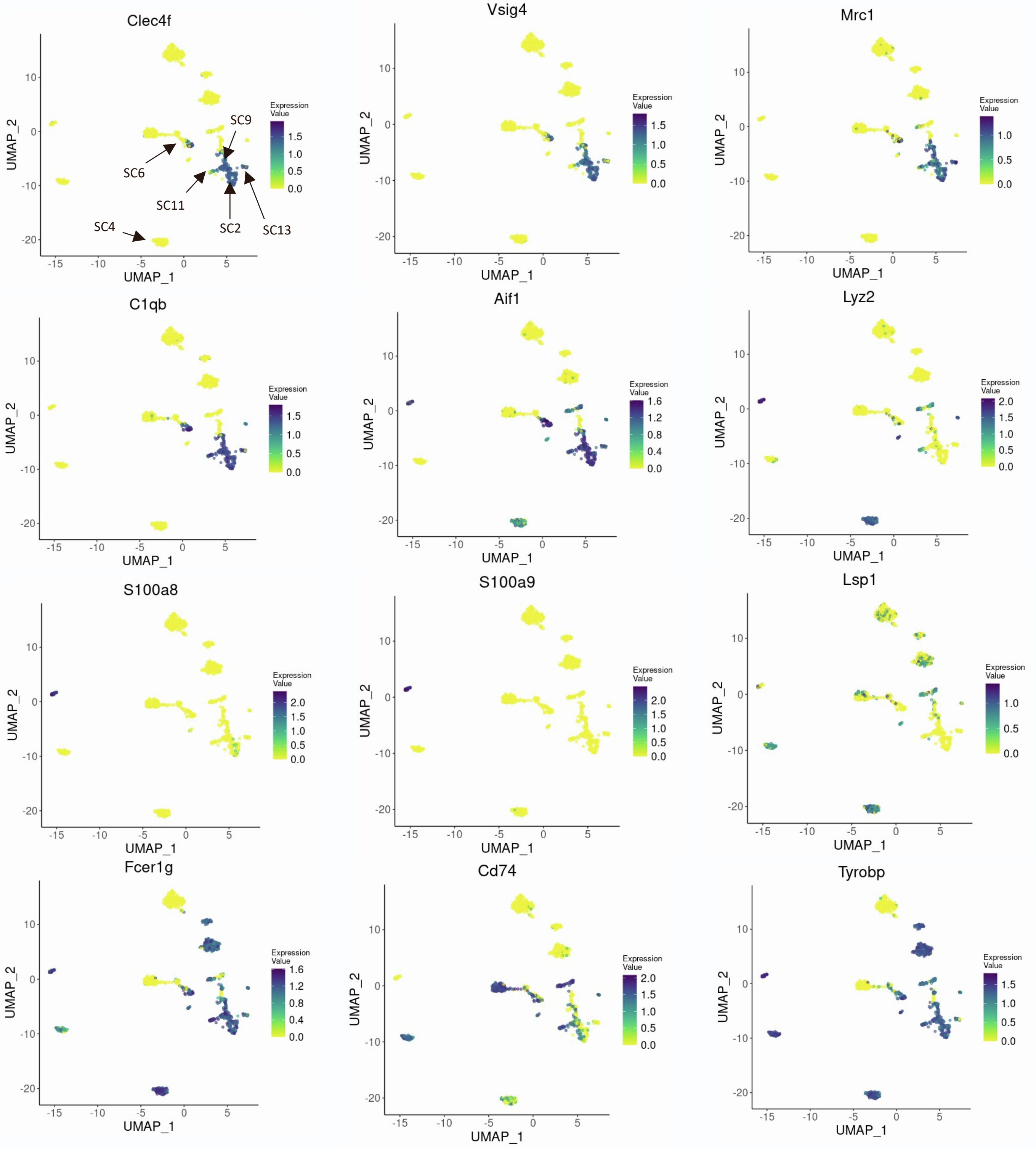

Figure S32. UMAP plots showing the relative distribution of commonly expressed Myeloid cell markers (subcluster 2, 4, 6, 9, 11, and 13) in the healthy rat immune population subclustering map. Related to Figure 6. Subclusters 2, 6, 9, and 13 were identified as myeloid cells based on highly differential expression of *Clec4f*. Subclusters 2 and 13 are identified as *Clec4f*/*Vsig4*+ myeloid clusters based on their top DE genes Subcluster 6 is a predominantly *Clec4f*+ population that appears to be contaminated with hepatocytes, as the top expressed genes include both KC-like myeloid genes *C1qa, Aif1*, and *Clec4f*, and hepatocyte genes *Alb, Serpina1, Hp,* and *Apoa1*. Subcluster 9 is a predominantly *Clec4f*/*Vsig4*+ population that appears to be contaminated with LSECs, showing enriched expression of *Sparc* and *Calcrl,* KC-like genes *C1qb, Mrc1, Vsig4,* and *Aif1* . Subcluster 11 appears to be an intermediate population with an expression of *Fcgr3a* (CD16) and no expression of *Vsig4*. Subclusters 10 were characterized as *Lyz2*+*S100a8*/*9*+ recently recruited macrophages based on the enriched expression of *Lyz2, S100a8*, and *Lsp1*\. Subcluster 4 also showed enriched expression of recently recruited monocyte/macrophage markers *Cd74* and *Tyrobp*. Legend for the relative expression of each marker from lowest expression (yellow dots) to highest expression (purple dots) is placed on the right. SC: subcluster

pDC and cDC markers (subcluster 5,7) – Immune subclustering

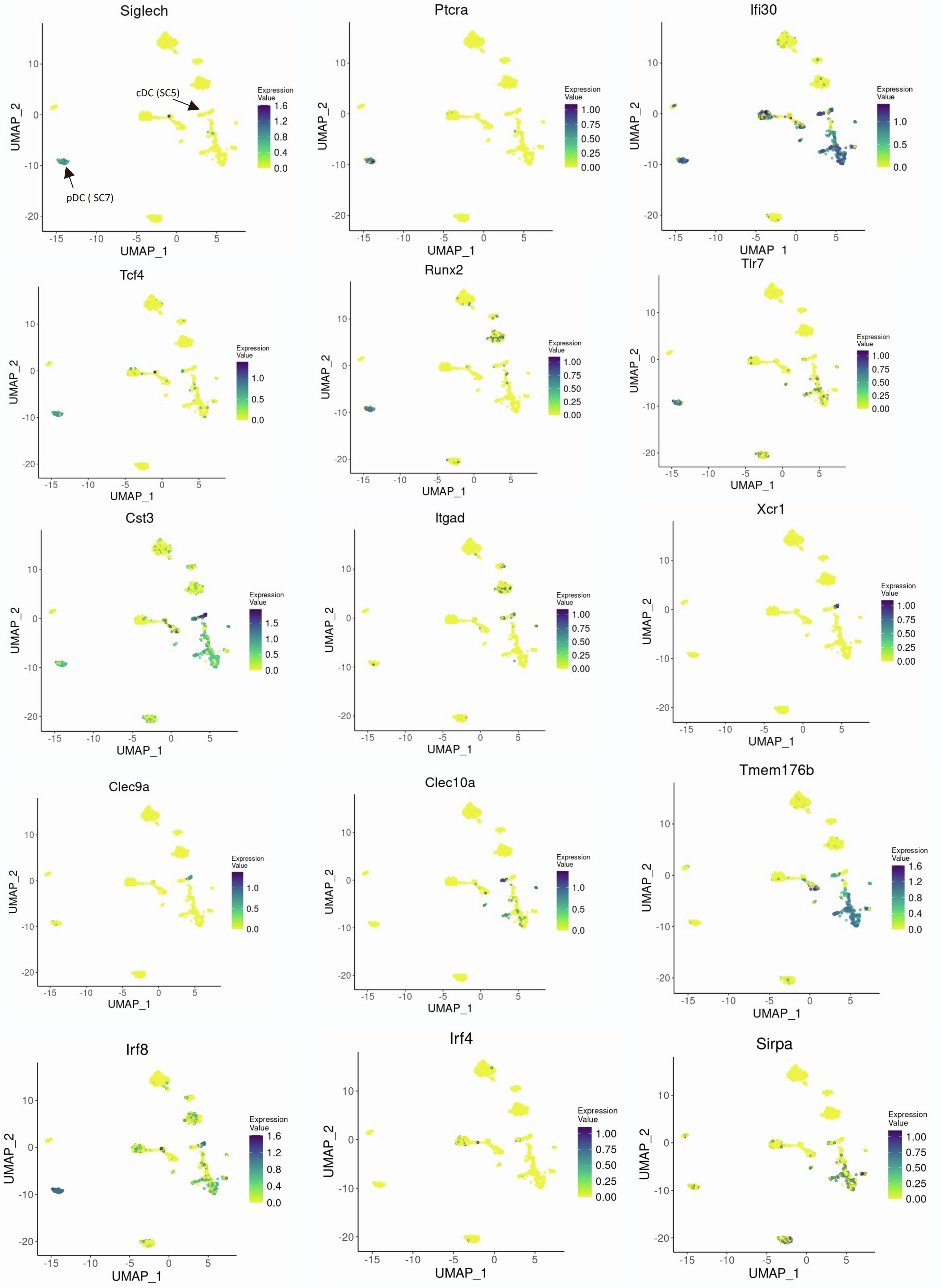

Figure S33. UMAP plots showing the relative distribution of commonly expressed pDC and cDC markers (subcluster 5, 7) in the healthy rat immune population subclustering map. Related to Figure 6. subcluster 7 indicated an enriched expression of DC genes *Siglech, Ptcra, Ifi30, Tcf4, Runx2, Tlr7* and *Ptcra*, suggesting that this subcluster was composed of a pDC population. Subcluster 5 revealed enriched expression of recently recruited monocyte/macrophage markers *Cst3* and *Cd74*, as well as cross-presenting DC markers *Xcr1*, *Clec9a*, and *Tlr3*, suggesting that this subcluster contained a mixture of cDC1 and cDC2 cells. Legend for the relative expression of each marker from lowest expression (yellow dots) to highest expression (dark blue dots) is placed on the right. SC: subcluster

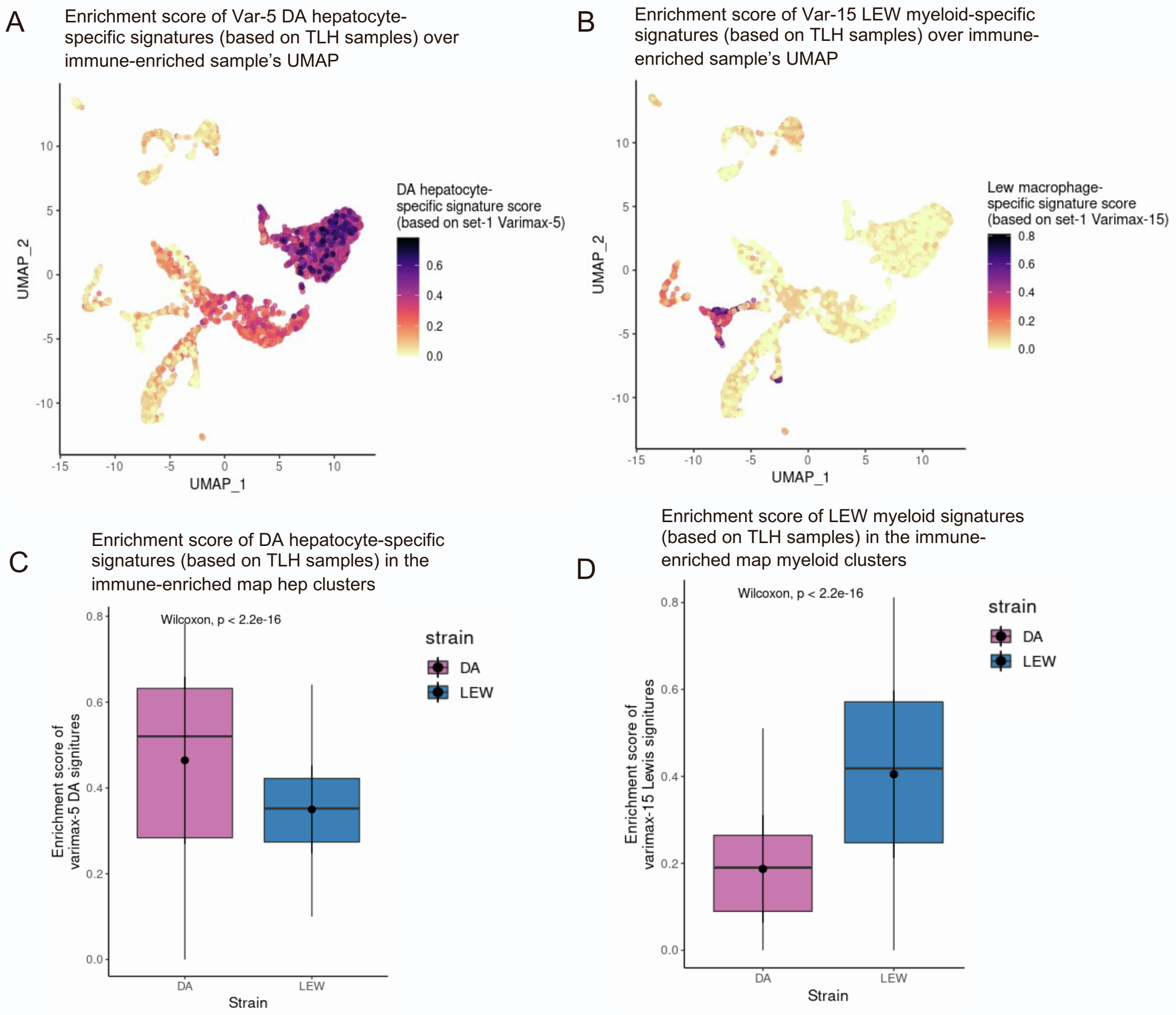

Figure S34. Strain variations in the total liver map are verified in the immune-enriched map. Related to Figures 4, 5 and 6. To verify the hepatocyte and macrophage-specific strain variations (captured by varimax-5 and varimax-15 respectively) identified in the total liver homogenate map, we selected the top 10 positive scoring genes of each factor and calculated their enrichment in each cell within the immune-enriched map using Ucell. Overlying enrichment score of the top 10 A) Hep-specific genes (varimax-5 positive loading gene) and B) myeloid-specific genes (varimax-15 positive loading gene) upon the immune-enriched UMAP. Cells with high enrichment of this geneset are colored dark purple and cells with zero enrichment are indicated as yellow. The distribution of the enrichment scores over the immune-enriched map clusters confirms that varimax-5 and 15 are hepatocyte and myeloid-specific, respectively. Boxplots are indicating the distribution of C) varimax-5's top positive gene enrichment scores within the hepatocyte population and D) varimax-15's top negative gene enrichment scores within the myeloid population of each strain. In line with our predictions based on the TLH map, varimax-5 top positive genes are more enriched in the immune-enriched map DA hepatocyte compared to LEW (Wilcoxon-test p value < 2.2e-16) and varimax-15 top positive genes are more enriched in the immune-enriched map LEW myeloid compared to DA (Wilcoxon-test p value < 0.001). Data are represented as mean ± SEM with each dot representing a single cell.

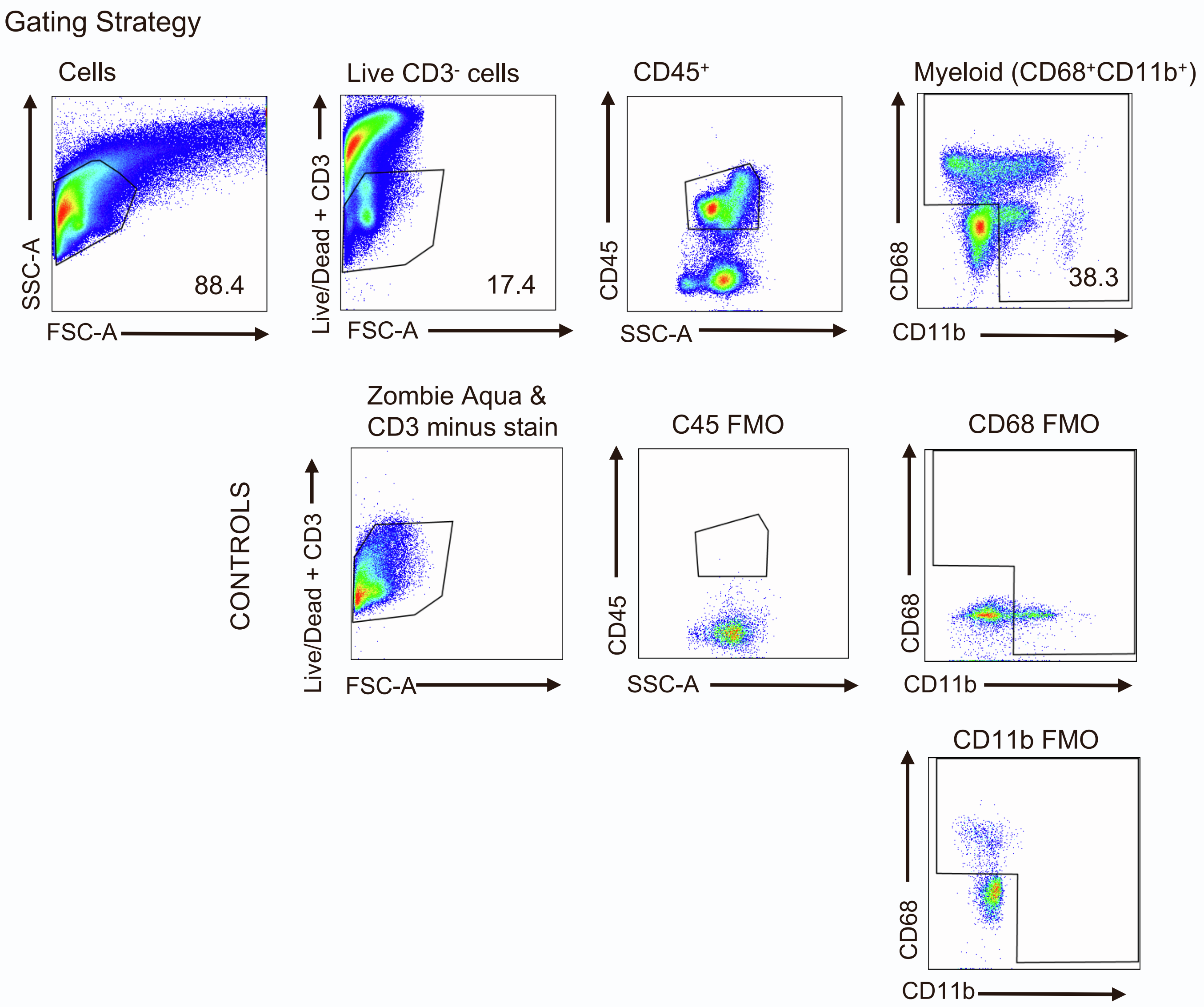

Figure S35. Staining controls for flow cytometry gating strategy. Related to Figure 7. The rationale for the gating strategy was based on partially stained cell suspensions. To analyze a live T cell-free population, cells stained positively for the Live/Dead Zombie Aqua dye and CD3 were gated out. Positivity was determined via a partially stained control that consisted of the full antibody panel minus Live/Dead Zombie Aqua dye and CD3 antibody staining. For all other gating controls (CD45, CD68, CD11b), the fluorescence-minus-one (FMO) staining strategy was used. SSC-A: side scatter area, FSC-A: forward scatter area

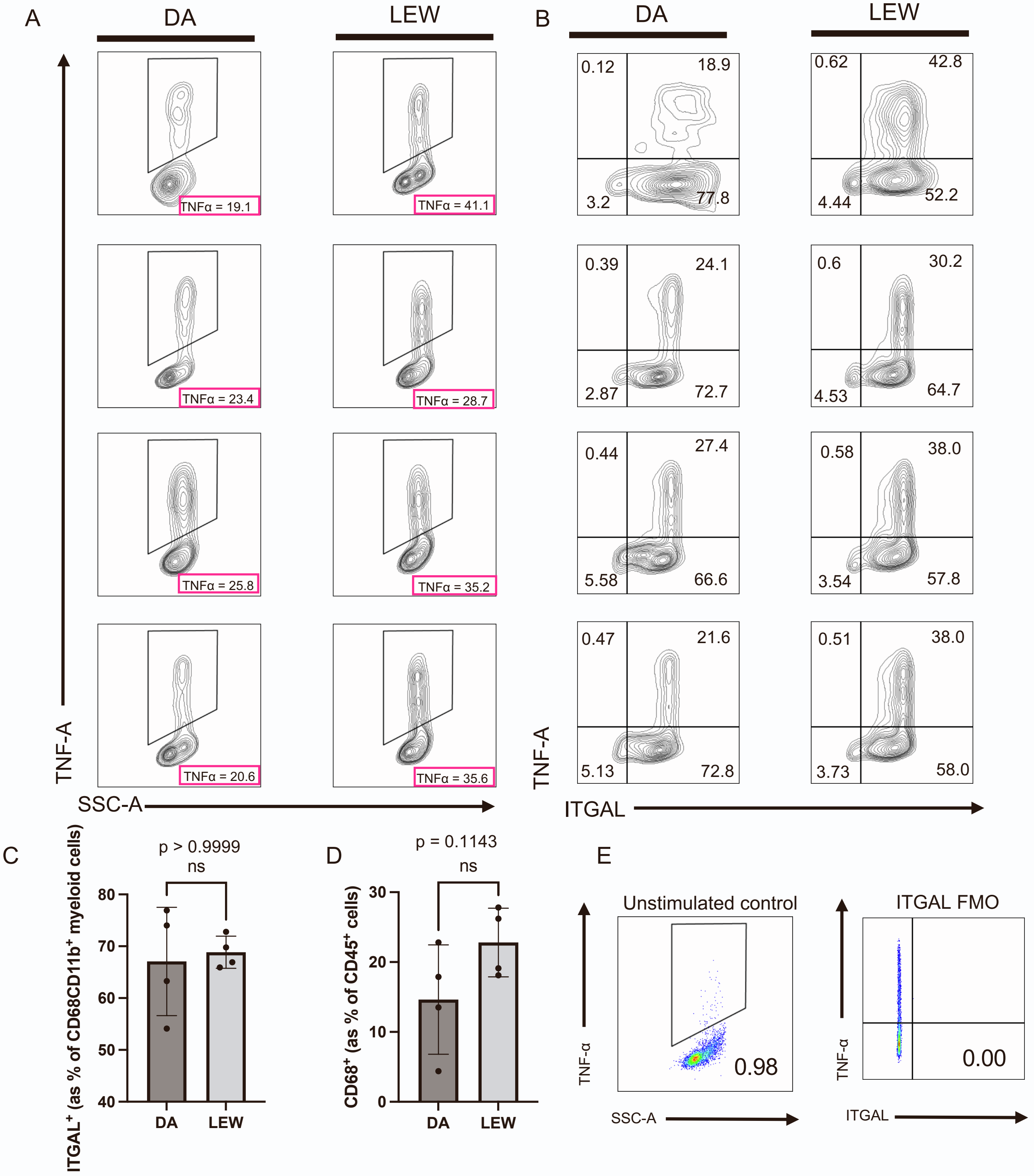

Figure S36. Flow cytometry plots of all Lewis and DA intracellular cytokine stimulation assay replicates. Related to Figure 7. ICS was performed on four animals per strain to investigate the inflammatory potentials of myeloid cells. As described in Figure 7, LPS-induced TNFα secretion was measured via intracellular cytokine staining after being treated with 1ng/mL LPS for 6 hours. Myeloid cells were gated as Live/Dead Zombie Aqua$^-$CD3$^-$CD45$^+$CD68$^+$CD11b$^+$ events. Shown are A) the percentage of total TNFα$^+$, secreting CD68$^+$CD11b$^+$ myeloid cells of all LEW and DA pairs, B) TNFα secretion in ITGAL expressing CD68$^+$CD11b$^+$ myeloid cell subpopulations of all LEW and DA pairs, C) Summary graphs of ITGAL expressing myeloid populations of each strain. Data are represented as mean ± SEM with each dot representing a single animal (n=4). No significant differences between the strains were found. (p>0.9999)

D) Summary graph of CD68⁺ myeloid cells as a percentage of CD45⁺ cells collected at the end of intracellular cytokine assays. No significant differences between the strain were found. ($p>0.1143$), E) controls for TNFα were based on unstimulated cells that were cultured without LPS in parallel to the LPS-treated cells whilst ITGAL staining controls were based on the FMO strategy. Statistical significance was determined using a non-parametric Mann-Whitney (Wilcoxon rank-sum) test.

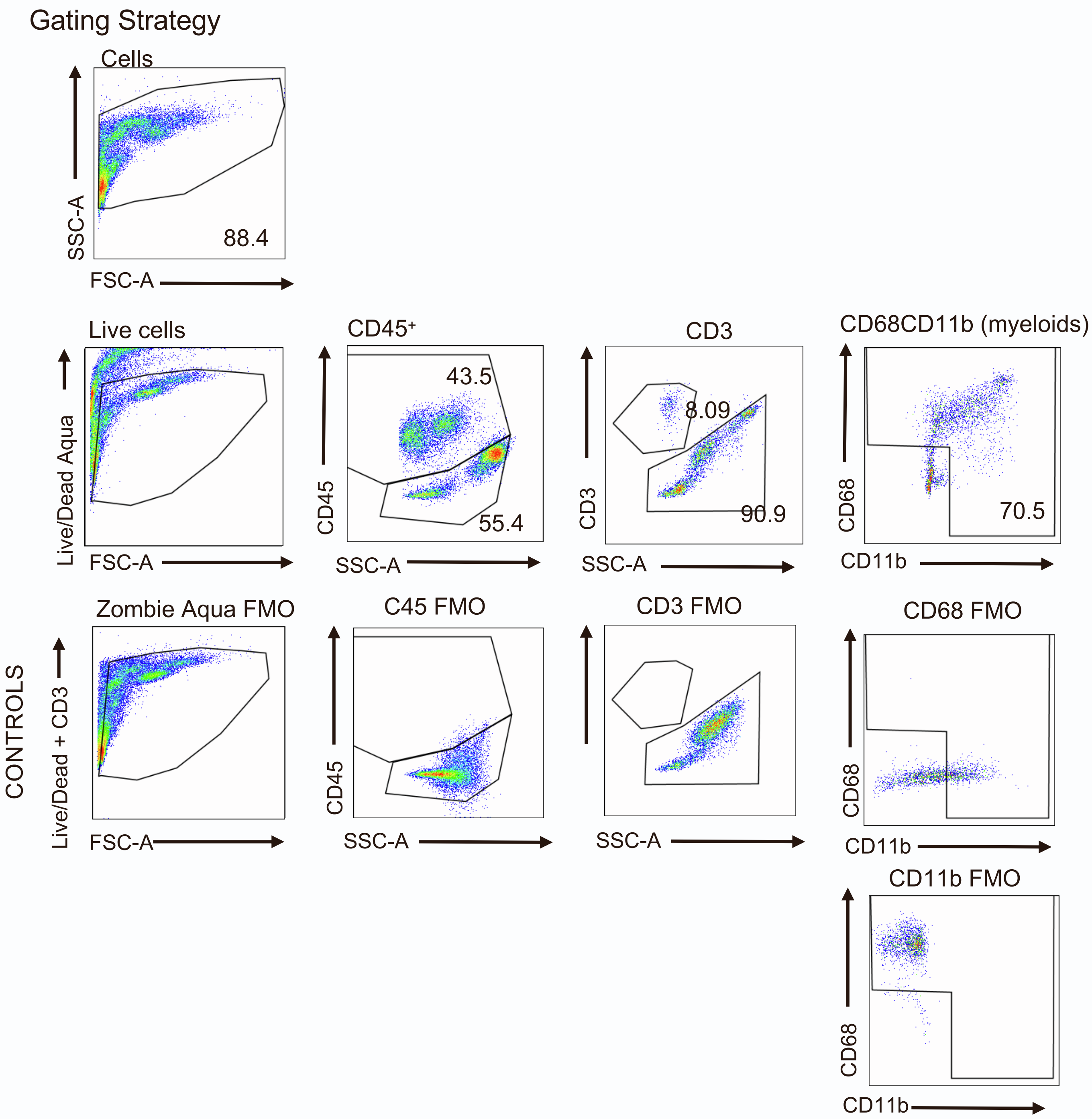

Figure S37. Example gating strategy and staining controls for the verification of myeloid purity post-magnetic bead enrichment. Related to Figure 7. To confirm a live T cell-free myeloid enriched immune fraction was obtained, flow cytometry was performed. Viable cells were determined by excluding positively stained Live/Dead Zombie Aqua cells. Positivity for all markers was determined via fluorescence-minus-one controls (FMO). SSC-A: side scatter area, FSC-A: forward scatter area

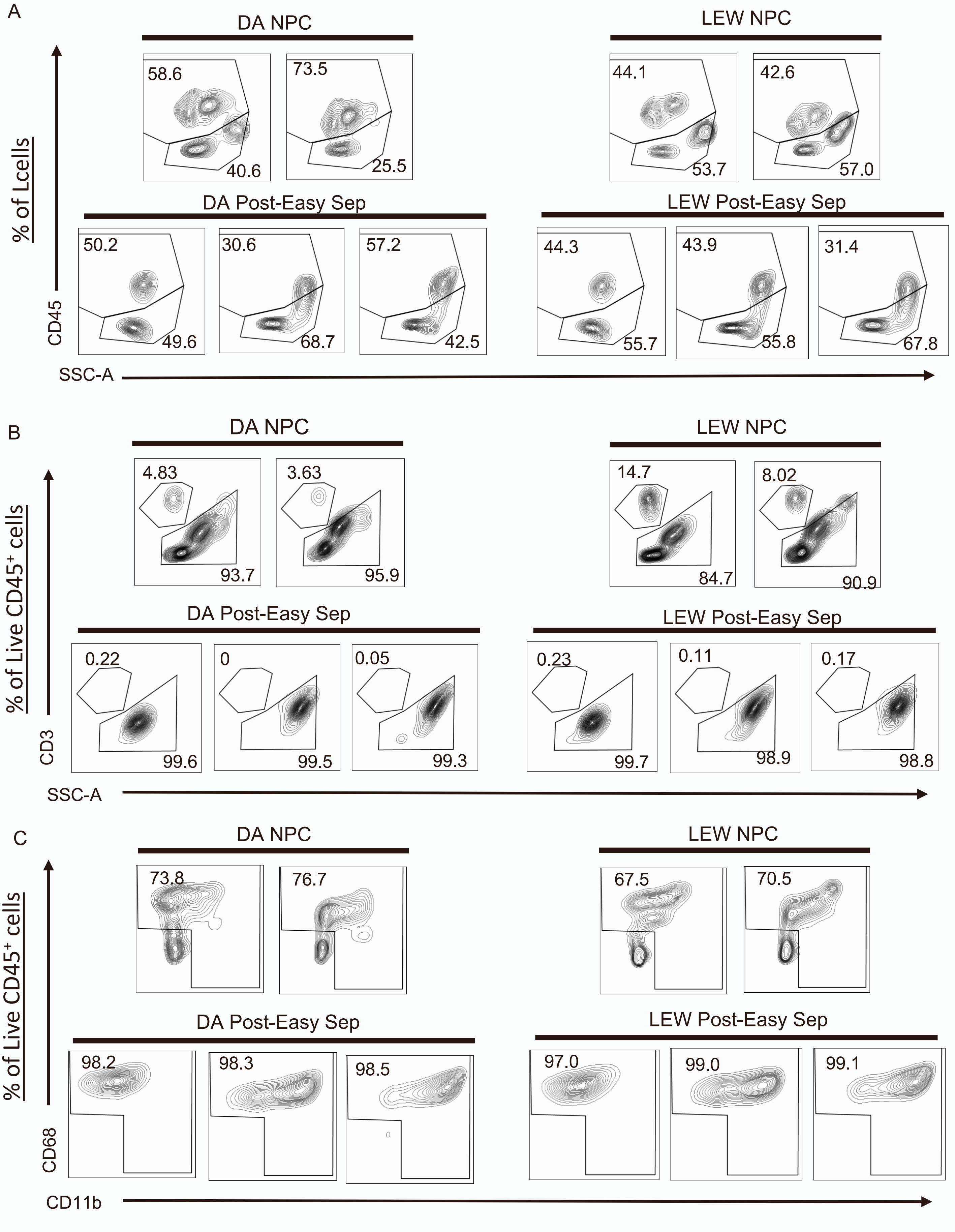

A
DA NPC
58.6
40.6
73.5
25.5
LEW NPC
44.1
53.7
42.6
57.0
% of Lcells
CD45
DA Post-Easy Sep
50.2
49.6
30.6
68.7
57.2
42.5
LEW Post-Easy Sep
44.3
55.7
43.9
55.8
31.4
67.8
SSC-A
B
DA NPC
4.83
93.7
3.63
95.9
LEW NPC
14.7
84.7
8.02
90.9
% of Live CD45+ cells
CD3
DA Post-Easy Sep
0.22
99.6
0
99.5
0.05
99.3
LEW Post-Easy Sep
0.23
99.7
0.11
98.9
0.17
98.8
SSC-A
C
DA NPC
73.8
76.7
LEW NPC
67.5
70.5
% of Live CD45+ cells
CD68
DA Post-Easy Sep
98.2
98.3
98.5
LEW Post-Easy Sep
97.0
99.0
99.1
CD11b

Figure S38. Flow cytometry plots of all Lewis and Dark Agouti NPC and CD68-enriched myeloid immune fractions. Related to Figure 7. To confirm cell suspensions were depleted of non-myeloid immune cells after magnetic-bead CD68 enrichments, flow cytometry was performed on all pairs of Dark Agouti and Lewis non-parenchymal liver cell (NPC) suspension and post-enrichment cells suspensions(n=3). Myeloid cells were gated as Live/Dead Zombie Aqua^{-}CD3^{-}CD45^{+}CD68^{+}CD11b^{+}. Shown are A) depletion of CD45 heterogeneity post-enrichment. B) depletion of T cells post-enrichment C) percentage of CD68^{+}CD11b^{+} myeloid cells amongst all immune cells defined as live CD45^{+} cells. SSC-A: side scatter area.

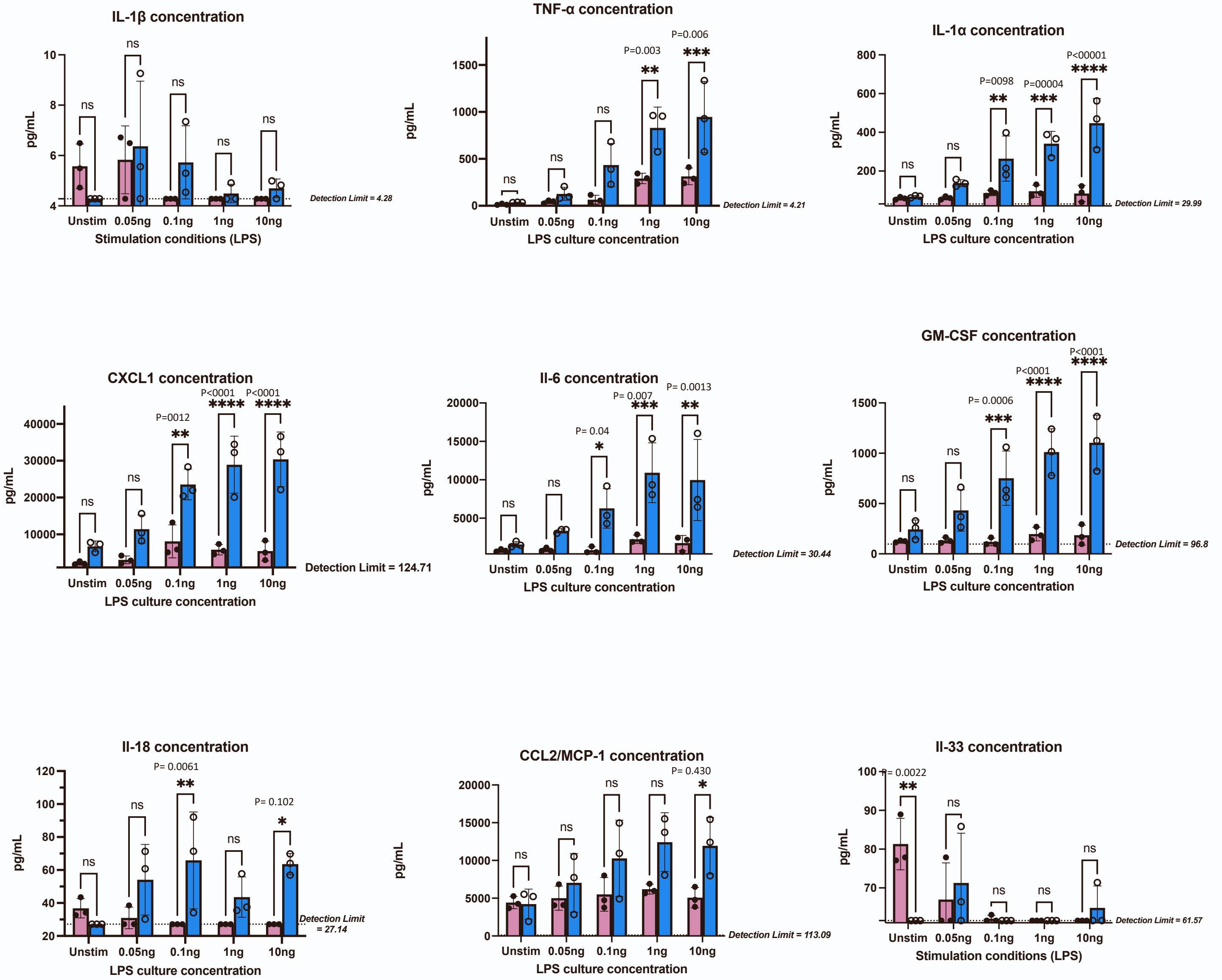

Figure S39. Inflammatory cytokine concentrations were significantly higher in Lewis myeloid cultures compared to Dark Agouti's. Related to Figure 7. To further validate transcriptional strain differences of the inflammatory potential of myeloid cells, and the findings from the ICS, a multiplex (12-plex) cytometric bead array (LEGENDplex) was performed on the culture supernatants of LPS-stimulated (0, 0.05, 0.1, 1, 10 ng/mL) myeloid cultures from three pairs of animals. Significantly higher inflammatory cytokine secretion was found in Lewis Cd68^{+} myeloid cells for eight of the detectable cytokines(TNFα, Il-6, Il-18, GM-CSF, CXCL1, CCL2 and Il-1α). Data are represented as mean ± SEM with each dot representing a single animal. (n=3) Three technical replicates were used per animal. Statistical significance was determined using a two-way ANOVA and Šídák's multiple comparisons test. (* : p-value < 0.05, ** : p-value < 0.01, *** : p-value < 0.001,**** : p-value < 0.0001)

Cluster	Mt-thr=20	Mt-thr=30	Mt-thr=40
Hep (0)	4989	4989	4989
Hep (1)	4212	4212	4212
Marco/Cd5l Mac (10)	518	518	518
Endothelial (11)	517	517	517
Hep (12)	446	446	446
Lymphocyte (13)	409	410	410
Mesenchymal (14)	265	265	265
Hep (15)	167	167	167
Hep (16)	76	126	127
Hep (2)	1182	2497	2606
Endothelial (3)	2090	2091	2091
Hep (4)	1670	1739	1740
Marco/Cd5l Mac (5)	1149	1150	1150
Hep (6)	1123	1124	1124
Mesenchymal (7)	983	984	984
Hep (8)	753	949	977
Lyz2/Cd74 Mo/Mac (9)	713	713	713
Total	21262	22897	23036

Table S2. Total liver homogenate map clusters are robust to the choice of MT-fraction threshold. Related to Figure 1. Table representing the cell count of final TLH map clusters at various mitochondrial cut-offs. The number of cells in each cluster (resolution: 0.6) was evaluated for three different mitochondrial fraction thresholds to ensure that our map was robust at all mitochondrial cut-offs. For this analysis, all samples were filtered using the noted harmonized threshold in the column name. In all mitochondrial cut-offs (40%, 30%, 20%), we have cells from all 17 clusters identified.
